# Pore Architecture and Dynamic Ionic States in Solid and Quasi-Solid Electrolytes for Lithium, Sodium, and Zinc Batteries: Evidence and Testable Criteria for Ionic Memory

**DOI:** 10.3390/ma19214505

**Published:** 2026-10-26

**Authors:** Ammar Alsoud, Alexandr Knápek

**Affiliations:** 1 Department of Physics, Faculty of Science, Isra University, Amman 16622, Jordan; 2 Department of Physics, Faculty of Electrical Engineering and Communication, Brno University of Technology, Technická 2848/8, 616 00 Brno, Czech Republic; 3 Institute of Scientific Instruments, Czech Academy of Sciences, Královopolská 147, 612 00 Brno, Czech Republic

**Keywords:** ion association, desolvation, counterion immobilization, coordination exchange, percolation, transference number, operando characterization, electrochemical hysteresis

## Abstract

Porous electrolytes are commonly discussed through pore size, surface area, conductivity, and cell lifetime, although these measurements do not by themselves identify the conducting phase or ionic pathway. This critical narrative review examines how architecture regulates ion-specific states in lithium, sodium, and zinc electrolytes. We distinguish solvent-free solids from solvent-containing composites, gels, ionogels, and hydrated networks, then evaluate evidence connecting pore accessibility and wall chemistry to salt speciation, counterion retention, residence time, and transport. Published observations are separated from mechanistic interpretations and prospective hypotheses. The synthesis motivates a state-aware framework whose descriptors are organizing tools, not validated universal laws. Nanofluidic and iontronic demonstrations provide device-specific precedents for ionic memory; they do not establish memory in bulk battery electrolytes. A unified write–wait–read–erase protocol and an evidence matrix distinguish dynamic ionic gating, history-dependent transport, persistent reversible ionic states, and programmable ionic memory from irreversible conditioning and artifacts. The proposed extension to bulk battery electrolytes remains an open experimental question requiring field-off retention, minimal disturbance of readout, reversible switching, and controls locating the state within the electrolyte.

## 1. Introduction

A scientific challenge is to identify which physical region actually carries ions in a material described as porous. An empty connected channel, a solvent-filled pore, a functionalized framework, and an infiltrated ceramic scaffold can all improve an electrochemical measurement by different routes. Conductivity reports their combined response together with electrode contacts; it does not by itself separate a fast bulk path from increased liquid content or improved interfacial wetting. For lithium, sodium, and zinc electrolytes, the first mechanistic question is therefore one of location and physical state rather than performance ranking. Broader solid-state Li and Na electrolyte surveys frame the interface and material-class context [1,2,3,4,5,6,7,8].

A second question concerns the carrier. The species approaching a constriction can be solvated, polymer-coordinated, paired with an anion, or associated with a charged wall. These populations have different effective sizes and exchange rates even when the nominal transported ion is Li^+^, Na^+^, or Zn^2+^. We consequently treat salt speciation and residence time as variables that may change with pore chemistry, temperature, water activity, and electrical history. A pore-size comparison without a carrier-state measurement has limited power to establish a selective transport mechanism.

Pore networks introduce a further distinction between geometric continuity and dynamic interaction. A sample-spanning route can conduct without producing a persistent state, while a slowly relaxing site may influence only its local neighborhood. The present review evaluates whether evidence for one property also supports the other. This distinction matters when a conductivity maximum is attributed to percolation or when a delayed current is interpreted as collective transport; both assignments require controls beyond a loading series [9].

Physical-state reporting is central to the comparison. Retained organic solvent, ionic liquid, and water can be essential to a useful quasi-solid cell, but they alter the identity and abundance of mobile species. A dry polymer framework, a gel-filled membrane, and a hydrated Zn network should therefore not be treated as interchangeable examples of solid-state ion conduction. Their cell functions can be compared only after the conducting phase and measurement conditions are made explicit [10,11,12,13,14,15,16,17,18]. Hydrogel reviews emphasize that water content, polymer chemistry, and Zn-ion regulation must be reported together [19,20,21,22].

The same caution extends to interfaces. A porous region may reduce local current focusing and accommodate deposition but also expose reactive area or enable void and crack formation. Cell lifetime depends on contact pressure, current density, areal capacity, metal inventory, and interphase evolution. Images of smoother metal deposition are informative but cannot alone identify the pathway by which the electrolyte transports ions [23,24,25,26,27,28]. We use such observations to connect structural mechanisms to application-specific tests rather than to infer a universal optimal porosity. Sodium interface engineering and its failure modes have also been reviewed [29].

The contribution of this critical narrative review is a claim-dependent evidence hierarchy linking architecture, carrier identity, and response time. Structural characterization establishes location and accessibility; coupled spectroscopy and transport constrain the ion-bearing population; time-dependent measurements test exchange and retention. Established ingredients such as confinement, selective coordination, and relaxation are synthesized from published studies. The additional connection between these ingredients and a persistent, programmable bulk ionic state is proposed for testing. Neither the individual mechanisms nor the conceptual ratios introduced later are claimed as a new universal theory. Their intended value is to identify discriminating experiments and the conditions under which an interpretation should be rejected.

The prospect of history-dependent transport motivates a final boundary. A loop in a voltage sweep may arise from capacitive polarization, concentration gradients, heating, electrode reactions, or irreversible changes. Iontronic and nanofluidic systems demonstrate device-specific electrical-history responses [30,31,32], while analogous evidence in bulk battery electrolytes remains a separate experimental question. Throughout this review, ionic memory requires a distinguishable ionic state retained after a field-off waiting interval, read with minimal disturbance, and reversibly erased or rewritten. The operational sequence is write–wait–read–erase (WWRE); the waiting interval tests retention rather than constituting an additional electrical stimulus.

The review proceeds from the physical inventory of pore types to the evidence for transport and interfaces, then to speciation, trapping, connectivity, and temporal response. The illustrations provide structural and battery context, motivate the state variables, and organize the memory evidence and proposed tests. The purpose is to define what current results support and which measurements could reject the proposed link between architecture and a dynamic ionic state.

Three evidence categories are maintained throughout: reported observations in the cited systems, mechanistic interpretations supported to the extent that competing explanations were tested, and prospective hypotheses requiring new validation. Section 3, Section 4, Section 5, Section 6, Section 7, Section 8, Section 9 and Section 10 synthesize the first two categories. Section 11, Section 12 and Section 13 identify the proposed extension and its countertests. No bulk-battery-electrolyte system is designated here as a validated programmable ionic memory solely on the basis of conductivity, cycling, hysteresis, or delayed relaxation.

**Figure 1:**
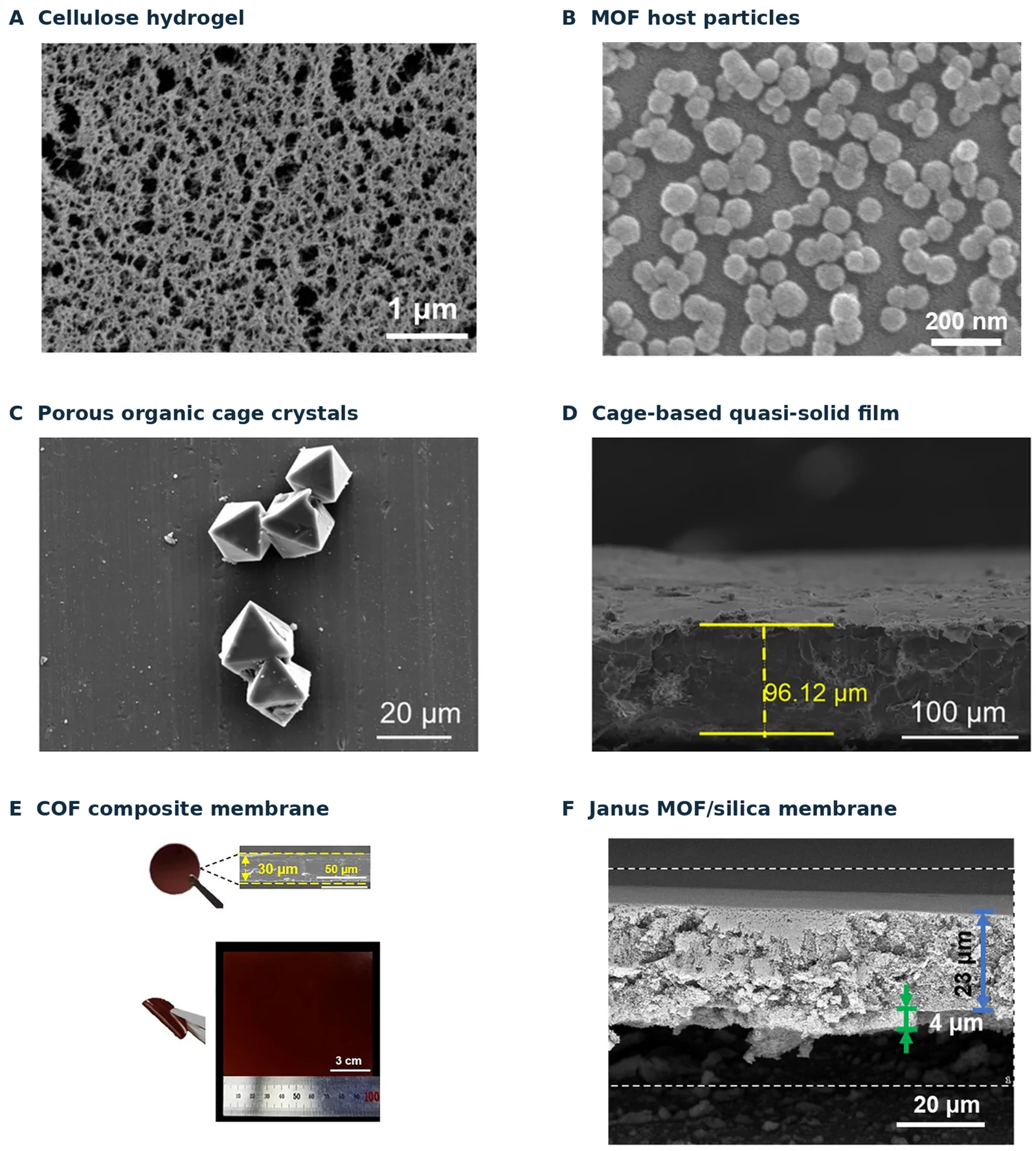
Published experimental morphologies. (**A**) Cross-sectional FE-SEM of freeze-dried DCZ cellulose hydrogel: Zhang et al. [33], Figure 1d in Ref. [33]. (**B**,**F**) MOF particles and a Janus MOF/silica membrane cross-section: Chen et al. [35], Figure 1a,g in Ref. [35]. (**C**,**D**) CC3 crystals and a cage-based quasi-solid electrolyte cross-section: Qin et al. [37], Figure 2a,b in Ref. [37]. (**E**) COF composite membrane photographs and cross-sectional SEM detail: Li et al. [36], Figure 1e in Ref. [36]. Cropped, uniformly resized, and rearranged with Python 3.12.14 and Pillow 12.3.0; original scale bars and dimensional annotations retained. All four sources: © The Author(s) 2024, CC BY 4.0, https://creativecommons.org/licenses/by/4.0/ (accessed on 19 October 2026). SEM morphology does not resolve framework micropores or establish operating-state transport.

Figure 1 provides published examples of host particles, hydrogel morphology, and fabricated electrolyte membranes within the architecture classes discussed below [33,34,35,36,37,38,39,40,41,42]. The physical state and specimen preparation must accompany the images: a dried section or precursor-powder image does not establish the ion-conducting pathway in an operating membrane.

## 2. Literature Selection, Evidence Grading, and Physical-State Audit

This article is a critical narrative review and an evidence-based conceptual synthesis, not a systematic review or meta-analysis. The initial bibliography was assembled iteratively around material architectures and mechanistic questions; a reproducible database search log and screening counts are not available for the initial collection. Consequently, no claim of exhaustive coverage, preregistration, or PRISMA-style screening is made.

A targeted revision-stage search was conducted on 16–17 October 2026 using a general web search to identify publisher-hosted studies, with DOI-directed checks against publisher pages and Crossref bibliographic records. No Scopus, Web of Science, or other subscription-database search is claimed. There was no fixed lower publication-date boundary; older methodological studies were retained where directly relevant, and recent experimental evidence was sought through the revision date. Exact-title and DOI searches were used to resolve incomplete records and the studies suggested by the reviewers.

Representative revision queries were: “porous solid electrolyte” lithium sodium zinc “transport”; “solid” “quasi-solid” electrolyte “ionic memory”; “electrolyte” “BET” “solvent” “MOF” 2025 2026; and “nanofluidic memristors” “electrode artefacts”. Additional exact DOI queries were 10.1093/nsr/nwac024, 10.1016/j.jechem.2022.04.029, and 10.1016/j.cej.2026.177663. These targeted queries document the update, not a retrospectively reconstructed search history for the initial collection.

For mechanistic discussion, studies were selected when they connected an identified electrolyte or confined-ion architecture to measured transport, species/coordination evidence, interfacial behavior, or a temporal-state test. Primary research was preferred for quantitative entries; reviews provided context and routes to original studies. Electrode-only porosity, gas-storage results, and iontronic devices were included only as explicitly identified methodological comparisons or analogies. They were not treated as direct proof of battery–electrolyte transport or memory.

Records with unresolved identity were not used to support new quantitative additions. Unverified numerical details, unmatched physical states, and inaccessible supporting data were excluded from direct rankings rather than imputed. Duplicate DOI records were checked, and the accessible source was used to distinguish observation from the original authors’ interpretation. Bibliographic metadata verification establishes source identity; it does not independently validate experimental measurements or mechanistic claims. The targeted search and narrative selection remain susceptible to coverage and selection bias.

Figure preparation and AI assistance. ImageGen (OpenAI; hosted service, build identifier not recorded) assisted preparation of the original artwork; the thematic map retains an AI-assisted qualitative thematic map. The experimental and conceptual composite illustrations were assembled with Python 3.12.14 and Pillow 12.3.0 from individually attributed published panels and the original conceptual elements identified in their captions. The nanoconfinement illustration contains published molecular-dynamics results; The Li-COF transport illustration contains published experimental measurements. The conceptual network in the connectivity illustration does not represent a measured or simulated pore topology. The research-agenda illustration was prepared with Python 3.12.14 and Matplotlib 3.10.8 as an original synthesis of testable propositions. Captions distinguish experimental observations, source calculations, and original concepts, identify exact source panels, and state reuse conditions and layout changes. Artwork from reference [9], whose archived license restricted adaptation, is not reproduced. Codex (OpenAI; hosted service, build identifier not recorded) assisted revision-stage language editing, code preparation, literature/reference organization, and figure-source matching. This assistance did not generate the reproduced experimental panels or source simulation results. The authors are responsible for verifying scientific interpretations, bibliographic details, source fidelity, and reuse compliance. In the final proof correction, selected published panels were reassembled from higher-resolution source files; axis typography was corrected without redrawing the data. Matplotlib 3.10.8 was used to typeset the corrected axis labels in the Li-COF transport illustration. The three-dimensional conceptual artwork in Figure 7C was prepared with ImageGen (OpenAI; hosted service, build identifier not recorded) and reviewed by the authors; it represents no experimental or simulated dataset.

The literature was read through the mechanism claimed by each study rather than through a single ranking of material performance. For each system, we record the nominal ion chemistry, the pore-bearing component, the operating physical state, and the measurement used to assign a transport pathway. This approach makes it possible to compare a dry framework with a liquid-filled membrane without describing both as instances of the same solid conduction mechanism.

The bibliography covers several distinct experimental traditions, including polymer composites, frameworks, ceramics, interfaces, and nanofluidic devices. References are used to test specific propositions in the text; their total number is not itself evidence of systematic coverage. The quantitative framework-electrolyte comparisons are presented with the reported temperature and liquid inventory because a conductivity value separated from those conditions is an unreliable basis for comparison.

We distinguish an observed association from a mechanism identified by an intervention. Imaging can locate voids; spectroscopy can constrain local coordination; transport and transference measurements establish an electrical response. A pore-specific causal claim becomes stronger when access to the internal channel, rather than simply filler amount, is varied while the surrounding chemistry and mobile-phase content are held sufficiently similar. No one technique supplies all of these pieces.

A physical-state classification is assigned at the measurement condition. Solvent-free solids, partially solvated solids, gels, ionogels, and hydrated zinc networks are separated according to the mobile phase actually present. The word quasi-solid identifies mechanical form but cannot specify whether ions move mainly through polymer segments, a confined liquid, or a framework. Where retained liquid or water is not quantified, that uncertainty remains in the comparison rather than being silently resolved.

The resulting synthesis is qualitative because published studies differ in cell geometry, test pressure, temperature, salt content, current density, and definitions of failure. We therefore use numerical cross-study comparisons to reveal reporting gaps and candidate trends, not to calculate a pooled conductivity advantage. Mechanistic claims receive priority when a reported structural variable has a matched electrical or chemical observable and an alternative pathway was examined. Life-cycle criteria are a further practical constraint when comparing battery architectures [43].

Section 3, Section 4, Section 5, Section 6, Section 7, Section 8 and Section 9 review material architectures and battery consequences; Section 10 evaluates ionic and temporal evidence; Section 11, Section 12 and Section 13 propose and challenge a state-aware framework. This order separates reports of improved performance from the later question of whether the electrolyte can retain an electrically written ionic state. Proposed memory mechanisms are identified as hypotheses unless field-off, reversible, and bulk-attributed tests support them.

Terminology is used operationally throughout. Dynamic ionic gating denotes a response controlled during stimulation; history-dependent transport denotes a subsequent response influenced by prior excitation without, by itself, establishing memory. A persistent reversible ionic state additionally requires field-off retention and reversible state recovery. Programmable ionic memory requires reproducible WWRE operation and ionic attribution in the relevant specimen. Irreversible conditioning denotes a retained chemical, structural, or contact change that cannot be reversibly reset under the stated protocol. The state-aware framework is the authors’ conceptual synthesis; it is not a validated constitutive model. Table 1 summarizes this claim-dependent evidence hierarchy.

**Figure 2:**
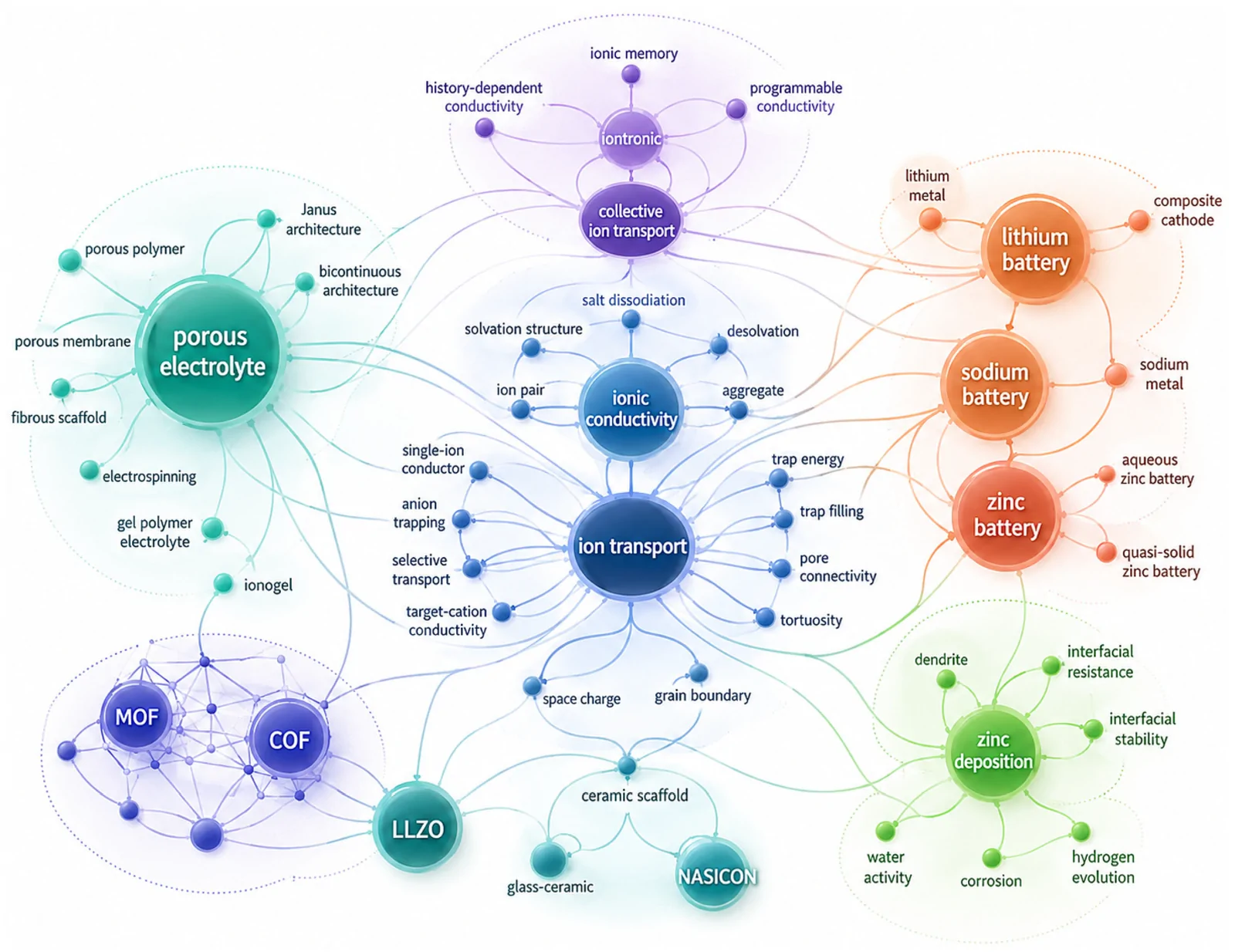
Qualitative thematic map connecting electrolyte architectures, ion transport, battery chemistries, interfaces, and prospective ionic memory [44,45,46,47]. Node sizes and links are illustrative and do not represent a bibliometric analysis. ImageGen (OpenAI) assisted figure preparation. Links indicate thematic association, not demonstrated causal coupling or a progression toward memory. Colors group related themes; the colored links and arrows are illustrative thematic connections and do not encode measured direction, magnitude, or causality.

Figure 2 provides a qualitative reading map for the themes of the review; it is not derived from a quantitative bibliometric network.

## 3. Operational Taxonomy: Where Are the Pores and Which Phase Carries the Ions?

### 3.1. Intrinsically Porous Polymer and Fibrous Hosts

The most useful first distinction for a porous polymer is whether the polymer itself supplies the continuous ion-bearing pathway. A fibrous skeleton can remain mechanically coherent while its voids contain a second phase; the electrical current may then follow that phase rather than the dry polymer. Conductivity before and after controlled filling, together with mass uptake and through-plane measurements, provides a more informative description than pore size alone. Porous asymmetric polymer supports and porous-net templates illustrate the separate roles of support and conduction [48,49].

Flexibility and interfacial conformity often motivate fibrous separators. These advantages can improve a cell even when they do not increase intrinsic ionic mobility. A fair account reports membrane thickness, accessible porosity after processing, and the amount of liquid or water retained during the transport measurement. Improved wetting should be described as an architectural function rather than automatically as a new solid conduction channel [17,18].

Chemical groups on a pore wall offer a separate intervention. Fixed charge or polar coordination sites may alter counterion residence and the local cation environment, but a shift in a vibrational band does not show that the altered population carries a through-thickness current. Pairing species-sensitive measurements with diffusion or transference tests is essential to distinguish selective regulation from a nonspecific change in salt loading. Analogous ion selectivity in non-battery membranes is useful only as a comparison, not battery validation [50].

### 3.2. Dense Polymers Containing Porous Fillers or Connected Porous Networks

When porous particles are dispersed in a dense polymer, the voids belong to the filler rather than the continuous matrix. Three candidate routes must be considered: motion in the polymer, motion near particle surfaces, and passage through accessible internal channels. If particles are isolated, a conductivity change can arise from polymer disorder without an internally connected pore route. A nonporous analogue and a controlled pore-accessibility treatment would help separate these possibilities. MOF-containing single-ion fibrous membranes and other composite hosts exemplify the mixed-path problem [51,52].

Increasing filler content changes several variables simultaneously: interparticle distance, interfacial area, crystallinity, mechanical response, and sometimes residual solvent. A conductivity maximum is therefore not an unambiguous percolation threshold. The structural network should be measured independently, and the electrical trend should be checked against mobile-ion inventory and polymer relaxation before a continuous filler-assisted pathway is assigned.

### 3.3. Self-Conducting Porous Frameworks

Frameworks supply chemical precision that an unfunctionalized void lacks. Charged channel walls, tethered anions, open sites, and discrete cages can alter the partitioning of cations and anions. The key comparison is between the structure of the empty host and the occupied electrolyte: salt loading, swelling, and liquid inclusion may change the accessible opening and the carrier that crosses it. Channel crystallinity alone does not establish channel conduction. Recent MOF chemistry has explored ether functionality, controlled defects, crosslinking, cold operation, and computational screening [53,54,55,56,57,58].

Reported framework-based electrolytes occupy a range from dry single-ion solids to liquid-assisted composites. The mechanism must therefore be located in the operating membrane. If a carbonate or ionic liquid provides the dominant mobile phase, the framework may act as a selective host or a reinforcement. This remains a valuable design function, but it differs from ions moving through an intrinsically dry framework [37,59,60]. Lithium-rich porous aromatic frameworks and other tunable MOF quasi-solids require explicit accounting of their mobile phase [61,62].

### 3.4. Porous Ceramic Conductors and Infiltrated Scaffolds

A pore in a ceramic has at least two possible meanings for transport. It may remove part of the lattice available for conduction, or it may be deliberately filled with another electrolyte to create a composite path. A ceramic backbone can also redistribute stress and preserve electrode contact. Empty, infiltrated, and dense reference samples are needed to identify which effect explains an observed improvement [63,64]. Sodium thioantimonate, thiostannate, and NASICON structures provide contrasting continuous ceramic transport routes [65,66,67,68,69,70].

An image of a three-dimensional ceramic network establishes geometry only at the imaged scale. For a directional battery separator, through-plane continuity, bottleneck distribution, and the chemistry of the infiltrated phase matter more than total void fraction. Excess open volume can weaken fracture resistance and expose interfaces even as it improves infiltration. The useful porosity is therefore constrained by transport and mechanics together. Sodium chloride and iodide studies provide crystalline transport comparisons [71,72,73]; the mechanochemically synthesized Na–P–W–S system is a sulfide comparison, not a halide [74].

### 3.5. Taxonomic Distinction Between Accessible Pores and Structural Disorder

A glass can conduct ions through disordered local coordination without possessing connected, accessible pores. Atomic free volume is a molecular-scale descriptor and should not be substituted for the mesoscopic void network discussed for porous hosts and membranes. A glassy/crystalline sulfide surface micrograph does not itself measure atomic free volume. Glasses are useful controls because they test whether a reported transport benefit follows disorder and local bonding rather than added pore volume. Co-crystalline and glassy solid electrolytes offer disordered or nonporous comparison cases [75,76].

This control also changes the interpretation of a polymer composite. If a nonporous disordered filler yields a similar transport trend, pore-wall conduction is no longer the sole credible explanation. Pore accessibility must be demonstrated at the operational state before a glassy sample is grouped with open frameworks; otherwise, the comparison conflates short-range structural disorder with permeable architecture.

### 3.6. Taxonomic Overview of Layered and Interpenetrating Architectures

Hierarchical membranes allocate different tasks to different layers or networks. One region may conduct, another block metal penetration, and a third conform to the electrode. The strongest claim such an architecture can support depends on knowing which layer governs the measured response. A higher effective conductivity does not prove that all layers conduct rapidly; it may reflect improved contact at one limiting interface. Polymer infiltration of LFP–LATP cathodes [77], sodium–phosphate formation during chemical infiltration [78], and sodium rare-earth silicate electrolyte design [79] concern distinct structures and functions; they are not interchangeable demonstrations of one layered geometry.

For a Janus or graded design, direction matters. The thickness of each layer, local liquid uptake, orientation of pores, and through-plane versus in-plane transport should be reported separately. A reversible change under electrical bias must also be distinguished from redistribution of a retained liquid between layers. These controls allow hierarchy to be evaluated as functional allocation rather than a generic increase in complexity.

**Figure 3:**
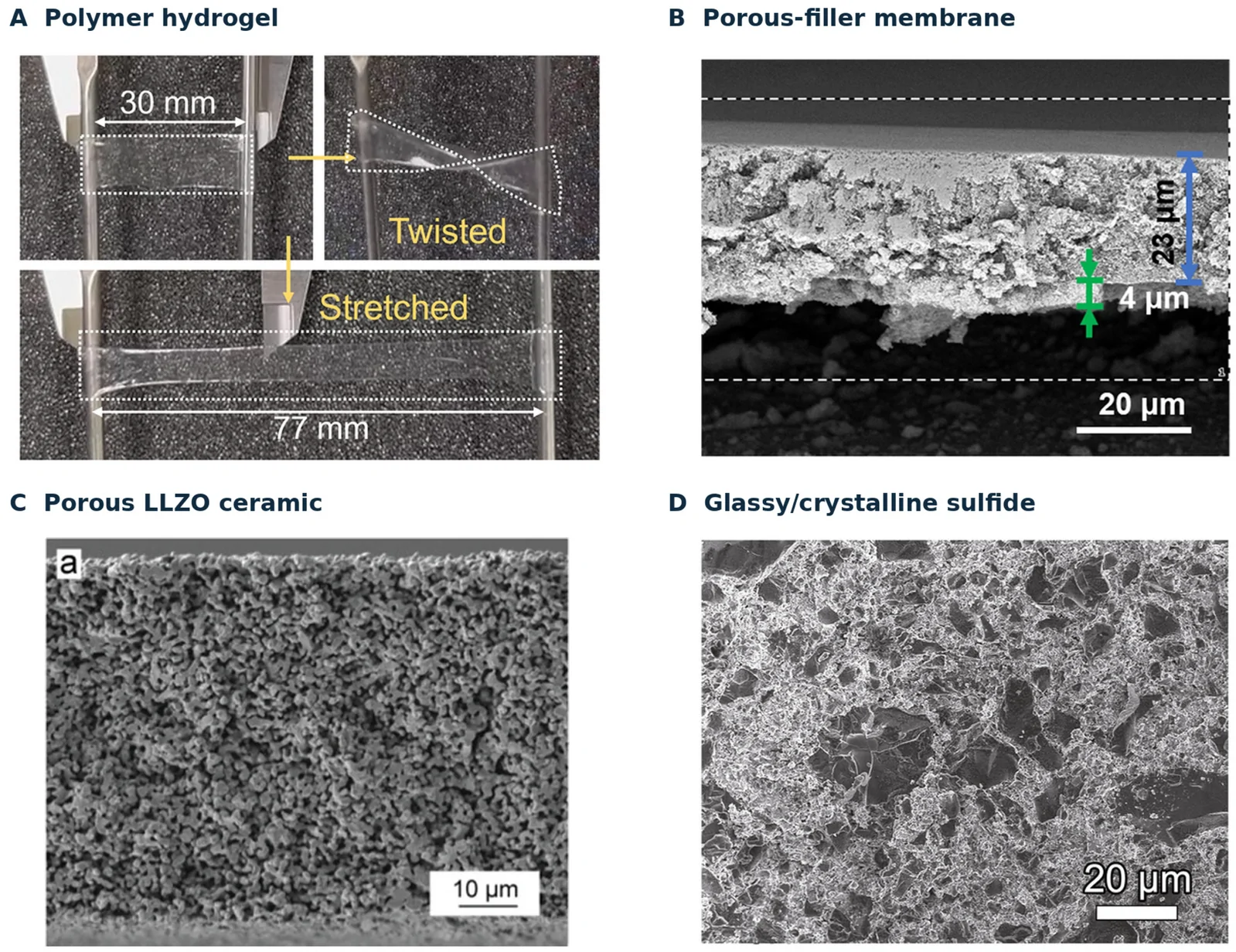
Published experimental electrolyte architectures. (**A**) Optical photographs of PAPTMA hydrogel during twisting and stretching: Lin et al. [81], Figure 1e in Ref. [81]. (**B**) Cross-sectional SEM of a Janus MOF/silica quasi-solid electrolyte membrane: Chen et al. [35], Figure 1g in Ref. [35]. (**C**) Cross-sectional SEM of a sintered porous LLZO membrane: Zhang et al. [84], Figure 3a in Ref. [84]. (**D**) Surface SEM of a cold-pressed glassy/crystalline sulfide composite with crystalline:glassy mass ratio C:G = 7: Su et al. [41], Figure 6e in Ref. [41]. Cropped, uniformly resized, and rearranged with Python 3.12.14 and Pillow 12.3.0; original scale bars and dimensional annotations retained. Sources (**A**): © The Author(s) 2025; (**B**,**D**): © The Author(s) 2024; (**C**): © 2024 The Authors, published by American Chemical Society. All panels are adapted under CC BY 4.0, https://creativecommons.org/licenses/by/4.0/ (accessed on 19 October 2026). In (**B**), the dashed horizontal lines delineate the membrane region, and the colored double-headed arrows mark the indicated layer thicknesses.

Figure 3 presents source-specific examples of hydrogel deformation, a porous-filler membrane, a porous ceramic, and a glassy/crystalline sulfide composite. These architectures require different transport tests [45,46,51,80,81,82,83]. The optical image and SEM sections establish specimen appearance and morphology; they do not identify a unique conducting phase. In particular, the LLZO panel shows a sintered porous ceramic, not a directly imaged polymer-infiltrated scaffold, and the sulfide surface image does not measure atomic free volume.

## 4. Porous Polymer, Fibrous, Gel, and Ionogel Electrolytes

Polymer-based porous electrolytes provide the clearest example of why physical state must precede materials classification. The same fibrous support can be evaluated dry, swollen with solvent, or hydrated. These are different ion-transport problems, even when microscopy shows the same skeletal geometry. We compare them by active mobile phase, mechanical role, and evidence for a pore-wall contribution [17,18,85]. Polymer-in-salt zinc and hydrated network studies are separate physical-state cases from dry Li polymer conductors [86].

### 4.1. Dry Porous Polymer Networks and Pore-Wall Conduction

In a nominally dry polymer network, a continuous solid phase has to connect the electrodes. Voids can lower tortuosity only if their walls or associated solid interphases support exchange; an empty insulating space cannot carry ionic current. Temperature-dependent relaxation, controlled humidity, and a dense polymer comparator help distinguish segmental transport from a channel-mediated contribution. Strongly complexed and chemically modified PEO formulations illustrate dry-polymer coordination controls [87,88,89,90]. Microdomain-interlocking polymer electrolytes provide another polymer-structure comparison [91] rather than an assumed liquid-filled gel.

Open pores can make a thin film compliant but can reduce load-bearing area and create high-field constrictions. A proposed dry-solid improvement should therefore be examined together with modulus, short-circuit behavior, and electrode contact. Conductivity gains caused by trace absorbed water are particularly easy to mistake for a new dry pathway unless conditioning and mass change are reported. Mechanical and interface changes in polymer–ceramic mixtures must be assessed together [92,93,94].

### 4.2. Liquid-Filled Porous Membranes, Gels, and Ionogels

Liquid-filled membranes and ionogels are best evaluated as coupled host-mobile-phase systems. The host controls uptake, spatial distribution, and leakage resistance; the contained phase often supplies much of the ionic mobility. Their strong cell performance is legitimate, but comparisons to solvent-free materials require explicit uptake, temperature, and salt-state information rather than the common label solid-state. Liquid-plasticized polymers and porous-matrix gels exemplify the need to report ion inventory and pore filling [95,96,97,98,99,100,101,102,103].

A higher filling fraction does not necessarily improve practical operation. It can weaken dimensional stability, increase chemical contact with electrodes, or change the apparent transference number through dilution. A useful experiment varies filling while tracking modulus, leakage or evaporation, and ion-specific transport. The optimum then reflects an engineering balance in a stated physical state, not a universal pore-volume target. Self-healing and adaptive gels are additional examples where mechanics and the mobile phase interact [104,105,106,107,108,109,110,111,112,113,114].

### 4.3. Electrospun Fibers and Hierarchical Polymer Supports

Electrospinning allows alignment and composition gradients across a membrane. The potential gain is directional organization and control of the contact layer. However, a micrograph of connected fibers cannot show whether ions cross inside fibers, along their surfaces, or through electrolytes held between them. Filling studies and direction-dependent impedance are needed to assign the path. PAN membrane studies [115,116] and an electrospun anode-free scaffold study [117] provide architecture-specific comparisons. Broader in situ polymerization reviews [118,119] and a functionalized carbon-nanotube channel study [120] address related processing and interface questions rather than all representing electrospun PAN membranes.

The large interface created by small fibers can also become a reaction site. Salt decomposition, moisture uptake, and binder or polymer relaxation can modify transport over cycling. Reporting the initial porous geometry without its post-operation condition may therefore miss the mechanism that determines cell lifetime. Where possible, the same region should be examined structurally and electrically before and after operation. Interfacial ligand chemistry and polymer phase structure provide independent controls for contact and stability [121,122,123,124].

For this material family, the causal question can be stated simply: what changes when the pore remains but the mobile phase, the wall chemistry, or the network connectivity is changed? Table 2 lists the controls needed to answer it. The purpose is to protect useful conclusions about gel and fiber architectures while avoiding an intrinsic-solid interpretation unsupported by the experiment.

## 5. Framework and Porous-Filler Electrolytes

Frameworks and porous fillers are attractive because internal surfaces can be designed independently of the surrounding polymer. Their mechanistic promise lies in selective interactions, but those interactions must be connected to a measurable mobile carrier and an accessible pathway. Table 3 and Table 4 therefore retain the reported physical state besides transport and cell metrics; a single conductivity ranking would obscure the distinction between a dry channel and a solvent-assisted host. Nanoscale confinement can couple diffusion and salt dissociation in polymer media [125,126].

### 5.1. Metal–Organic Frameworks

An MOF can expose polar sites within an ordered cavity and can alter salt distribution near a polymer interface. Whether those sites assist Li^+^ transport depends on channel access after mixing, occupied pore size, and the lifetime of cation and anion binding. Measuring a dry powder before membrane fabrication is not sufficient to establish these quantities. Conductivity and transference should be interpreted alongside solvent content and framework stability [127,128,129,130,131,132]. MOF–PEO composites and tunable framework-based quasi-solids illustrate the need to locate the conducting phase [52,62].

Defects change both the number and the heterogeneity of binding sites. Their presence might facilitate dissociation while also creating slow cation traps or reactive surfaces. A comparison with a nominally defect-free parent should document phase and moisture differences and should not attribute all changes to one calculated adsorption energy. Long-range transport requires exchange between sites and a route through the membrane, not only favorable local binding.

Related MOF examples include subnanometer channels and ultrathin composite films [133,134], conduction-mechanism analysis [135], a functional-design review [136], and crosslinked or MOF-808-containing composites [137,138]. Polydopamine-organized MOF networks [139], MOF-derived covalent crosslinking [140], and MOF/ionic-liquid polymer formulations [141] likewise belong to the MOF/composite comparison; they are not evidence for discrete organic cages or fixed-anion COFs.

MOFs with apparent BET areas above 5000 m^2^ g^−1^ are relevant to the design space. NU-1501-Al, for example, was reported at 7310 m^2^ g^−1^ while satisfying four BET consistency criteria [142]. That gas-storage study demonstrates ultrahigh gravimetric gas-accessible area, not battery–electrolyte performance. For an electrolyte host, this area must survive processing and chemical exposure, remain accessible after salt/polymer loading, and connect to through-plane transport. Higher area can increase useful interaction sites, but also the inventory of strongly binding or reactive sites; its net benefit requires matched-composition transport and stability controls. The moderate-area battery examples in Table 3 and Table 4 therefore do not define an upper area limit.

### 5.2. Covalent Organic Frameworks and Fixed-Anion Single-Ion Conductors

COFs permit channel-wall functionalization and, in some cases, tethered anions. This can reduce anion migration or organize local hopping environments, but the operating state is decisive. A swollen, solvent-activated COF and a dry fixed-anion framework can both be ion selective for different reasons. Their reported conductivity should be compared only with thickness, temperature, and retained liquid made explicit [36,38,143].

An effective channel assignment needs information about orientation, interlayer registry, and filling after salt incorporation. A favorable transference number does not prove every cation moves inside the framework: the polymer or confined liquid may carry much of the current. Species-sensitive spectroscopy with diffusion and directional transport would narrow the range of plausible pathways. Lithium- and sodium-conducting COF channel studies span distinct ion chemistries and physical states [45,144,145,146,147,148]. Soft-base anion-immobilized COFs are a separate fixed-anion example [149].

### 5.3. Porous Organic Cages, Porous Aromatic Frameworks, and Molecular Porous Solids

Discrete molecular cages [37], porous aromatic frameworks [150], and one-dimensional organic molecular porous solids [151] have different building units and connectivity. For each, a local cavity or adsorption site must exchange ions with a continuous path across the membrane. Empty-host area does not establish the spacing, accessibility, or conducting phase of the occupied film.

The CC3 quasi-solid example illustrates the distinction. Its proposed anion retention can be useful while the material still contains an organic mobile phase [37]. A stronger causal test would compare cavity accessibility and counterion dynamics under matched solvent loading. The relevant design variable is a selective, reversible exchange over the experimental time window rather than the strongest possible adsorption.

### 5.4. Quantitative Comparison and Physical-State Audit

Table 3 and Table 4 compare selected lithium-based framework electrolytes, not a cross-chemistry conductivity ranking. Their physical states, mobile-phase inventories, and test protocols must be retained when interpreting the values. Dry-powder gas adsorption is distinguished from gas adsorption on a composite and from ion access in an operating membrane. A computational MOF search does not establish conduction in a fabricated membrane [55].

The strongest mechanistic comparison combines pore access in the finished film, its contained liquid or water, species-sensitive coordination, and directional transport. The tables describe selected studies rather than a meta-analysis, and they do not convert proposed pore roles into demonstrated pathways.

Electrolyte regulation can alter both ion coordination and electrode reactions. In a water-in-LiCl gel, Zhu et al. used concentrated LiCl to regulate ion intercalation and suppress oxidation of MXene microelectrodes [152]. Chen et al. introduced ethylene glycol into an aqueous LiCl hybrid gel to obtain low-temperature operation of planar micro-supercapacitors [153]. These are examples of concentration- and solvent-dependent electrolyte/interfacial behavior in capacitive devices; their voltage and transport results are not intrinsic dry-solid conductivity benchmarks or evidence of battery–electrolyte memory.

For micropores (<2 nm), overlapping wall potentials cause pore filling rather than a separable monolayer followed by multilayer adsorption. We therefore use apparent BET surface area: a model-derived gas-adsorption descriptor, not an exact geometric or ion-accessible area. An unsuitable fitting interval can inflate the estimate; bias is not universally upward. The adsorptive, temperature, activation procedure, fitting interval, point count, and consistency checks are needed to judge reliability. A high regression coefficient alone is insufficient, and a fixed relative-pressure interval should not be imposed on every microporous isotherm [154,155].

The round-robin BETSI study found substantial variation when laboratories analyzed identical isotherms, supporting transparent reporting of raw adsorption data and fitting choices [154]. Probe identity also changes access and adsorption energetics [155]. For battery interpretation, these limitations are compounded by solvent occupation, polymer swelling, pore blocking, and phase connectivity. A decrease in apparent BET area after electrolyte incorporation can be consistent with pore occupation but does not itself quantify the ion-conducting volume or establish a transport pathway.

Table 4 now labels the sample associated with each apparent BET area and explains missing entries. NV means not verified from the accessible primary article or supporting information; it does not mean that the original authors never reported the quantity. NM means not mentioned in the accessible main text, with any unverified supporting information identified in the corresponding entry. Neither code is a zero value. Host-powder areas are not substituted for finished-electrolyte areas, and values are not estimated from a related MOF or a plotted curve without adequate numerical resolution.

Table 3 records thickness and the transference measurement description, where verified. EIS-derived conductivity, an operational polarization-based transference number, and a cation-specific conductivity are distinct observables. Reported polarization values should be compared only with compatible temperature, electrode, and reference-frame assumptions. Table 4 gives reported oxidation onsets rather than a universal thermodynamic stability window: working electrode, scan rate, onset criterion, and cell configuration matter. Current density without half-cycle areal charge does not establish an equivalent metal-cycling test.

Physical-state checks materially affect interpretation. The PHML fiber-network electrolyte contains an ionic liquid as well as PEO and LiTFSI [60]. The activated binary-MOF electrolyte retains a measured liquid-electrolyte fraction [34]. Neither is a solvent-free framework benchmark. For the Janus system, the dry-film thickness, EIS specimen thickness, and two-membrane symmetric-cell configuration are reported separately [35]. These distinctions prevent a nominally similar membrane label from concealing different conducting environments.

The supporting methods also separate measurements that must not be combined as though obtained under one condition. For polymer@TpPa-COOLi, the quoted conductivity is described as room-temperature, whereas the polarization-derived transference number was measured at 60 °C [156]. In-MOF/PVH contains residual DMF despite the original all-solid-state terminology [157]. Here the source classification is retained as provenance, while the comparison follows the measured mobile-phase inventory. Reported transference numbers are operational polarization results unless a different definition is specified.

**Table 3:** Selected lithium framework electrolytes: physical state, inventory, thickness, conductivity, and transference measurement. NV and NM are defined in Section 5.4; RT denotes the source’s room-temperature description without an assumed numerical temperature. M and m retain the source notation for molarity and molality; ambiguous source notation is identified. Water content is NV unless explicitly stated. Values are not normalized across physical states. All references to supplementary sections, figures, and tables refer to the Supporting Information of the original publications cited in the corresponding rows, not to supplementary material of the present review.

System	Audited State	Mobile Phase and Thickness	Reported Conductivity and T	Reported *t*_Li_^+^ and Method	Ref.
CC3 porous organic cage quasi-solid-state electrolyte (QSSE)	Quasi-solid; propylene carbonate (PC)-assisted	12 wt% PC; 11.4 wt% LiTFSI; activated in 1 M LiTFSI/PC; ~96 μm	1.25 × 10^−4^ S cm^−1^ at 25 °C (source label; SI EIS series: 303–363 K)	0.67 at 25 °C; Li||Li, 10 mV for 7200 s; before/after EIS, resistance-corrected polarization (Section S1 of the Supporting Information of Ref. [37])	[37]
F-COF donor–acceptor QSSE	Quasi-solid; PC-activated	1 M LiTFSI/PC activation; retained PC fraction NV; ~95 μm	6.7 × 10^−4^ S cm^−1^ at room temperature	0.83 at source RT; Li||Li DC polarization plus before/after EIS, resistance-corrected; bias NV (Section S5 of the Supporting Information of Ref. [38])	[38]
Li-COF-2@P75	Solvent-free single-ion composite (authors’ classification)	No added solvent or salt; polymerized PEGDA; ~30 μm composite (parent pellets ≥ 200 μm)	σLi^+^ = 8.9 × 10^−5^ S cm^−1^ at 25 °C (cation contribution)	0.95 at 298 K; Li||Li, 10 mV DC plus before/after EIS (SI Figure S20)	[36]
Polymer@TpPa-COOLi	Polymer/COF composite; residual-liquid fraction not quantified	Cellulose-supported in situ P(4CL–TMC)/COF; precursor: 25 mg LiTFSI + 5 mg LiI per 200 mg CL + 45 mg TMC; residual fraction and final thickness NV	1.1 × 10^−5^ S cm^−1^ at RT (exact T NV)	0.85 at 60 °C; Li||Li, 10 mV for 10,000 s; before/after EIS, resistance-corrected (Section S3 of the Supporting Information of Ref. [156])	[156]
In-MOF/PVH	Solvent-containing polymer composite	14.3 wt% residual DMF by TG; recipe PVH/LiTFSI/In-MOF = 0.60/0.60/0.42 g; final thickness NV	1.23 × 10^−3^ S cm^−1^ at 25 °C	0.49 at 25 °C; Li||Li, 10 mV DC; before/after EIS (5 mV AC), resistance-corrected (Section S1 and Figure S21 of the Supporting Information of Ref. [157])	[157]
Janus MOFLi/MSLi	Quasi-solid; PC-assisted Janus membrane	1 M LiTFSI/PC; 23/39 wt% uptake in separate components; dry film ~27 μm; EIS membrane 29 μm	2.2 × 10^−4^ S cm^−1^ at room temperature	0.71; Li||Li current–time/EIS (SI Figure S8); bias NV	[35]
ZIF-67–LA–PAM	Liquid-soaked gel/quasi-solid composite	Source: “1 m” LiPF_6_ in EC/DMC/EMC (1:1:1 *v*/*v*/*v*); retained fraction NV; ~48.9 μm dry membrane	3.84 × 10^−3^ S cm^−1^ at 30 °C	0.627 at 30 °C; chronoamperometry plus EIS, resistance-corrected polarization; bias NV	[158]
Binary-MOF hierarchical film	Semi-solid; PC-assisted	Activated composite retains 11.41 wt% LE (source mass convention); LiTFSI/PC “1 m”/“1 M” notation inconsistent; ~60 μm	2.04 × 10^−4^ S cm^−1^ (temperature of this value not explicitly stated)	Value for combined film NV; method: Li||Li, 10 mV DC plus before/after EIS, Bruce equation	[34]
MOF-fiber/PEO/LiTFSI/IL (PHML)	Ionic-liquid-containing polymer composite	[EMIM][TFSI] present; retained IL fraction and salt loading NV; 56.92 μm	1.36 × 10^−4^ S cm^−1^ at 30 °C (abstract); main text gives 1.34 × 10^−4^	0.49; Li symmetric-cell test; full polarization protocol NV	[60]
ZIF-8/PP ionic-liquid membrane	Ionogel-like solid membrane	Li-[EMIM][TFSI]; retained fraction, salt concentration and thickness NV	2.09 × 10^−4^ S cm^−1^ at 25 °C	0.45 (publisher abstract); detailed polarization protocol NV; primary full text/SI not accessible in this audit	[159]

**Table 4:** Selected lithium framework electrolytes: sample-specific apparent BET area, reported oxidation onset, metal-cycling conditions, and proposed pore function. Areas are in m^2^ g^−1^. Powder and dry-composite measurements do not establish operando ion access. Missing protocol details are marked NV or NM as defined in Section 5.4. All references to supplementary sections, figures, and tables refer to the Supporting Information of the original publications cited in the corresponding rows, not to supplementary material of the present review.

System	Sample and Apparent BET Area	Reported Oxidation Onset	Metal/CCD Protocol	Proposed Pore Role	Ref.
CC3 porous organic cage QSSE	CC3 powder: 426; prepared QSSE: 7; gas-adsorption state, not operando access	4.7 V, reported LSV; Li|QSSE|SS, 1.0 mV s^−1^; numerical onset criterion NV	CCD 1.60 mA cm^−2^; Li||Li > 2000 h at 0.1 mA cm^−2^, 0.1 mAh cm^−2^	Cavity-induced TFSI^−^ trapping and confined-PC-assisted Li^+^ hopping	[37]
F-COF donor–acceptor QSSE	F-COF powder: 147; pore size ~1.8 nm; finished-film area NV	>5 V, reported LSV; scan rate and onset criterion NV	CCD 5 mA cm^−2^; Li||Li > 1000 h at 0.1 mA cm^−2^, 0.1 mAh cm^−2^	Donor–acceptor electron density regulates anion binding and Li^+^ migration	[38]
Li-COF-2@P75	Li-COF-2 precursor: 95; pores ~1.2 nm, volume 0.21 cm^3^ g^−1^; composite area NV	LSV: Li|electrolyte|SUS, 0.1 mV s^−1^; numerical onset NV	Li||Li > 500 h at 0.05 mA cm^−2^ and 0.25 mAh cm^−2^ per half-cycle, 25 °C	Weak ion–dipole interactions lower Li^+^ hopping barrier in fixed-anion channels	[36]
Polymer@TpPa-COOLi	TpPa-COOLi powder: 75.96 (N_2_, 77 K); pores 1.37–1.70 nm; polymer composites show a decrease, numerical areas NV	Li|electrolyte|SS LSV, 0.1 mV s^−1^ at 60 °C; formulation-specific numerical onset NV	CCD 0.50 mA cm^−2^; source also tests Li||Li at 0.1 mA cm^−2^, 60 °C; half-cycle charge NV	COF–polymer junctions and fixed COO^−^ groups create selective pathways	[156]
In-MOF/PVH	Numerical apparent BET area not located in inspected main text/SI; operating-membrane access not established	4.90 V, source-reported low-current limit; SS|electrolyte|Li, 0.1 mV s^−1^ at 25 °C; current threshold NV	CCD 1.7 mA cm^−2^; Li||Li 5550 h at 0.2 mA cm^−2^, 25 °C; half-cycle charge NV	MOF regulates polymer coordination/interfaces; DMF materially contributes to transport	[157]
Janus MOFLi/MSLi	MOF/MS powders: 1130/725; dry MOF/MS–PVDF-HFP composites: 878/374 (N_2_, 77 K; SI Table S1); Janus electrolyte-filled area NV	Components MOFLi/MSLi: 4.25/4.6 V; Li|electrolyte|SS, 1 mV s^−1^; Janus onset NV	CCD 2.1 mA cm^−2^; symmetric cells use two Janus membranes, MOF sides toward Li; distinct from one-film EIS geometry	Asymmetric pore scales separate fast ion supply and selective interfacial transport	[35]
ZIF-67–LA–PAM	Dry composite area increases sixfold vs. LA–PAM (source SI Figures S9–S11); absolute area NV; film porosity ~88%	5.2 V, reported LSV; Li|electrolyte|SS, 5 mV s^−1^ at 30 °C; onset criterion NV	Reported Li||Li cycling at 40–100 mA cm^−2^, 30 °C; half-cycle charge NV; not ranked against matched-charge tests	MOF increases accessible pores, uptake and ion-regulating sites	[158]
Binary-MOF hierarchical film	Component powders: HKUST-1 215–234; ZIF-8/Cu–Zn MOF ~1690; activated binary-film area NV	5.25 V vs. Li/Li^+^; Li|electrolyte|SS LSV (3–6 V); scan rate NV	Li||Li > 1000 h at 0.5 mA cm^−2^, 0.5 mAh cm^−2^ per half-cycle, RT	Large-pore field transport plus micropore hopping creates dual pathways	[34]
MOF-fiber/PEO/LiTFSI/IL (PHML)	Precursor pore size ~1.74 nm; absolute area NV owing to source unit inconsistency; finished-film area NV	5.32 V, reported LSV; electrode, scan rate and onset criterion NV	Li||Li 3000 h; half-cycle charge and cycling T NV	MOF network, polymer and ionic liquid jointly affect transport; intrinsic framework conduction not isolated	[60]
ZIF-8/PP ionic-liquid membrane	Absolute area and finished-film gas-accessible pores NV; primary full text not accessible in this audit	NV	Li||Li 550 h at 0.1 mA cm^−2^; half-cycle charge and T NV	Porous host retains ionic liquid and regulates transport while remaining freestanding	[159]

## 6. Porous Ceramic and Three-Dimensional Scaffold Electrolytes

The role of a ceramic scaffold depends on which phase connects the two electrodes after processing. A continuous garnet skeleton, isolated ceramic particles within polymer, and a porous ceramic infiltrated with gel may have similar apparent composition but different routes and failure modes. The material should be evaluated as a coupled transport–mechanical structure rather than as a pore fraction alone.

### 6.1. Porous Garnet and Oxide Membranes

A porous LLZO membrane can admit a compliant phase and maintain contact with lithium, while its dense ceramic fraction offers a selective lattice route. The relative contributions depend on backbone connectivity, infiltrant uptake, thickness, and grain-boundary chemistry. Without these measurements, an impedance reduction can be assigned to improved contact just as plausibly as to a faster bulk path. Printed porous LLZTO and rapidly sintered garnet scaffolds provide geometry-specific ceramic test cases [46,160].

Wettability measurements on porous anodic alumina provide a methodological comparison: changing synthesis and pore-widening conditions alters membrane morphology and liquid contact angle [161]. This shows why pore dimensions should be considered together with wetting when evaluating infiltration. Contact angle alone does not establish internal saturation, connected electrolyte access, or a conducting pathway, and this non-battery membrane study does not validate Li-garnet transport.

Removing ceramic to create voids reduces the nominal cross-section available for ceramic conduction. The gain from infiltration or interface conformity must compensate for that loss. Measurements on the empty scaffold, infiltrated scaffold, and dense control can establish whether the pore is an active transport element, a processing aid, or a mechanical compromise. Cell cycling under reported pressure provides the application context.

### 6.2. Vertically Aligned and Bicontinuous Scaffolds

Aligned ceramic fibers or channels can reduce through-plane detours; yet, a two-dimensional aligned image cannot prove that the paths are continuous across membrane thickness. A directional measurement together with three-dimensional structural evidence is needed for that claim. Polymer surrounding the aligned phase may contribute in parallel or provide the dominant contact improvement. Ferroelectric ceramic nanofibers and polymer composites illustrate how alignment, interfaces, and electrostatics may covary [162].

In a porous electrode, the same structural principle can improve infiltration: an electrolyte must reach a large internal reaction area without leaving disconnected dead ends. That architecture must be judged against active-material loading, electrolyte mass, and impedance growth. The most useful comparison keeps these quantities fixed while varying scaffold continuity rather than ranking a highly infiltrated electrode against a dense one under different loading. Polymer infiltration of composite cathodes is an example of an access benefit distinct from bulk electrolyte mobility [77].

### 6.3. Mechanical, Interfacial, and Field-Mediated Limits

Pores can focus stress and electrical field at curved interfaces. Improved wetting during assembly may coexist with greater risk of cracking during cycling. Fracture imaging and impedance evolution should therefore be read together. A long symmetric-cell test is more interpretable when current density, areal capacity, pressure, and cumulative charge are included [23,24,25,26,27].

Polar ceramic inclusions may alter local electrostatic fields, but a measured conductivity increase cannot uniquely identify a field-mediated mechanism. Changes in polymer crystallinity, salt association, and water content provide alternatives. A depolarized or chemically matched inclusion can test whether orientation or polarization has a causal role, alongside structural and ion-specific measurements.

The ceramic cases show why a high-performing hybrid should not be forced into one conduction label. The scaffold may carry ions, support an infiltrant, redistribute stress, or stabilize an interface; several contributions can coexist. The useful conclusion is a measured allocation of functions with uncertainties, not an assumption that greater porosity is always advantageous. Table 5 summarizes the attribution questions and measurements for these ceramic-rich and disordered controls.

## 7. Glass, Glass–Ceramic, and Disordered Controls

Glasses and disordered ion conductors provide an important control for claims of pore-mediated transport because atomic free volume, topological disorder, and phase boundaries can create low-barrier pathways without forming accessible pores. Treating these features as equivalent to meso- or microporosity can therefore misassign the physical origin of conductivity.

### 7.1. Atomic Free Volume Is Not Automatically Porosity

Glassy sulfides, oxyhalides, halides, and related amorphous conductors contain distributions of bond lengths, coordination numbers, and interstitial volumes. In lithium and sodium electrolytes, such disorder can create connected low-barrier configurations without gas-accessible voids; zinc glasses and amorphous halides remain comparatively sparse. Atomic free volume and accessible porosity must therefore be treated as distinct descriptors because they imply different transport, infiltration, and mechanical functions [41,163,164,165]. Amorphous sodium halides and simulated NaPSO glass provide additional non-MOF disorder comparisons [42,166,167,168]. Iodide, chloride, and substituted halide studies furnish crystalline transport controls rather than evidence of accessible porosity [169,170,171,172,173,174,175,176,177].

Lithium–phosphate glasses illustrate a composition-dependent transport control. Ouachouo et al. found that replacing Li_2_O with ZrO_2_ changes the network and reduces the reported ionic conductivity [178]. Because lithium inventory and network chemistry change together, this is not an isolated porosity intervention. Their borophosphate study further reports composition-dependent ionic and electronic contributions [179]. These examples reinforce the need to resolve charge-carrier identity and structural chemistry before assigning a conductivity change to accessible pores; neither study establishes a programmable retained ionic state.

Atomistic simulation and total-scattering studies show that disorder can enhance transport by increasing the population or connectivity of favorable local configurations, but excessive disorder can also create deep local minima or disconnected favorable sites. The relevant quantity is consequently the connectivity of low-barrier configurations rather than the total amorphous fraction. Sodium argyrodite screening and halide substitution studies illustrate why modeled pathways need material-level checks [173,180].

### 7.2. Glass–Ceramic Phase Connectivity

Glass–ceramic electrolytes contain crystalline and glassy domains whose relative contributions depend on composition, phase fraction, crystallite size, density, connectivity, and interface chemistry. Crystallization can increase conductivity by forming a fast phase, decrease it by consuming a favorable glass composition, or improve mechanics while increasing grain-boundary resistance. Phase boundaries should not be counted as pores unless a true void is directly observed [181,182,183]. Grain-boundary and phase-connectivity interpretation can be tested against varied sodium conductor families [68,70,184].

Modified lithium thiophosphate, thioborate, argyrodite, and related systems therefore function in this review as mechanistic controls: they test whether a conductivity increase attributed to ‘porosity’ could instead arise from disorder, crystallization, or connected low-barrier domains. Solution processing of sulfide conductors changes synthesis and interphase variables alongside phase structure [185].

### 7.3. MOF Glasses and Vitrified Frameworks

MOF glasses occupy an intermediate category because a porous crystalline precursor can lose long-range channels during vitrification while retaining local cavities, free volume, or chemically heterogeneous coordination environments. Some MOF–glass electrolytes also contain confined solvent or salt. Claims of pore-mediated conduction therefore require evidence that accessible voids survive vitrification, together with local structural characterization and a mass balance of any mobile phase [83,186,187]. These MOF-derived systems are distinguished from the inorganic glasses and amorphous halides in Section 7.1.

A framework glass can provide a grain-boundary-free matrix with chemically programmable local environments, but the terms ‘glassy,’ ‘porous,’ and ‘quasi-solid’ describe different physical attributes and should not be used interchangeably. Matched comparisons among crystalline framework, densified glass, and liquid-containing glass states are particularly useful for identifying which attribute controls transport.

## 8. Hierarchical, Janus, Gradient, and Bicontinuous Architectures

Hierarchy is valuable when one architecture cannot simultaneously maximize mobility, contact, and blocking ability. A graded membrane may place a conformal region against metal and a denser layer against an electrode prone to penetration. The design claim should name which function belongs to which region and provide a measurement resolving it. Architecture-level examples include asymmetric porous polymer membranes and sodium rare-earth silicate electrolyte design [49,79].

An effective impedance combines all layers and contacts. If a thin interlayer removes a dominant contact resistance, the total response can improve even though the intrinsic conductivity of the bulk remains unchanged. Layer-resolved thickness, directional measurements, and comparisons with each constituent are necessary before assigning the gain to a novel connected pore network.

Bicontinuity can couple a rigid ceramic support with a compliant polymer or gel path. Both phases may percolate while only one carries most ions under a given physical state. Three-dimensional network measures and operating-state uptake are needed to identify the conducting route. The same architecture may change function as temperature or solvent content changes. Ceramic scaffold fabrication [46,160] and composite–cathode infiltration [77] provide separate architectural comparisons; neither alone establishes that both phases carry a substantial through-plane ionic current.

These designs are therefore judged by controlled functional separation rather than by geometric complexity. A useful comparison reports the membrane orientation, electrode-facing chemistry, local mobile-phase inventory, and failure location after cycling. Such information links published electrolyte morphologies to the interface observations discussed in Section 9.1 without presuming a universal optimum.

## 9. Electrode–Electrolyte Interfaces and Li/Na/Zn Applications

The electrode interface determines whether a pore-bearing membrane retains its benefit in a full cell. Contact, mobile-phase inventory, interphase chemistry, and local current distribution may change together. Evidence from Li, Na, and Zn cells should therefore be read with the electrode reaction and test conditions rather than treating longer cycling as direct proof of an internal pore-conduction pathway [23,28,188].

### 9.1. Interfacial Ion Flux, Voids, Dendrites, and Filaments

Plating and stripping depend on transport normal to the interface, the evolving reaction layer, and mechanical contact. A conformal membrane can reduce initial gaps, but its pore mouths can also become sites of localized deposition. Interpretation needs current density, areal charge, pressure, and cross-sectional evidence after a defined number of cycles [23,24,25,26,27]. Critical-current studies and second-phase NASICON modifications illustrate coupled mechanical and electrochemical limits [189,190,191].

**Figure 4:**
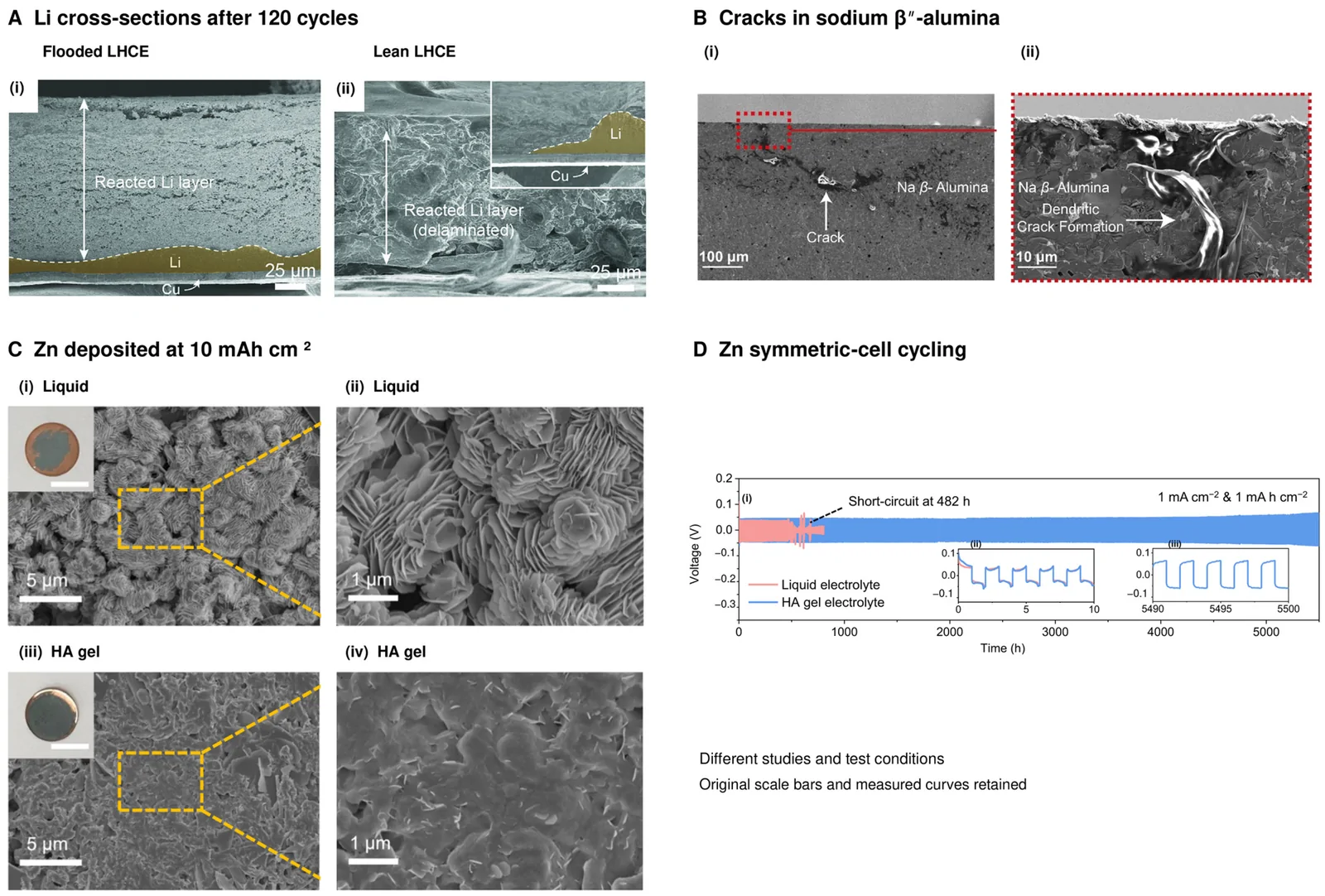
Source-specific electrode–interface observations. (**A**) Cross-sectional SEM images of Li anodes in Li||NMC622 cells with flooded (**A(i)**) and lean (**A(ii)**) LHCE after 120 cycles: Figure 5c,f in Ref. [23]. (**B**) SEM images of cracking in sodium β″-alumina at lower (**B(i**)) and higher (**B(ii)**) magnification: Figure 2c,d in Ref. [188]. The red outlined region in (**B(i)**) corresponds to the higher-magnification view in (**B(ii)**). (**C**) Zn deposited on Cu at 1 mA cm^−2^ to 10 mAh cm^−2^ in liquid electrolyte (**C(i,ii)**) and HA gel electrolyte (**C(iii,iv)**): right-hand low/high-magnification pairs of Figure 3d,e in Ref. [28]. SEM scale bars are 5 μm and 1 μm; optical-inset scale bars are 1 cm. Yellow dashed outlines identify the magnified regions. (**D**) Zn||Zn cycling at 1 mA cm^−2^ and 1 mAh cm^−2^: Figure 5a in Ref. [28]; (**D(i)**) is the main trace, and (**D(ii,iii)**) are the early- and late-time insets. Red and blue traces identify liquid and HA gel electrolytes, respectively. Adapted by cropping, proportional resizing, relabeling, and rearrangement using Python 3.12.14 and Pillow 12.3.0; scale bars and measured curves are retained, and minus signs in axis annotations have been retypeset. Copyright © 2022 the authors (**A**); © 2020 the authors (**B**); © 2023 the authors (**C**,**D**). All sources are adapted under CC BY 4.0, https://creativecommons.org/licenses/by/4.0/ (accessed on 19 October 2026). These observations have different test conditions and are not a matched performance comparison.

The failure modes differ across chemistries and methods. In Li cells, a porous reaction layer can accumulate inactive metal when electrolyte becomes locally depleted [23]. Sodium penetration through β″-alumina has been resolved with sodium-sensitive imaging and associated with crack growth [188]. In hydrated Zn cells, surface roughness also reflects corrosion and hydrogen evolution [28]. Figure 4 illustrates these separate observations; the juxtaposition is conceptual and does not make their test conditions equivalent.

Figure 4 juxtaposes source-specific Li-interface cross-sections, cracking in sodium β″-alumina, and Zn deposition/cycling observations. The Li comparison concerns liquid localized high-concentration electrolyte (LHCE), the Na images concern a solid ceramic, and the Zn comparison concerns aqueous liquid and hyaluronic-acid (HA) gel electrolytes. These examples illustrate distinct interfacial processes; they are not matched tests of electrolyte performance or evidence of ionic memory.

### 9.2. Lithium–Metal and Anode-Free Batteries

Lithium–metal studies should distinguish ion delivery from surface chemistry and lithium inventory. Increased scaffold area can improve wetting while also increasing electrolyte consumption. Match cathode loading, lithium thickness, electrolyte quantity, pressure, and cumulative plated charge before attributing a stability gain to pore regulation [23,26]. An electrospun anode-free scaffold [117] and an artificial interphase for anode-free lithium cells [192] address finite-inventory lithium configurations. Electrochemical–mechanical and interphase-growth measurements provide broader tools for resolving contact and reaction contributions [193,194].

In anode-free lithium cells, plated lithium originates in the cathode. First-cycle efficiency, irreversible inventory loss, scaffold mass, and absorbed electrolyte therefore belong in the cell-level balance [117,192]. A long symmetric-cell trace does not resolve that finite-inventory question. Sodium interfacial and sodiation studies are discussed separately in Section 9.3.

### 9.3. Sodium–Metal and Sodium-Ion Batteries

Sodium transport should be evaluated with Na-specific coordination, pore access, and the phase that actually spans the membrane. A glass–ceramic grain boundary, a liquid-filled polymer void, and an intrinsic framework channel are not interchangeable routes. Interfacial contact can determine the observed impedance even when the bulk conductor is unchanged [195,196,197,198,199]. Na-specific polymer examples include polyisoprene, NASICON-compatible, and localized-concentration systems [200,201,202,203,204,205].

Na-specific examples include edge-growth control in NaSICON [206], ferroelectric interface modification [207], β-alumina contact engineering [208], NASICON interface dynamics [209], and Na–K alloy anodes [210]. Sulfide/anode degradation [211] and sodium–phosphate sodiation imaging [212] concern different electrode reactions and are not anode-free lithium evidence. The low-temperature sodium-ion hybrid capacitor in [213] is an adjacent-device comparison, not a solid-state battery validation.

A recent fluorinated-MOF/PEO study illustrates an Na-specific coordination intervention: the selected formulation uses NaTFSI with EO/Na = 16 and reports 1.01 mS cm^−1^ at 60 °C [214]. The temperature and polymer coordination environment are essential to interpreting that value; it is not a room-temperature comparator for Table 3. Its combined spectroscopy and simulation support the proposed coordination change without establishing a universal pore-size or surface-area optimum.

For Na metal, pressure, fracture, and deposition location deserve explicit measurements. The β″-alumina study associated dendritic sodium with cracks, while its imaging method resolved features that ordinary bulk impedance cannot locate [188]. Comparisons with Li require Na-specific current, temperature, inventory, and phase-state controls rather than a transferred pore-size optimum [215,216,217]. Na–Sn interface modification and composite cathode contacts further show that both electrode boundaries matter [218,219].

### 9.4. Zinc–Metal, Aqueous, and Quasi-Solid Zinc Batteries

Hydrated Zn systems couple Zn^2+^ coordination to water activity, pH, hydrogen evolution, corrosion, and precipitation. A gel can distribute current and change deposition morphology while water remains the dominant mobile phase. Wall charge and coordination may be useful design variables, but their contribution should be isolated from gel uptake and bulk Zn salt concentration [28,220,221,222,223,224,225,226]. Zn coordination, hydrogen evolution, hydrophobic interfaces, and water management are independent variables in hydrated cells [227,228,229,230,231]. Aqueous anti-freezing-electrolyte development [232] and a zincophilic polyanionic hydrogel interfacial layer [233] are Zn-specific examples; the latter is a protective layer rather than automatically a bulk electrolyte.

The HA gel example illustrates how a controlled electrolyte chemistry can alter Zn surface morphology [28]; that observation alone does not establish conduction along pore walls. Quantified roughness, Coulombic efficiency, corrosion or gas measurements, areal charge, electrolyte amount, and repeated cells provide a more defensible link from membrane design to Zn cycling. Hydrogel, zwitterionic, biomacromolecular, and crosslinked formulations offer Zn-specific battery comparisons [234,235,236,237,238,239,240,241,242,243]. The zwitterionic systems in [244,245] are zinc-ion hybrid capacitors whose device-level performance is not a battery cycling benchmark.

Recent quasi-solid Zn evidence also links channel chemistry to interfacial reactions. The sodium polyacrylate/lithium magnesium silicate/cellulose nanofiber (PAAS/LMS/CNF) electrolyte, prepared by immersion in 2 M ZnSO_4_, was examined using coordination-sensitive measurements and characterization of zinc hydroxysulfate by-products [246]. The same study also attributes improved deposition to an in situ Zn–Mg–Si interphase [246]; the result therefore does not isolate channel geometry from electrode chemistry. This motivates reporting local pH, water state, and precipitation alongside ion transport. Water mass fraction and thermodynamic water activity are different quantities; neither conductivity nor an O–H spectral change alone quantifies water activity. Conductivity can include anions and proton-related species, so a Zn^2+^ pathway requires species-sensitive evidence.

A 2026 aqueous Zn study further reports that trace Ti_3_C_2_Tₓ MXene in a low-concentration zinc acetate electrolyte jointly modifies solvation and the electrode interfaces [247]. This supports considering electrolyte composition and interfacial reconfiguration together. It does not isolate a bulk pore-architecture effect: the additive acts at both electrodes, and the system remains aqueous. Such coupled effects must be separated before attributing improved cycling or a delayed response to an intrinsic retained ionic state.

### 9.5. Cross-Chemistry Application Constraints

Table 6 separates common measurement requirements from chemistry-specific interpretation. The comparison concerns selected nonaqueous Li/Na systems and hydrated Zn systems; it is not a claim that every Li/Na electrolyte is nonaqueous or every Zn electrolyte is aqueous. Geometry descriptors may be shared, but numerical binding energies, carrier sizes, association equilibria, interfacial products, and retention thresholds must be determined for each chemistry [34,35,36,37,38,156,157,214,246].

Use conditions determine the appropriate architecture. Thick electrodes prioritize infiltration and directional access; high-rate cells require a known mobile-ion inventory and manageable polarization; flexible cells must retain contact during deformation. These demands interact with voltage, temperature, electrolyte quantity, and pressure. Table 7 lists discriminating cell-level checks rather than ranking Li, Na, and Zn by one metric [248,249,250,251,252]. Flexible zinc devices and mechanically robust gels demonstrate application-specific requirements [253,254,255,256].

## 10. Cross-Cutting Mechanistic Evidence

The comparisons in Section 4, Section 5, Section 6, Section 7, Section 8 and Section 9 leave four possible origins of a transport change: carrier availability, local mobility, architectural access, and electrode contact. Section 10.1, Section 10.2, Section 10.3, Section 10.4 and Section 10.5 distinguish these mechanisms; Section 10.6 adds the temporal evidence needed for a post-field state.

### 10.1. Minimal Transport Basis: Electrochemical Potential and Flux

A coarse-grained description starts from the molar electrochemical potential of species *i*. To make the assumed contributions explicit, we write the following phenomenological decomposition:

(1) $$\tilde{\mu_{i}}=\mu_{i}^{\circ }+RT\;lna_{i}+z_{i}F\phi +\Omega_{i}\sigma_{h}+{\Delta G}_{i,wall}+{\Delta G}_{i,conf}$$

In Equation (1), $\tilde{\mu_{i}}$ is the molar electrochemical potential and *μ_i_*^∘^ is the chemical reference term (J mol^−1^); *R* is the gas constant (J mol^−1^ K^−1^); *T* is absolute temperature (K); *a_i_* is dimensionless ionic activity under a stated standard-state convention; *z_i_* is the signed charge number; *F* is the Faraday constant (C mol^−1^); and *ϕ* is electric potential (V). The stress coefficient *Ω_i_* has units m^3^ mol^−1^ and *σ*_h_ is hydrostatic stress (Pa), with compression taken positive here. The terms Δ*G_i_*_,wall_ and Δ*G_i_*_,conf_ are effective molar contributions from pore-wall interactions and confinement (J mol^−1^) [257,258]. This separation is bookkeeping, not a claim that these effects are independent: effects already absorbed into activity or the reference term must not be added again. At uniform temperature and without convection, a diagonal-mobility approximation gives

(2) $$J_{i}=-\frac{D_{i}c_{i}}{RT}\nabla \tilde{\mu_{i}}$$

In Equation (2), *J_i_* is molar flux relative to the specified stationary host or solvent reference frame (mol m^−2^ s^−1^), *D_i_* is the positive kinetic diffusivity entering the assumed mobility relation (m^2^ s^−1^), *c_i_* is molar concentration (mol m^−3^), and ∇ denotes the spatial gradient. Inserting Equation (1) retains the activity, electrical, stress, pore-wall, and confinement gradients. For an ideal dilute phase with constant reference and excess-energy terms, the activity contribution reduces to the usual Fickian term. This approximation neglects off-diagonal species correlations and does not replace concentrated-solution or Onsager transport analysis. The kinetic *D_i_* must not also include an activity-gradient correction that is explicitly counted in the driving force; convection requires an additional advective term.

The electrical potential is additionally coupled to local charge density. In a charged or selectively adsorbing pore,

(3) $$-\nabla \cdot (\varepsilon \nabla \phi )=F\sum_{i}z_{i}c_{i}+\rho_{f}+\rho_{trap}$$

In Equation (3), *ε* is absolute permittivity (F m^−1^), *ρ*_f_ is fixed structural charge density, and *ρ*_trap_ is separately represented trapped or slowly relaxing ionic charge density (both C m^−3^). The sum includes only ionic populations counted in the mobile concentrations *c_i_*; charges included in *ρ*_trap_ must be excluded from that sum. This prevents double-counting the same ions. *F*, *z_i_*, and *ϕ* retain their definitions above. The continuum Poisson equation permits local space charge; local electroneutrality is a further approximation, not an automatic pore-wall condition. Molecular-scale confinement may require a noncontinuum description.

### 10.2. Connectivity, Tortuosity, and Effective Transport

Local conductivity and macroscopic geometry should be separated only under a stated homogenization model. For a single dominant conducting phase, we use an aggregate directional architecture factor:

(4) $$\sigma_{eff}\approx \sigma_{local}G_{arch}$$

In Equation (4), *σ*_eff_ is macroscopic effective ionic conductivity and *σ*_local_ is the conductivity of a matched local conducting phase (both *S* m^−1^). The dimensionless *G*_arch_ is one aggregate architecture factor for the specified transport direction and physical state. It incorporates the joint effects of accessible conducting volume, tortuosity, constriction, connectivity, and orientation. These geometric effects are generally correlated and are not multiplied as independently measured corrections. *G*_arch_ must be calculated from an explicit transport model or interpreted as a ratio of matched conductivities; it cannot itself prove a pore mechanism. The relation is not a general mixing law for multiple parallel conducting phases, and changes in local chemistry or interfacial conductance must not be silently assigned to geometry [17,63,257,259,260,261,262,263]. These material-design studies provide context; they do not independently validate this aggregate factor or a universal geometry law.

Architecture determines which conducting phase must be connected: polymer, pore surface, or confined liquid in polymer membranes; accessible internal channels and interfaces in framework composites; and the ceramic skeleton in porous conductors. Ceramic voids remove conducting cross-section unless reduced thickness, infiltration, contact, or alignment compensates [39,40,64,82,84,264,265,266,267,268,269,270].

Total conductivity alone does not establish useful transport by the electrochemically active cation. Where the transference definition and measurement protocol are compatible, an operational screening quantity can be defined as

(5) $$\sigma_{M,screen}\equiv \sigma_{tot}t_{M}$$

In Equation (5), M is the target cation (Li^+^, Na^+^, or Zn^2+^), *σ*_M,screen_ is an operational target-cation screening quantity (*S* m^−1^), *σ*_tot_ is measured total ionic conductivity (*S* m^−1^), and *t*_M_ is its reported transference number under a stated method and reference frame. A steady-state apparent transference number, a tracer-derived estimate, and a concentrated-solution transference number must not be interchanged without justification. The product is not an independently measured partial conductivity or a replacement for a full multicomponent transport analysis. It supports comparison only when the definitions and physical-state conditions are compatible.

### 10.3. Confinement, Salt Speciation, and the Effective Carrier State

The species approaching a bottleneck may be a solvated or polymer-coordinated ion, an ion pair, or a larger complex. Confinement therefore couples geometry to coordination and dielectric response, as illustrated by nanoconfined ion pairing [44].

For a generic salt with one target cation of positive valence z and z monovalent counterions, the formal reaction is MA_z ⇌ Mᶻ^+^ + zA^−^. Under specified pore-phase standard states, the dimensionless dissociation descriptor is

(6) $$K_{d,pore}=\frac{a_{M}{a_{A}}^{z}}{a_{{MA}_{z}}}$$

In Equation (6), M denotes the target cation, A a monovalent counterion, and *z* the positive cation valence (1 for Li^+^/Na^+^ and 2 for Zn^2+^). *K*_d,pore_ is the dissociation equilibrium constant for the stated reaction and pore-phase convention; *a*_M_, *a*_A_, and *a*_MA*z*_ are dimensionless activities of the cation, counterion, and undissociated species, respectively. In general, *a_j_* denotes the activity of species *j*. Single-ion activities require a stated convention, and this simplified equilibrium does not resolve all solvent-separated pairs, contact pairs, aggregates, or adsorption equilibria. It does not apply unchanged to salts with multivalent counterions, such as sulfate. The pore and bulk descriptors are comparable only under compatible standard-state definitions.

Geometric access does not eliminate the energetic cost of coordination rearrangement. Li^+^, Na^+^, and Zn^2+^ must be compared within their own solvation, valence, and wall-interaction conditions. Polymer-regulated Na solvation [271], hydrogen-bond solvation modules [272], anion anchoring in Zn electrolytes [273], and defect-associated Li trapping [274] are distinct interventions; none supplies a universal measured value of *K*_d,pore_ for Equation (6).

Resolve speciation with Raman/IR, NMR, XAS, dielectric measurements, or appropriately constrained simulation; pair the assignment with transport on the same physical state.

Confinement can promote dissociation or selective exchange but can also increase exclusion, association, or immobilization. A nonmonotonic response is therefore possible, not a universal optimum or an inevitable ordering of free ions, pairs, and aggregates.

**Figure 5:**
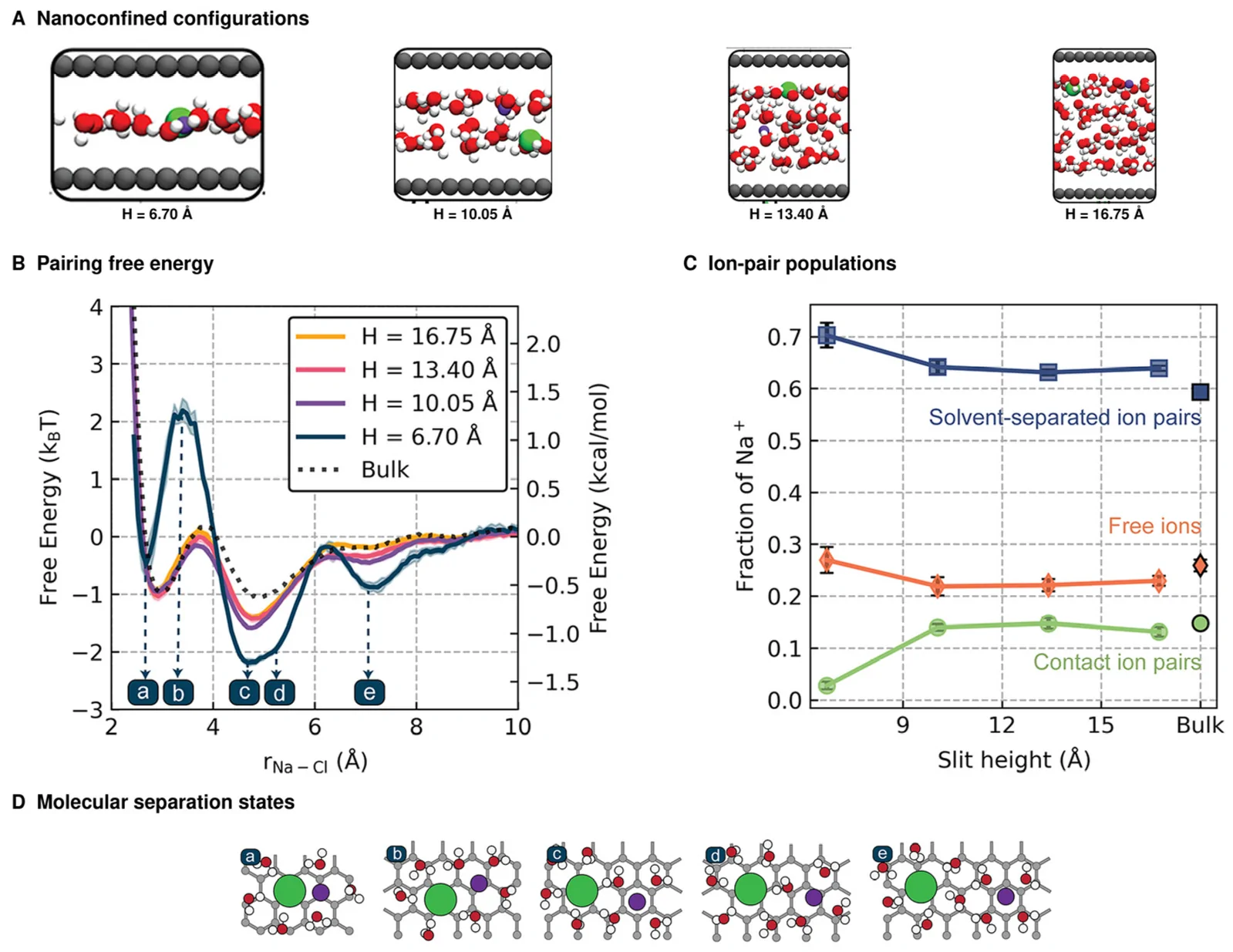
Published molecular-level evidence for nanoconfined ion pairing. (**A**) Simulation snapshots at four graphene slit heights, cropped from Figure 1 in Ref. [44]. (**B**,**C**) Potentials of mean force and sodium-ion-pair populations from Figure 2A,B in Ref. [44]. (**D**) Molecular configurations at separations marked a–e in (**B**), from Figure 2E in Ref. [44]. Purple and green spheres represent Na^+^ and Cl^−^, respectively; red, white, and gray spheres represent oxygen, hydrogen, and carbon. The lowercase labels a–e in (**D**) correspond to the separations marked a–e on the free-energy profile in (**B**). Curve colors and pairing-state colors are identified by the original legends and labels. These are machine-learning molecular-dynamics results for aqueous NaCl, not microscopy or measured battery–electrolyte spectra. Source axes, energy units, curves, and error bars are retained; redundant source panel letters have been removed. Cropped, proportionally resized, and rearranged using Python 3.12.14 and Pillow 12.3.0. © 2024 The Authors; published by American Chemical Society. Adapted under CC BY 4.0, https://creativecommons.org/licenses/by/4.0/ (accessed on 19 October 2026).

Figure 5 replaces a generic speciation sketch with a source-specific molecular example: aqueous NaCl in graphene slit pores [44]. Published molecular-dynamics snapshots, potentials of mean force, and ion-pair populations link slit height, coordination, and association in that model. They are computational results, not microscopy, and cannot be transferred quantitatively to Li- or Zn-based battery electrolytes. Neither stronger confinement nor cation valence fixes a universal direction of the speciation shift.

### 10.4. Ion-Specific Trapping and Relaxation Evidence

Candidate retention sites include coordination groups, fixed charges, defects, pore necks, dead ends, and interfaces. Their transport role depends on the retained species and release kinetics. Aqueous Zn channel layers and biomaterial hydrogels illustrate proposed pathway-selectivity mechanisms [246,275,276,277]. 

A non-battery comparison is supplied by chelating hyperbranched polyester nanoparticles: Alregeb et al. measured Cd(II) uptake as a function of contact time, concentration and temperature, and examined desorption [278]. The study illustrates the value of testing uptake and release separately. Its aqueous metal-removal results do not establish through-membrane Li^+^, Na^+^, or Zn^2+^ conduction, nor an electrically rewritten state; adsorption capacity must not be substituted for useful ionic mobility.

For a thermally activated release process,

(7) $$k_{off,i}=\nu_{i}exp(\frac{-{\Delta G}_{rel,i}^{‡}}{k_{B}T});\tau_{res,i}={k_{off,i}}^{-1}$$

In Equation (7), *k*_off,*i*_ is an effective first-order release rate (s^−1^), *ν_i_* is an attempt-frequency prefactor (s^−1^), Δ*G*_rel,*i*_^‡^ is a release activation free energy per ion (J), *k*_B_ is the Boltzmann constant (J K^−1^), and *τ*_res,*i*_ is the mean residence time (s) for a single exponential escape process at temperature *T*. A molar activation free energy would require RT in the denominator instead of *k*_B_T. This kinetic ansatz distinguishes a release barrier from equilibrium adsorption energy; heterogeneous sites and nonexponential survival require distributions or an explicitly stated effective rate.

Selective counterion retention can favor cation transport only if cation exchange remains fast and adverse space charge or depletion is limited. Long cation residence can instead reduce flux. For Zn^2+^, enrichment and partial desolvation must be distinguished from slow ligand exchange and immobilization.

Heterogeneous sites can produce broad relaxation spectra and observation-time dependence. The sources reviewed do not establish one trap-depth spectrum or energy boundary transferable across Li, Na, and Zn electrolytes.

**Figure 6:**
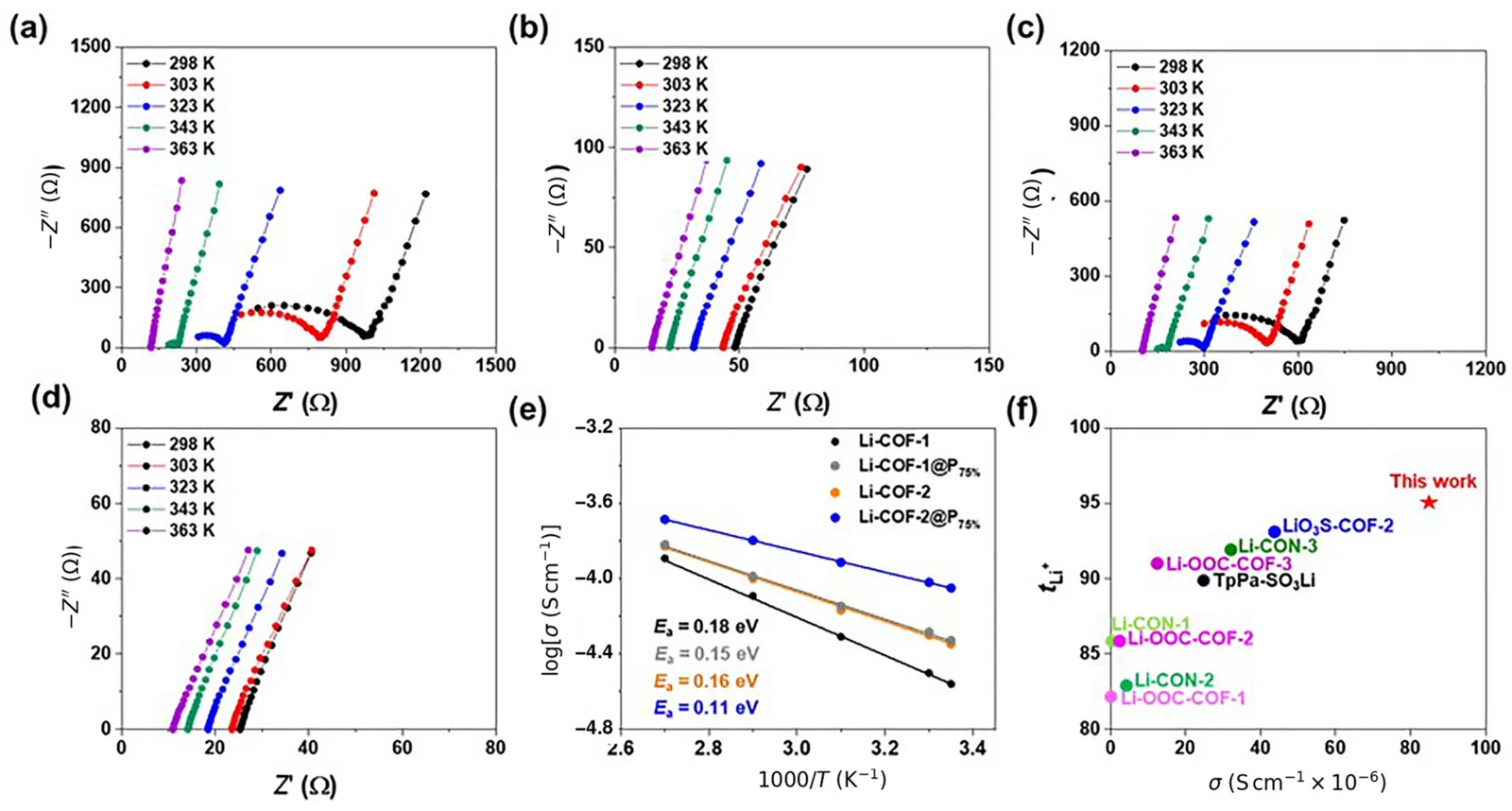
Experimental transport characterization of Li-COF composites, adapted from Figure 2a–f of Ref. [36]. (**a**–**d**) Temperature-dependent impedance spectra of Li-COF-1, Li-COF-1@P75%, Li-COF-2, and Li-COF-2@P75%, respectively, at 298–363 K. (**e**) Conductivity Arrhenius plots. (**f**) The source authors’ comparison with previously reported COF single-ion conductors. Colored text and markers in (**f**) identify the labeled electrolyte compositions; they do not represent an additional quantitative variable. The red star labeled “This work” denotes the material reported in Ref. [36], not a new measurement in the present review. The published data, panel labels, legends, and numerical values are preserved. Axis labels and minus signs have been retypeset using Matplotlib 3.10.8; layout preparation and proportional resizing used Python 3.12.14 and Pillow 12.3.0. Activation energies for conductivity do not directly measure ionic residence times. © The Author(s) 2024; CC BY 4.0, https://creativecommons.org/licenses/by/4.0/ (accessed on 19 October 2026).

Figure 6 provides a complementary experimental transport example: temperature-dependent impedance and conductivity in nominally solvent-free Li-COF and PEGDA-containing Li-COF composites [36]. These measurements constrain macroscopic transport activation, but the fitted conductivity activation energies are not independently measured release barriers or residence times in Equation (7). Coordination/trapping studies [149,274,279] and hydrophobically gated nanopores [280] concern different mechanisms and physical states; they cannot supply a universal trap-depth or retention threshold. Species-specific exchange and field-off tests remain necessary before assigning reversible ionic memory.

### 10.5. Nonlinear and Collective Ion Transport

A nonlinear loading response can reflect geometric percolation, site filling, association, or cooperative motion. These mechanisms require different observables. Polymer-state changes provide additional alternatives [281,282]. Anion-retaining cage electrolytes [37], cation-selective Zn hydrogels [81], defect traps [274], and oxygen-vacancy-assisted dissociation/trapping [279] address species regulation and are not by themselves a geometric percolation threshold.

First, geometric percolation concerns a sample-spanning conducting network. Near a threshold, and where an appropriate network model supports scaling, a simplified relation is

(8) $$\sigma_{eff}\propto {\left(\varphi -\varphi_{c}\right)}^{m_{perc}},\;\;\;\varphi >\varphi_{c}$$

In Equation (8), *φ* is the volume fraction of the candidate conducting phase, *φ*_c_ is its geometric percolation threshold, and *m*_perc_ is a dimensionless conductivity-scaling exponent. All fractions are defined for the same geometry and physical state. The proportionality coefficient has conductivity units; parallel conduction through another phase can require a background term. A loading-dependent conductivity change alone does not identify a threshold or an exponent. Trap filling and, at higher occupancy, ion association or crowding are separate possible crossovers and must not be conflated with geometric percolation.

A pore-network charging model [9] supplies a topology-dependent transport example, not a measured battery percolation exponent. Other nonlinear mechanisms include paddle-wheel-assisted Na conduction [283], convection-assisted porous-electrode charging [284], ion correlations in nonaqueous nanopores [285], and electrochemical doping [286]. Their observables and physical states differ; none establishes the proposed occupancy window for all battery electrolytes.

Accordingly, a conductivity maximum may reflect changes in both carrier availability and mobility. Its origin must be resolved from composition, speciation, connected volume, and relaxation rather than assigned to an occupancy optimum from curve shape.

**Figure 7:**
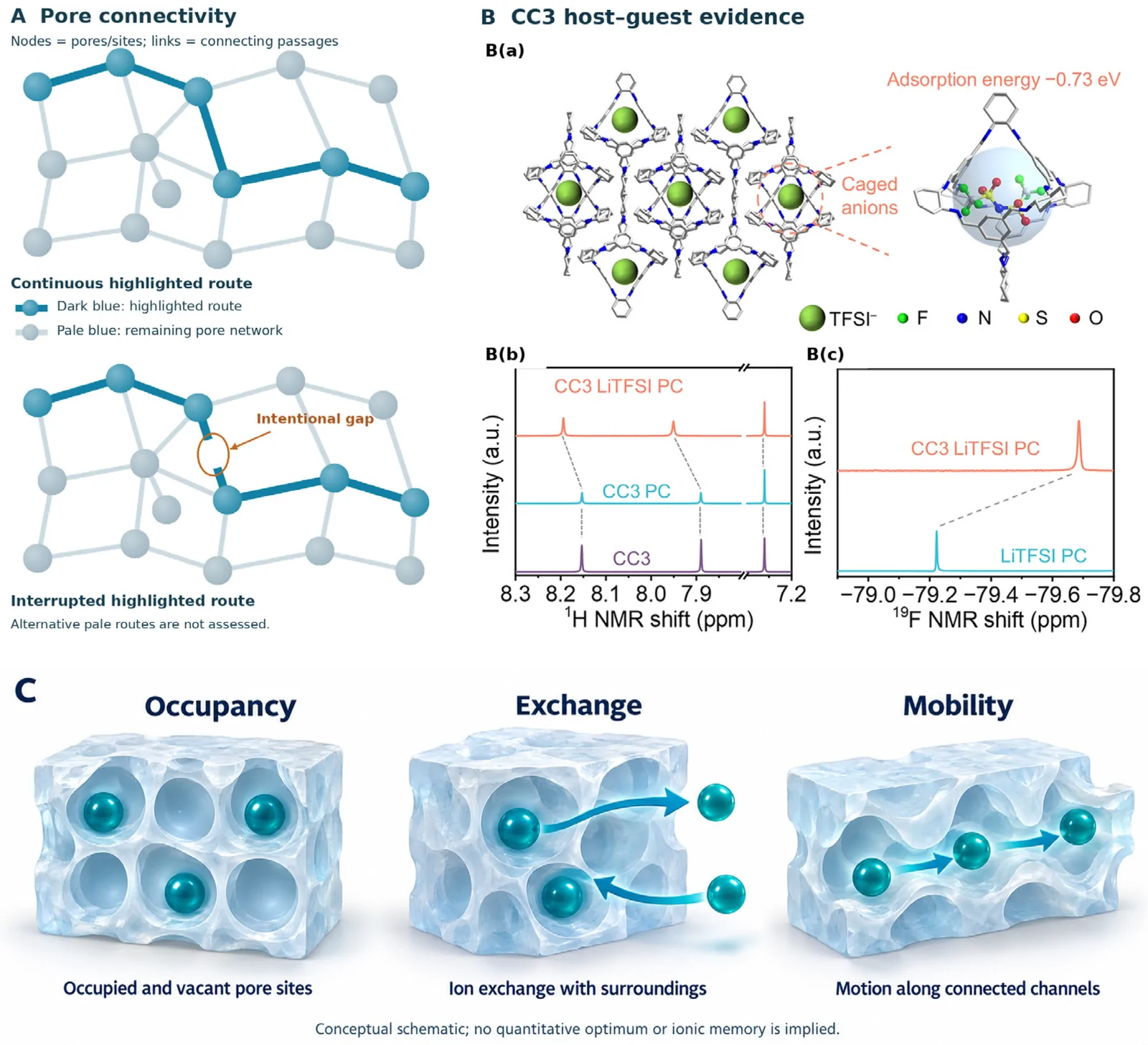
Connectivity, host–guest interactions, and conceptual transport controls. (**A**) Conceptual pore networks: nodes represent pores or sites, and links represent connecting passages, not atoms or chemical bonds. Dark blue highlights a candidate transport route; pale blue shows the remaining network. The upper highlighted route is continuous. The intentional gap in the lower highlighted route, marked by the orange ellipse and arrow, interrupts that route; connectivity through alternative pale routes is not assessed. Colors do not distinguish chemical species. (**B**) Adapted from Figure 3a–c of Qin et al. [37]. (**B(a)**) CC3 packing and the calculated TFSI^−^ adsorption energy within the cage cavity (−0.73 eV). (**B(b)**) ^1^H NMR spectra of CC3, CC3 with propylene carbonate (PC), and CC3 with LiTFSI in PC, measured in CDCl_3_. (**B(c)**) ^19^F NMR spectra of LiTFSI in PC with and without CC3, measured in CDCl_3_. Gray dashed lines connect corresponding resonances. Original spectra, numerical values, and units are retained; source subpanels a–c are relabeled (**B(a)**–B**(c)**). (**C**) Qualitative schematics of site occupancy, exchange between a pore and its surroundings, and ion mobility along a conducting pathway. The translucent pale-blue scaffold represents the porous host, teal spheres represent ions, and arrows indicate exchange or motion. Occupied and vacant cavities illustrate site occupancy. No quantitative optimum or ionic-memory response is implied. (**A**) was drawn with Python 3.12.14 and Pillow 12.3.0. The original three-dimensional conceptual artwork in (**C**) was prepared with ImageGen (OpenAI; hosted service, build identifier not recorded), reviewed by the authors, and assembled with Python/Pillow. (**B**) was proportionally resized and its subpanels relabeled. (**B**): © The Author(s) 2024; CC BY 4.0, https://creativecommons.org/licenses/by/4.0/ (accessed on 19 October 2026).

Figure 7 separates conceptual transport controls from source-specific CC3 host–guest evidence. In panel A, nodes represent pores or sites rather than atoms; dark blue highlights a candidate transport route and pale blue denotes the remaining network. The lower gap intentionally interrupts the highlighted route, without establishing whether alternative pale routes can support transport. Panels B(a)–B(c) combine a calculated host–guest configuration with solution-state NMR evidence for CC3–TFSI^−^ interactions [37]. The three-dimensional conceptual views in panel C illustrate occupancy, exchange, and mobility: the pale-blue scaffold represents the porous host, teal spheres represent ions, and arrows indicate exchange or motion. These panels do not quantify an operating-film transport network, establish a universal optimum in occupancy, or demonstrate field-off retention and electrical rewriting. Any occupancy optimum must be measured for a defined material, composition, and operating condition.

Cooperative motion occurs when one ion changes another’s migration probability. Angstrom-scale channel evidence [287] motivates this possibility but does not establish a universal cooperative threshold in bulk battery electrolytes.

To distinguish the mechanisms, combine increasing/decreasing composition sweeps with matched temperature, pressure, and mobile-phase inventory, plus species-sensitive and structural observations. Test heating, phase change, fracture, interphase growth, and electrode polarization. Reserve separate terms for percolation, trap filling, cooperative crossover, and aggregation onset.

### 10.6. Evidence for History-Dependent Ionic States

Concentration polarization, adsorption, occupancy, wetting, and interfacial rearrangement can relax slowly after stimulation, leaving transient history dependence. Droplet synapses and biological/artificial nanopores provide non-battery examples [47,288,289,290,291,292].

Define the operational sequence once as write–wait–read–erase (WWRE): prepare the state, impose a specified field-off wait, read with bounded disturbance, and test reversible recovery. Distinct written histories must remain distinguishable under identical read conditions. State whether waiting is open circuit or held at zero applied voltage, since these boundaries can yield different relaxation [30,31,293]. These papers motivate device-specific tests; the common WWRE acceptance criteria are proposed here and are not claimed to have been demonstrated in every cited device.

Nanofluidic memory studies [30,31,32,293,294,295,296,297,298] must be distinguished from concentration–polarization coupling [299], voltage-gated pore chemistry [300], confined-transport theory [301], and ion-selective transistor or logic devices [302,303]. Cooperative transport [287] is likewise not synonymous with a retained memory state. Each source supports its own geometry, observable, and timescale; a shared ionic mechanism or full compliance with Table 8 is not presumed.

Poletayev et al. provide a separate solid-state result: terahertz pump–probe measurements and simulation resolve decay of orientational correlations between ionic hops in β-aluminas on picosecond timescales [304]. This microscopic transport memory is an established, source-specific use of the term. It is distinct from the reversibly addressable retained state targeted by our programmable-memory protocol; the optical result does not demonstrate repeated electrical WWRE operation in a bulk battery electrolyte.

Transfer to a battery membrane requires controls for its own electrodes, dimensions, fields, and mobile phase. Interphase formation, Zn corrosion, solvent redistribution, heating, cracking, contact drift, electronic leakage, and metallic penetration can mimic a retained ionic response [23,28,30,188,297,305].

Cathodic polarization of porous silicon supplies a complementary interfacial counterexample. Yakovtseva et al. discuss space-charge and surface/contact effects together with erbium–hydroxide deposition promoted by local alkalization [306]. It is a semiconducting porous electrode in solution, not a bulk battery electrolyte. Its relevance here is to distinguish electrode-driven chemical deposition and contact changes from a reversibly retained electrolyte state.

Pandey et al. found electrode-related hysteresis in membrane-free Ag/AgCl controls, while the tested two-dimensional channels retained characteristic loops with other electrodes [305]. This device-specific result motivates electrode substitution and blank controls; it neither validates nor excludes programmable memory in bulk battery electrolytes.

**Figure 8:**
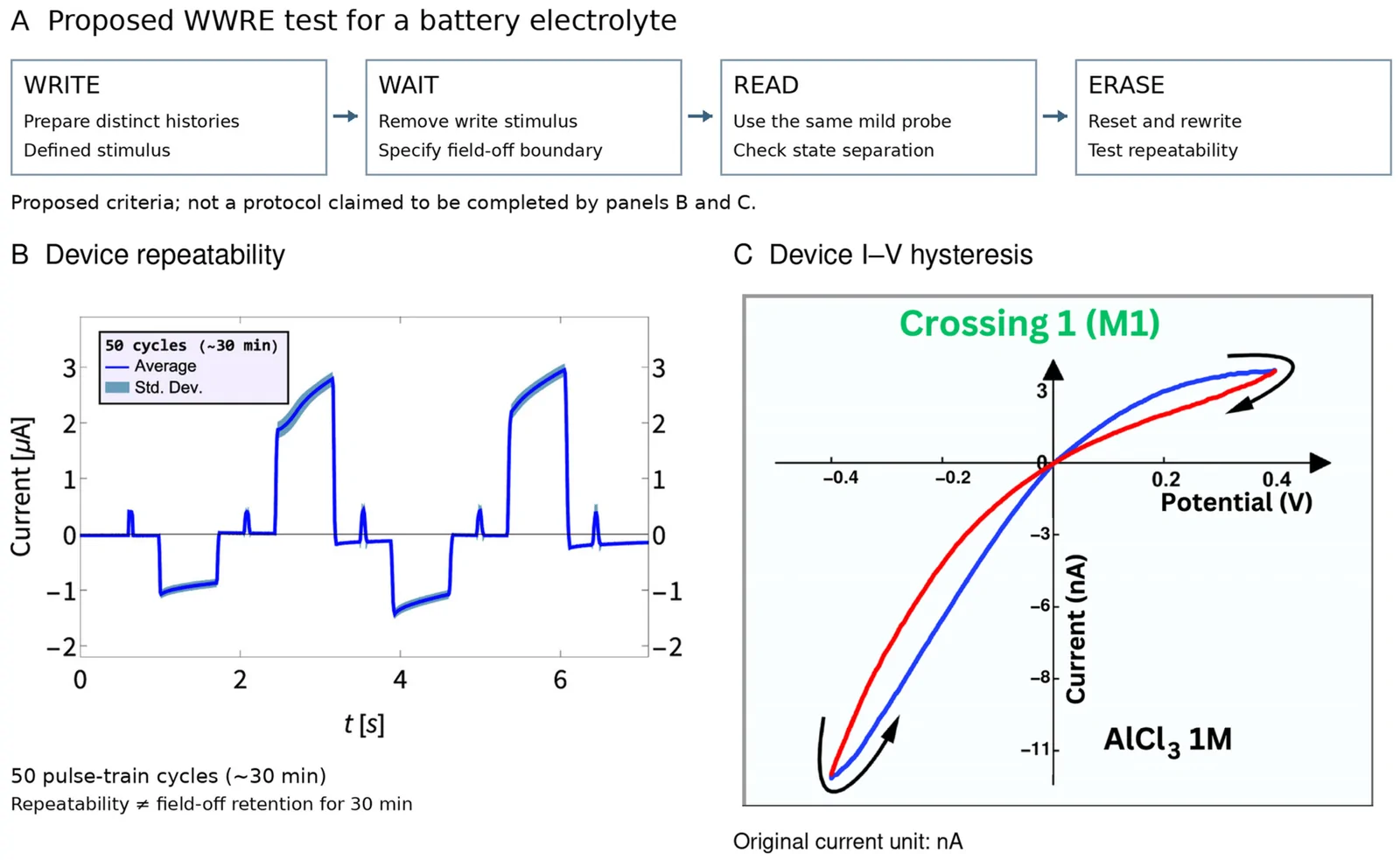
Proposed validation sequence and separate nanofluidic device examples. (**A**) Original conceptual WWRE sequence: write–wait–read–erase; field-off waiting and minimally disturbing readout are separate operations. (**B**) Figure 2 in Ref. [31]: mean current (blue line) and standard-deviation band over 50 pulse-train cycles (~30 min) in an aqueous-KCl colloidal nanochannel device. Write pulses are −2 and 5 V; read pulses are 1 V. (**C**) Figure 1d in Ref. [32]: crossing-type I–V loop in a 2-nm hBN channel with 1 M AlCl_3_. Blue and red distinguish the two branches of the loop, and black arrows indicate the direction of traversal; the original current unit, nA, is retained. The original lowercase source label d has been removed, and minus signs in tick labels have been retypeset. Panel (**B**) is reproduced with proportional resizing; (**C**) is cropped, relabeled, and proportionally resized. © The Author(s) 2024 (**B**) and 2025 (**C**); both CC BY 4.0, https://creativecommons.org/licenses/by/4.0/ (accessed on 19 October 2026). Panel A and the composite layout were prepared using Python 3.12.14 and Pillow 12.3.0; B and C are published data, not generated curves.

Figure 8 separates the proposed battery–electrolyte WWRE test from two published nanofluidic device observations. Kamsma et al. [31] report repeatability over 50 pulse-train cycles in approximately 30 min; this duration is not a field-off retention time. Ismail et al. [32] report a device-specific I–V loop with current in nA. These selected panels illustrate repeatability and hysteresis, respectively; neither alone verifies the full WWRE criteria or establishes ionic memory in a bulk battery electrolyte.

Table 8 sets the evidence boundary for temporal claims. The battery observations [23,28,188] and device/optical results [30,31,32,304] retain their original specimen and timescale limits; missing controls remain unverified.

## 11. Authors’ Perspective: A Testable State-Aware Framework

The descriptors below organize operating-state measurements and competing explanations. They are prospective comparison tools, not fitted constitutive laws. Section 12 gives discriminating tests; Table 8 governs temporal claims.

### 11.1. Proposed Link Between Pore Architecture and Internal Ionic State

Architecture can affect speciation, occupancy, charge, and mobile-phase partitioning. Representing these through an internal state is useful only if measured variables explain a matched transport change; it does not establish universal coupling or persistence.

Separate quasi-static architecture from time-dependent ionic variables schematically as

(9) $$P=\{d_{b},\varepsilon_{acc},G_{arch},q_{wall},\varphi_{mob}\}\;S(t)=\{c_{free},\chi_{spec},\theta_{pore},\theta_{trap},\rho_{sc}\}$$

In Equation (9), *P* is a structural/physical-state descriptor set, and *S*(*t*) is a time-dependent internal ionic-state vector. *d*_b_ is the accessible bottleneck size (m), *ε*_acc_ is the accessible conducting-phase volume fraction, *G*_arch_ is the aggregate architecture factor from Equation (4), *q*_wall_ is a specified pore-wall surface-charge density (C m^−2^), and *φ*_mob_ is the mobile-phase volume fraction. *c*_free_ is an operationally defined free-carrier concentration (mol m^−3^), *χ*_spec_ is a vector of dimensionless speciation fractions, *θ*_pore_ and *θ*_trap_ are dimensionless pore and trap occupancies, and *ρ*_sc_ is net space-charge density (C m^−3^). Structural entries may be correlated; listing both *ε*_acc_ and *G*_arch_ does not license multiplying them again. The partition treats these descriptors as quasi-static over the chosen measurement interval; swelling, wall-charge regulation, or mobile-phase redistribution may require their explicit time dependence. This state vector is a conceptual selection of variables, not a closed evolution model.

(10) σ(t)=F(P, S(t), T, E, I)

In Equation (10), *σ*(*t*) is the time-dependent conductivity under the defined read or measurement protocol, ℱ is a generic response functional, *P* and *S*(*t*) are defined in Equation (9), *T* is temperature, *E* is electric field (V m^−1^), and ℐ identifies ion chemistry and composition. The calligraphic ℱ is distinct from the Faraday constant *F*, and ℐ is a label rather than electrical current. Equation (10) organizes dependencies without supplying a predictive constitutive law. A transient current cannot automatically be called conductivity unless capacitive and interfacial contributions are resolved.

### 11.2. Proposed Carrier–Bottleneck Matching Descriptor

Use the effective species approaching the limiting passage, rather than a bare crystal radius, to define a candidate carrier–bottleneck comparison.

A useful conceptual descriptor is the confinement-matching ratio

(11) $$\Lambda_{i}=\frac{d_{eff,i}}{d_{b}}$$

In Equation (11), *Λ_i_* is the proposed dimensionless carrier–bottleneck ratio, *d*_eff,*i*_ is an effective transported-species diameter, and *d*_b_ is the limiting accessible bottleneck size in the same length units. *d*_eff,*i*_ depends on solvation, coordination, association, temperature, and the operational definition used. Values near unity indicate a geometric size comparison, not a universal conductivity optimum or guaranteed entry. Flexible coordination shells and anisotropic openings can require a distribution rather than one diameter.

Equal bottleneck sizes can have opposite consequences because wall charge, dielectric response, and coordination chemistry change entry and escape barriers. Evaluate this descriptor within a specified Li, Na, or Zn electrolyte; geometric matching alone cannot predict favorable transport.

### 11.3. Geometric Connectivity and Hypothesized Dynamic State Coupling

Geometric connectivity identifies a continuous accessible route. Dynamic coupling asks whether a local ionic perturbation influences another region before relaxing.

A percolating conductor can remain dynamically local. To compare communication and local relaxation over matched length scales, define

(12) $$C_{dyn}=\frac{\tau_{relax}}{\tau_{comm}}$$

In Equation (12), *C*_dyn_ is a proposed dimensionless dynamic-coupling descriptor, *τ*_relax_ is the decay time of a specified local ionic perturbation, and *τ*_comm_ is the time for that perturbation to influence a specified neighboring region (both in s). The observable, length scale, and physical state must be common to the two times. A value near or above unity only makes communication before relaxation plausible; it does not establish a collective transition or memory. No universal critical value is asserted.

Percolation and temporal coupling need not share a threshold. A claimed coupling crossover requires spatial or length-dependent transient evidence beyond the conductivity-loading curve (Section 12.2).

### 11.4. Hypothesized Finite Transport Window

Architecture, available-carrier fraction, and mobility can change at different compositions. Their competition motivates a possible finite transport window whose existence and location require testing. The separate nonlinear mechanisms reviewed in Section 10.5 do not constitute experimental validation of a universal occupancy optimum.

A restricted screening expression avoids subtracting overlapping trapped, paired, and aggregated populations as if each were independently immobile:

(13) $$\sigma_{M,screen}\approx G_{arch}\left|z_{M}\right|F\;c_{M,avail}\;u_{M},\;c_{M,avail}=f_{M,avail}\;c_{M,total}$$

In Equation (13), *σ*_M,screen_ is the screening quantity of Equation (5), *G*_arch_ is the aggregate architecture factor, *z*_M_ is the target-cation charge number, and *F* is the Faraday constant. *c*_M,ava*i*l_ and *c*_M,total_ are target-cation concentrations within the local conducting phase (mol m^−3^); *f*_M,ava*i*l_ is an operationally defined available fraction between 0 and 1; and *u*_M_ is positive electrical drift mobility within the local conducting phase (m^2^ V^−1^ s^−1^), excluding the architectural reduction already represented by *G*_arch_. The expression therefore has conductivity units (S m^−1^). It is a single-population, weak-correlation screening approximation, not a general conductivity law. Association and trapping can affect both availability and mobility, and paired or aggregated species are not assumed universally immobile. Available fractions must be defined without overlapping population counts. Where charged clusters or cross-correlations contribute appreciably, species-resolved or concentrated-solution transport is needed; the factors must not be fitted to the same conductivity and then treated as independent mechanistic evidence.

The hypothesized window combines connected access, available carriers, reversible exchange, and limited crowding. It is not guaranteed, and memory is not a required outcome. Figure 7 separates conceptual controls from source-specific host–guest evidence without plotting an optimum. Section 12.3 distinguishes possible occupancy effects from changes in viscosity, polymer relaxation, or liquid fraction.

### 11.5. Species-Specific Residence Times and Proposed Kinetic Selectivity

Express site residence relative to the operation being evaluated through

(14) $$K_{i}=\frac{\tau_{res,i}}{\tau_{op}}$$

In Equation (14), *K_i_* is the proposed dimensionless kinetic trapping ratio, *τ*_res,*i*_ is the residence time of the named species and site population, and *τ*_op_ is the chosen operational, observation, or stimulation time (both in s). The interpretation changes with that selected time window. A large ratio suggests retention over that window but cannot by itself distinguish useful counterion retention from detrimental target-cation immobilization or establish reversible memory.

A useful kinetic asymmetry retains counterions while allowing rapid target-cation exchange. The reverse can suppress flux; equilibrium binding strength alone cannot choose between these outcomes.

Interpret this ratio for the named species, coordination environment, and measurement window. The chemistry-specific controls in Table 6 apply; no universal Li/Na/Zn residence–time ranking is assumed.

### 11.6. From History-Dependent Transport to Programmable Ionic States

A candidate state may evolve during electrical stimulation and affect a later measurement. Represent that prospective dependency formally as

(15) $$\frac{dS}{dt}=G(S,\;E,\;T;P)$$

In Equation (15), *dS*/*dt* is the time derivative of the state vector, 𝒢 is an unspecified state-evolution operator, *E* is applied electric field, *T* is temperature, and *P* is the descriptor set. Each component of 𝒢 has the units of its corresponding state variable per unit time; the mixed-unit state vector is not one scalar observable. The relation is a formal organizing statement, not a solved kinetic model. Field removal may leave slow relaxation, which must still pass the WWRE and attribution tests before it is called programmable ionic memory.

Use the claim hierarchy in Table 8: transport without a resolved retained state, dynamic gating, history dependence, persistent reversible state, and programmable ionic memory. These are evidence categories, not an inevitable sequence of material behavior.

A useful timescale condition is

(16) $$\tau_{read}\ll \tau_{state}\ll \tau_{irr}$$

In Equation (16), *τ*_read_ is the duration of the minimally disturbing read operation, *τ*_state_ is a characteristic lifetime of the written ionic state, and *τ_i_*_rr_ is the characteristic time for irreversible chemical, structural, or electrochemical change (all in s). The inequality is a desirable timescale separation, not proof of memory and not a universal threshold. Retention must be measured over explicitly stated field-off waiting times and compared with the readout noise; read duration alone does not specify retention. Repeated writing and reading must also be checked for cumulative irreversible change.

Nanofluidic and iontronic observations motivate the proposed tests [30,31,32,287,293,294,295,296,299,300]; they do not establish a distributed, reversibly programmable state in a bulk battery membrane.

**Figure 9:**
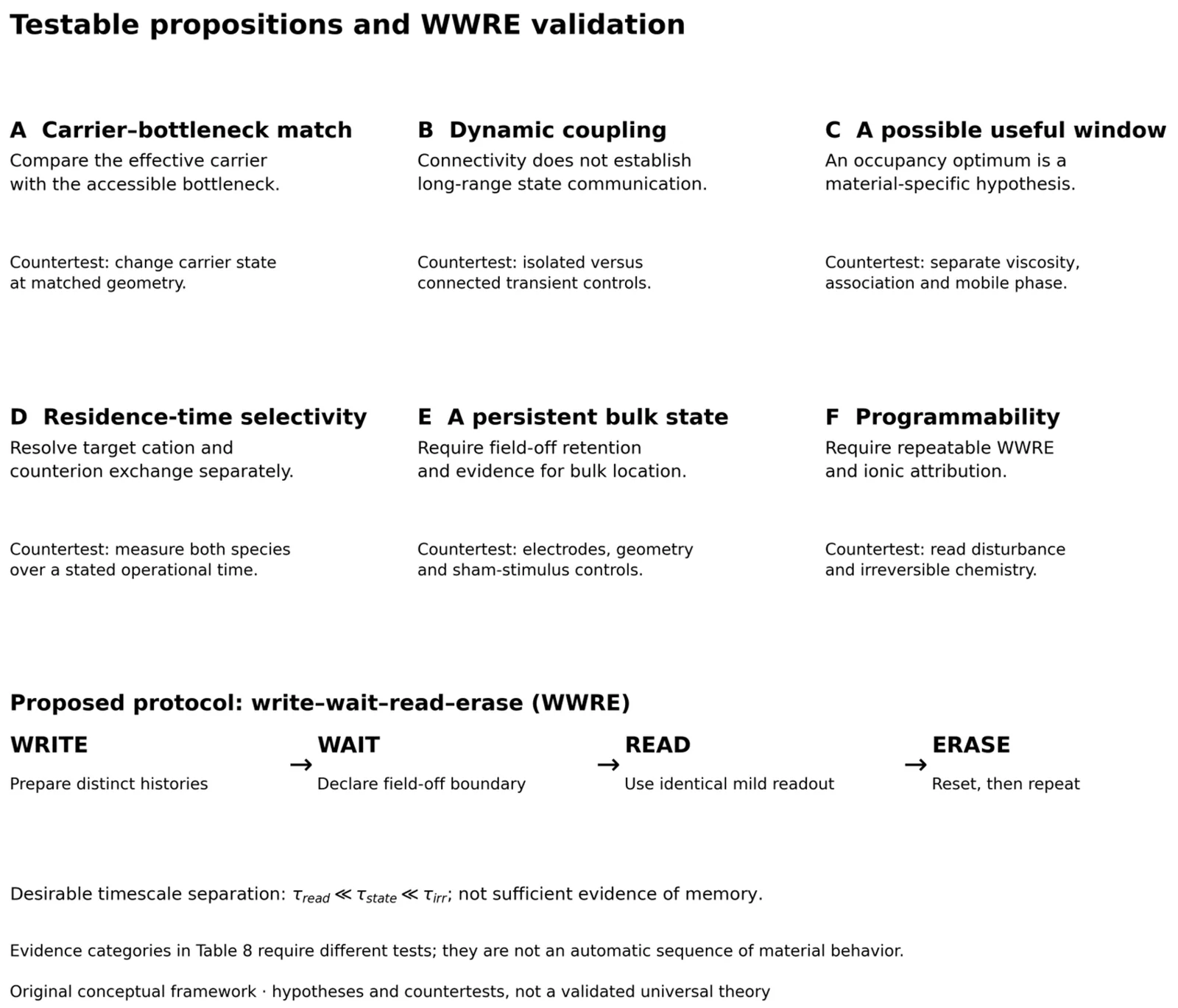
Original synthesis of six testable propositions and countertests aligned with Table 9: carrier–bottleneck matching, dynamic coupling, a possible useful transport window, residence–time selectivity, a persistent bulk state, and programmability. The protocol is write–wait–read–erase (WWRE), with a declared field-off wait and an identical minimally disturbing readout. The timescale separation *τ*_read_ ≪ *τ*_state_ ≪ *τ_i_*_rr_ is a desirable criterion, not sufficient evidence of memory. The propositions do not constitute a validated predictive theory, and Table 8 evidence categories are not an automatic sequence of material behavior. Prepared with Python 3.12.14 and Matplotlib 3.10.8; no experimental panel or universal optimum curve is reproduced. Coding assistance was provided by Codex (OpenAI).

Figure 9 organizes the six propositions and countertests in Table 9 as a research agenda. Geometric matching does not guarantee fast entry, connectivity does not establish dynamic coupling, and a finite useful occupancy window remains a material-specific hypothesis. Persistent bulk states and programmability require the WWRE and ionic-attribution tests; these propositions do not constitute a validated predictive theory.

### 11.7. Perspective Summary and Transition to Validation

Established access, speciation, and exchange mechanisms motivate the descriptors. Their proposed coupling and extension to bulk-electrolyte memory remain hypotheses.

Evaluate each proposition against a simpler explanation and a defined failure outcome. Section 12 specifies the tests; satisfying a descriptor alone does not advance a claim up Table 8.

## 12. Falsifiable Hypotheses, Validation, Reporting Standards, and Research Priorities

Each hypothesis below is paired with an intervention, matched controls, and an outcome that would weaken it. A negative persistence result does not invalidate evidence for useful steady transport.

### 12.1. Testing Carrier–Bottleneck Matching

**H1:**  *Vary operating-state bottlenecks while matching salt activity, mobile-phase amount, thickness, and host chemistry. Establish accessible geometry after conditioning and resolve the approaching coordination state. A chemistry-dependent shift in the response supports a carrier–bottleneck effect; a trend explained entirely by uptake or contact does not.*

### 12.2. Separating Geometric Percolation from Dynamic State Coupling

**H2:**  *Compare connectivity series at matched chemistry and mobile-phase inventory. Combine directional structure/access with thickness-dependent transients, relaxation, and blocked-channel controls. A distinct temporal crossover that follows network communication supports coupling; an electrode or local-polymer relaxation that explains the full signal weakens it.*

### 12.3. Distinguishing Trap Filling, Collective Coupling, and Aggregation

**H3:**  *Measure site occupancy, free/associated species, accessible volume, and ion-specific diffusion across a loading series at matched temperature and solvent content. Distinct site-filling and aggregation changes can support a finite window; a maximum tracking only viscosity, polymer relaxation, or mobile-phase fraction does not identify occupancy-driven transport.*

### 12.4. Measuring Species-Specific Residence–Time Landscapes

**H4:**  *Compare wall chemistries with similar access but different exchange kinetics. Resolve both cation and counterion residence distributions under the actual salt/solvent conditions, and test simulation predictions against measurement. Selectivity consistent with rapid cation exchange supports kinetic asymmetry; persistent binding without flux identifies immobilization.*

### 12.5. Demonstrating a Persistent Internal Ionic State

**H5:**  *Apply WWRE under bounded electrode reactions, heating, leakage, and contact drift. Vary write dose/polarity, field-off wait, and thickness; repeat unwritten controls and read-disturbance tests. An independent ionic-state marker strengthens attribution. A response present only during bias is gating; an irreversible retained change is conditioning.*

### 12.6. Validating Ionic Memory and Rejecting Artifacts

**H6:**  *Require repeated WWRE with recoverable state separation, controlled read disturbance, and independent-sample reproducibility. Use electrode substitution/blanks, thermal and mobile-phase controls, and chemically matched dense or pore-blocked membranes where feasible. Operando evidence helps locate the state but does not replace these controls [305,307,308]. If readout creates the apparent state, or irreversible/thermal changes explain it, programmable ionic memory is not established for that specimen.*

Report state separation and scatter, retention relative to noise, write energy, read disturbance, endurance, polarity, and temperature dependence across cells/batches. Specify the tested waiting boundary and recovery criterion; a failed test constrains retention only over its measured resolution and time window.

### 12.7. Decision Matrix for the State-Aware Framework

Table 9 links the six propositions to their main countertests. It permits selective memoryless conduction as a valid outcome and separates a useful transport mechanism from a programmable-state claim.

Reproducibility requires the following record for the actual electrochemical specimen.

### 12.8. Characterization and Reporting Standards

Measurements on precursor powders provide context; characterize the fabricated and conditioned membrane wherever access or mobile-phase content can change.

#### 12.8.1. Structural Architecture and Physical State

Report thickness, density, accessible volume/bottlenecks, orientation, surface chemistry, and measurement uncertainty in the tested dry, hydrated, swollen, or salt-loaded state. For layered specimens, give constituent positions/volumes relative to current direction.

#### 12.8.2. Mobile-Phase Inventory, Carrier Identity, and Speciation

List salt, solvent, ionic liquid, water, and residual processing species, including changes during conditioning. Support coordination/association assignments with ion-sensitive observables and pair them with transport in the same state.

#### 12.8.3. Transport, Trap, and Relaxation Reporting

State geometry, electrodes, area, thickness, temperature, conditioning, measurement window, fitting, and uncertainty. Identify the transference/diffusion method and reference frame. For trapping, specify species, sites, and timescale; distinguish affinity from release kinetics.

#### 12.8.4. Threshold, Interface, and Memory Claims

Distinguish percolation, saturation, association, and nonlinear crossovers using defined observables and sampling resolution. For cell results, give current density, areal capacity, active inventory, pressure, temperature, electrolyte amount/thickness, and failure criterion.

For temporal claims, report the complete WWRE sequence and the Table 8 controls, including wait boundaries, polarity, duration, energy/charge, read disturbance, drift, and replicate variation. Relate retention to the intended operating timescale and demonstrate reversible recovery.

#### 12.8.5. Minimum Reproducibility Matrix

Table 10 condenses this record by claim. No single technique satisfies every evidentiary requirement.

### 12.9. Research Priorities and Practical Translation

Prioritize matched operating-state series that separate bottleneck, wall chemistry, connectivity, and mobile-phase fraction using the chemistry-specific controls in Table 6. Pair species-resolved exchange with three-dimensional access and directional transport. Translation also requires reproducible full-cell and life-cycle assessment [43,307]. Zn additives and artificial interphases [309,310,311], and Li additive strategies or coupled fluorination [312,313], offer separate interface interventions; their cycling gains do not validate the proposed bulk-memory framework.

Carry candidate mechanisms into cells with reported loading, active inventory, thickness, pressure, and electrolyte quantity. A liquid-rich membrane and a dry solid address different physical-state constraints.

Test persistent states first in controlled nonfaradaic configurations with independent ionic markers. Extend to batteries only after location, field-off retention, and reversibility are established. Computation should prioritize matched interventions and measurable predictions, not infer memory from static adsorption energies.

## 13. Significance, Scope, and Limitations of the State-Aware Framework

### 13.1. Significance of the Framework

This review contributes a critical synthesis and claim-dependent evidence hierarchy. The descriptors in Section 11 organize prospective tests of architectural access, carrier chemistry, and temporal response; they do not supply a new validated microscopic theory.

### 13.2. Scope and Domain of Applicability

Applicability lies in shared experimental questions, not equivalent chemistry. Polymer coordination and association complicate Li/Na interpretation; hydrated Zn adds water activity, proton-related pathways, and interfacial reactions (Table 6). Classify each tested membrane by its actual mobile-phase inventory.

### 13.3. Limitations of the Evidence Base

The literature is selective and heterogeneous rather than a pooled dataset. Temperature, pressure, geometry, composition, and ion-specific measurement methods often prevent quantitative ranking. Precursor-powder structure may not describe membrane access, and static images/binding calculations rarely resolve species-specific exchange or post-field relaxation.

Evidence for programmable responses in iontronic devices cannot establish bulk battery–electrolyte memory by analogy. That extension remains open and requires the specimen-specific tests in Section 10.6 and Section 12.

### 13.4. Status of the Proposed Descriptors and Hypotheses

The proposed ratios and windows need operational definitions, independent inputs, uncertainty, and prospective countertests. Revise or abandon a descriptor when a verified intervention leaves the response unchanged or a simpler measured variable explains the trend.

### 13.5. Future Utility of the Framework

The immediate utility is a common measurement record linking physical state, access, species, and timescale. Operando observations on matched specimens can test the proposed couplings. Even without memory, slow relaxation remains relevant to polarization, interfaces, and impedance interpretation after cycling.

## 14. Conclusions

The principal result of this review is a set of conditions for assigning a pore-mediated mechanism. One must locate accessible pores in the operating material, identify the phase carrying ions, and measure transport under conditions that distinguish the pore contribution from polymer morphology, retained liquid, and electrode contact. A conductivity gain by itself establishes a useful system-level change, but it does not resolve its microscopic cause.

Across Li, Na, and Zn systems, the relevant internal state includes carrier speciation, wall occupation, counterion distribution, and the lifetime of exchange sites. These quantities offer testable explanations for why nominally similar pores may promote transport in one composition and restrict it in another. Pore geometry and chemistry act together; neither bare-ion radius nor dry-powder surface area supplies a universal design rule.

The clearest design principle is conditional rather than maximal: preserve an accessible pathway and a sufficient mobile-ion population while avoiding excessively long target-cation residence, crowding, or detrimental interface growth. The most convincing comparisons link a measured structural descriptor with speciation or relaxation and then test the resulting prediction in the battery configuration. Where such links are absent, performance trends remain correlations that deserve narrower interpretation.

The evidence for ionic memory requires a higher threshold than the evidence for history dependence. Persistent conductance must be observed after removal of the writing field, survive beyond the resolved electrode and dielectric transient, and be read without substantial rewriting. Repeated switching, reversal, or erasure, independent-sample replication, and bulk-versus-electrode controls are needed before a programmable electrolyte claim is justified. Existing nanofluidic demonstrations motivate this question but cannot settle it for a solid battery electrolyte.

This framework points to a specific next step for research: combine controlled pore accessibility and connectivity with chemical identification of ionic populations and time-resolved field-off tests. Positive results would establish when architecture supports a reversible internal state; negative results would define where passive conduction or ordinary interfacial polarization remains the adequate description. Both outcomes improve the interpretation and design of solid and quasi-solid energy-storage materials.

## Figures and Tables

**Table 1:** Claim-dependent evidence hierarchy for porous electrolytes.

Question	Minimum Evidence	Competing Cause to Exclude	Design Implication
Where are the active pores?	Operating-state accessibility and direction	Closed voids or dry-powder-only BET area	Target continuous accessible paths
What carries current?	Liquid/water inventory with conductivity	Infiltrated phase mistaken for dry-solid transport	Report physical state alongside performance
Does the wall regulate species?	Speciation/transference and exchange evidence	Salt concentration or bulk polymer effect	Tune wall chemistry and carrier state
Does connectivity couple dynamics?	Independent network descriptor plus relaxation	Conductivity-only percolation	Test temporal response separately
Is an ionic state retained and reversible?	WWRE with quantified retention and read disturbance; see the temporal evidence criteria in Section 10.6	Electrode polarization, thermal drift, electronic leakage, or irreversible conditioning	Require reversible recovery, ionic attribution, and independent-sample replication

**Table 2:** Architecture-specific ambiguities and decisive controls.

Architecture	Dominant Ambiguity	Required Control	Relevant Section
Porous polymer/fiber	Dry segmental versus filled-pore transport	Quantify uptake and test dry/filled membranes	Section 4
Polymer with porous filler	Internal pore versus outer interphase	Pore-accessibility control at matched chemistry	Section 3, Section 4 and Section 5
MOF/COF/cage host	Framework channel versus confined liquid	Inventory solvent and measure ion selectivity	Section 5
Ceramic scaffold	Ceramic skeleton versus infiltrant	Compare filled, empty, and dense controls	Section 6
Glass/disordered solid	Accessible pore versus atomic free volume	Demonstrate accessibility before assigning porosity	Section 7
Hierarchical/Janus stack	Directional route versus interlayer contact	Through-plane and direction-dependent transport	Section 8

**Table 5:** Attribution questions for ceramic-rich and disordered controls.

System Class	Question for Attribution	Measurement to Add	Interpretive Boundary
Porous ceramic conductor	Does current follow ceramic or void phase?	Empty/filled comparison and direction-dependent σ	Porosity can remove conducting cross-section
Infiltrated scaffold	Does the infiltrant dominate?	Quantify uptake and compare neat infiltrant	Framework may be mechanical support
Ceramic-rich polymer composite	Is there a connected interphase?	Network descriptor, crystallinity, transference	Loading trend does not identify pathway
Glassy/disordered control	Is free volume accessible porosity?	Sorption/tomography paired with transport	Disorder alone is not a pore network

**Table 6:** Common descriptors and chemistry-specific controls for selected Li, Na, and hydrated Zn electrolytes. Entries summarize comparison requirements rather than equivalent microscopic mechanisms.

Descriptor	Common Requirement	Li and Na Systems	Hydrated Zn Systems
Physical state and inventory	Temperature; thickness; salt basis; mobile solvent; processing and test pressure	Separate dry, solvent-containing and IL-containing states; identify metal and counterion	Measure retained water and drying history; distinguish water content from water activity
Architecture and access	Apparent BET with sample/probe conditions; bottlenecks; connected conducting phase	Resolve polymer, framework, liquid and ceramic pathways separately	Resolve swollen aqueous network and pore blocking by precipitates; dry BET is insufficient
Speciation and carrier availability	Coordination; association; exchange kinetics; measurement timescale	Li^+^ and Na^+^ require separate coordination and hopping/desolvation evidence	Zn^2+^ hydration, anion complexes, hydrolysis and possible proton-related carriers require separate assignments
Conductivity and transference	Total conductivity is not a cation-specific pathway; report electrode, bias, T and analysis method	Check polarization assumptions, interphase evolution and Li/Na reference convention	Check H^+^/OH^−^ and other ions; exclude corrosion/faradaic leakage from apparent Zn^2+^ transference
Interface and cycling	Current density plus half-cycle areal charge; metal inventory; contact; repeated cells	Li/Na deposition, stripping voids and chemistry-specific SEI; CCD depends on protocol	Hydrogen evolution, corrosion, pH drift and hydroxysalt precipitation alongside deposition
Relaxation and memory	WWRE with defined field-off wait, weak read and reversible reset	Exclude concentration polarization, contact change and irreversible interphase growth	Additionally exclude water redistribution, gas, pH change and dissolution/precipitation; persistence alone is insufficient

**Table 7:** Battery-specific tests that connect architecture to performance.

Battery Context	Relevant Pore Function	Cell-Level Test	Alternative Explanation
Li metal	Flux distribution and contact retention	Pressure, areal/cumulative capacity, interface imaging	Interphase chemistry, voids, filaments
Anode-free Li	Controlled nucleation with low inactive Li	Cathode loading and zero-excess Li accounting	SEI consumption and trapped Li
Na metal/Na-ion	Directional transport and interface integrity	Wetting, post-cycle cracks, through-plane σ	Contact loss or fracture
Zn quasi-solid	Ion/water regulation and uniform plating	Water activity, corrosion, H_2_, morphology	Gel chemistry or pH effect

**Table 8:** Operational evidence requirements for temporal ionic claims. Each claim applies only to the specimen, geometry, and protocol tested; missing controls remain unverified.

Claim	Minimum Discriminating Evidence	Alternatives to Test	Permitted Interpretation
Dynamic ionic gating	Bias-dependent conductance; recovery after removing the stimulus	Heating, electronic leakage, electrode reactions	Field-controlled response; retention is not established
History-dependent transport	Matched read conditions after different histories; resolved relaxation and drift	Dielectric/electrode polarization, concentration gradients, changing contacts	Prior excitation affects transport; memory remains unproven
Persistent reversible ionic state	State separation after a defined field-off wait; reversible recovery; independent ionic marker where feasible	Irreversible chemistry, solvent/water redistribution, structural or mechanical change	A retained reversible state in the tested system; bulk location requires separate evidence
Programmable ionic memory	Repeated WWRE; quantified retention, read disturbance, endurance and replicate variation; ionic attribution	Electrode-generated switching, metallic filaments, thermal and electronic contributions	Memory only within the validated geometry and operating window
Bulk battery-electrolyte attribution	Electrode-material and geometry/thickness controls; spatial or spectroscopic state evidence; relevant membrane conditions	Electrode/interphase-dominated signal or transfer from a distinct nanofluidic geometry	A bulk claim only when the controls locate the reversible ionic state within the electrolyte
Irreversible conditioning	Persistent shift without reversible reset under the stated protocol	Aging, corrosion, interphase growth, cracking or contact change	Conditioning or degradation; not programmable ionic memory

**Table 9:** Predictions and countertests for the six propositions in Figure 9.

Proposition	Observation to Test	Critical Countertest	What Remains Hypothetical
Carrier–bottleneck match	Ion-specific optimum under fixed chemistry	Vary carrier state at matched pore geometry	Generality across hosts
Dynamic coupling	Connectivity-dependent post-field relaxation	Isolated versus connected controls	A universal threshold
Finite useful window	Occupancy-dependent maximum with speciation	Disentangle aggregation and viscosity	Universal optimum position
Residence–time selectivity	Species-resolved exchange and mobility	Measure target cation and counterion separately	Transfer to full cells
Persistent bulk state	Field-off retention and geometry scaling	Electrode and sham-pulse tests	Bulk ionic-memory mechanism
Programmability	Repeatable write–wait–read–erase (WWRE) sequence	Read disturbance and irreversible chemistry	Practical battery benefit

**Table 10:** Minimum information required for mechanistic and memory claims.

Claim	Report at Minimum	Orthogonal Validation	Avoid Inferring
Accessible porosity	Operating-state access, size, thickness, direction	3D/network or infiltration evidence	BET area equals active path
Mobile carrier	Salt, solvent/water, temperature, speciation	Spectroscopy plus diffusion/transference	Conductivity equals free-ion density
Trapping kinetics	Target species, τ, protocol time window	Variable-T spectroscopy and recovery	Binding energy alone gives retention
Collective transport	Independent connectivity and relaxation descriptors	Matched structural series and held-out response	Nonlinear curve proves cooperation
Ionic memory	Write, field-off wait, weak read, erase, variance	Electrode, thickness, thermal, chemical controls	Any hysteresis is memory

## Data Availability

No new experimental data were created in this study. This review discusses and interprets published literature; the underlying data are available from the cited sources. Published experimental panels and molecular-dynamics results are reproduced or adapted from the sources identified in the figure captions. Original conceptual graphics organize the evidence and proposed tests; they are not measured or simulated material datasets.

## Abbreviations

Abbreviations	
AGG	aggregate.
BET	Brunauer-Emmett-Teller.
CCD	critical current density.
CEI	cathode-electrolyte interphase.
CIP	contact ion pair.
CNT	carbon nanotube.
COF	covalent organic framework.
DOI	digital object identifier.
DMF	N,N-dimethylformamide.
DLP	digital light processing.
DSC	differential scanning calorimetry.
ESW	electrochemical stability window.
GPE	gel polymer electrolyte.
HA	hyaluronic acid.
IR	infrared.
LATP	lithium aluminum titanium phosphate.
LiTFSI	lithium bis(trifluoromethanesulfonyl)imide.
LLZO	Li_7_La_3_Zr_2_O_12_ garnet electrolyte.
LLZTO	Ta-doped LLZO.
MOF	metal-organic framework.
NASICON	sodium superionic conductor.
NMR	nuclear magnetic resonance.
PA	polyamide.
PAF	porous aromatic framework.
PAN	polyacrylonitrile.
PC	propylene carbonate.
PEO	poly(ethylene oxide).
PEGDA	poly(ethylene glycol) diacrylate.
PFG-NMR	pulsed-field-gradient nuclear magnetic resonance.
PP	polypropylene.
PVDF	poly(vinylidene fluoride).
QSSE	quasi-solid-state electrolyte.
SEI	solid-electrolyte interphase.
SI	Supporting Information.
SSIP	solvent-separated ion pair.
TGA	thermogravimetric analysis.
TFSI	bis(trifluoromethanesulfonyl)imide.
WWRE	write–wait–read–erase; the field-off waiting interval tests retention.
XAS	X-ray absorption spectroscopy.
ZIF	zeolitic imidazolate framework.
Source-specific labels such as CC3, F-COF, P75, TpPa-COOLi, PVH, MOFLi, MSLi, ZIF-67-LA-PAM, PBA, and NMC811 are retained as used in the cited studies. An abbreviation is expanded only when its source provides an unambiguous definition.	
Symbols	
i	Species index; j is a generic species index.
${\tilde{\mu }}_{i}$	Molar electrochemical potential (J mol^−1^).
*μ* *_i_* ^∘^	Reference chemical contribution (J mol^−1^).
R	Gas constant (J mol^−1^ K^−1^).
T	Absolute temperature (K).
*a* *_i_*	Dimensionless ionic activity under a stated standard-state convention; a_j denotes a generic species activity.
*z* *_i_*	Signed charge number of species i (dimensionless).
F	Faraday constant (C mol^−1^).
ϕ	Electric potential (V).
*Ω* *_i_*	Molar stress-coupling volume (m^3^ mol^−1^).
*σ*_h_	Hydrostatic stress (Pa), compression positive in Equation (1).
Δ*G* *_i_*_,wall_	Effective pore-wall molar free-energy contribution (J mol^−1^).
Δ*G* *_i_*_,conf_	Effective confinement molar free-energy contribution (J mol^−1^).
*J* *_i_*	Molar flux in the stated reference frame (mol m^−2^ s^−1^).
*D* *_i_*	Kinetic diffusivity in the diagonal-mobility approximation (m^2^ s^−1^).
*c* *_i_*	Molar mobile-species concentration (mol m^−3^).
∇	Spatial gradient operator (m^−1^ when acting on a scalar).
ε	Absolute permittivity (F m^−1^).
*ρ*_f_	Fixed structural charge density (C m^−3^).
*ρ*_trap_	Separately counted trapped/slow ionic charge density (C m^−3^).
*σ*_eff_	Macroscopic effective ionic conductivity (S m^−1^).
*σ*_local_	Conductivity of the matched local conducting phase (S m^−1^).
*G*_arch_	Aggregate directional architecture factor; correlated geometric effects are included once (dimensionless).
M	Target cation Li^+^, Na^+^, or Zn^2+^; A denotes the monovalent counterion in Equation (6).
*σ*_M,screen_	Operational target-cation screening quantity defined by Equation (5) (S m^−1^).
*σ*_tot_	Measured total ionic conductivity (S m^−1^).
*t*_M_	Target-cation transference number; report method and reference frame (dimensionless).
z	Positive cation valence for MA_z ⇌ Mᶻ^+^ + zA^−^ (dimensionless).
*K*_d,pore_	Pore-phase dissociation equilibrium constant for the specified reaction and standard states (dimensionless).
*a*_M_	Activity of the target cation (dimensionless).
*a*_A_	Activity of the monovalent counterion (dimensionless).
*a*_MA*z*_	Activity of the undissociated salt species (dimensionless).
*k*_off,*i*_	Effective first-order detrapping/release rate (s^−1^).
*ν* *_i_*	Attempt-frequency prefactor in the release-rate ansatz (s^−1^).
Δ*G*_rel,*i*_^‡^	Release activation free energy per ion (J), not molar free energy.
*k*_B_	Boltzmann constant (J K^−1^); use a consistent per-ion energy convention in Equation (7).
*τ*_res,*i*_	Mean residence time in the stated escape model (s).
φ	Candidate conducting-phase volume fraction (dimensionless).
*φ*_c_	Geometric percolation threshold in that fraction (dimensionless).
*m*_perc_	Percolation conductivity-scaling exponent (dimensionless).
P	Structural/physical-state descriptor set; entries are not necessarily independent.
S(t)	Time-dependent internal ionic-state vector; components have their own units.
*d*_b_	Limiting ion-accessible bottleneck size (m).
*ε*_acc_	Accessible conducting-phase volume fraction (dimensionless).
*q*_wall_	Pore-wall surface-charge density under a specified convention (C m^−2^).
*φ*_mob_	Mobile-phase volume fraction (dimensionless).
*c*_free_	Operational free-carrier concentration (mol m^−3^).
*χ*_spec_	Vector of ionic-speciation fractions (dimensionless).
*θ*_pore_	Pore occupancy (dimensionless).
*θ*_trap_	Trap occupancy (dimensionless).
*ρ*_sc_	Net space-charge density (C m^−3^).
σ(t)	Time-dependent ionic conductivity under the specified measurement protocol (S m^−1^).
ℱ	Generic response functional in Equation (10), distinct from F.
E	Applied electric field (V m^−1^).
ℐ	Ion-chemistry/composition label; not electric current.
t	Time (s).
*Λ* *_i_*	Carrier–bottleneck ratio (dimensionless).
*d*_eff,*i*_	Effective transported-species diameter under the specified conditions (m).
*C*_dyn_	Proposed dynamic-coupling descriptor (dimensionless).
*τ*_relax_	Decay time of the specified local ionic perturbation (s).
*τ*_comm_	Communication time to a specified neighboring region (s).
*z*_M_	Target-cation charge number (dimensionless).
*c*_M,ava*i*l_	Available target-cation concentration in the local conducting phase (mol m^−3^).
*c*_M,total_	Total target-cation concentration on the same phase-volume basis (mol m^−3^).
*f*_M,ava*i*l_	Operational available fraction (dimensionless; 0–1).
*u*_M_	Positive electrical drift mobility of the target cation (m^2^ V^−1^ s^−1^).
*K* *_i_*	Proposed kinetic trapping ratio for a specified ionic species and observation window (dimensionless).
*τ*_op_	Selected operational/observation/stimulation time (s).
dS/dt	Time derivative of S; each component has its state-variable units per s.
𝒢	Generic state-evolution operator in Equation (15).
*τ*_read_	Duration of a minimally disturbing read (s).
*τ*_state_	Characteristic written-state lifetime (s).
*τ* *_i_*_rr_	Characteristic irreversible-change time (s).

## References

[1] Lou S., Zhang F., Fu C., Chen M., Ma Y., Yin G., Wang J. (2021). Interface Issues and Challenges in All-Solid-State Batteries: Lithium, Sodium, and Beyond. Adv. Mater..

[2] Xia W., Zhao Y., Zhao F., Adair K., Zhao R., Li S., Zou R., Zhao Y., Sun X. (2022). Antiperovskite Electrolytes for Solid-State Batteries. Chem. Rev..

[3] Yu S., Kim K., Wood B.C., Jung H.G., Chung K.Y. (2022). Structural design strategies for superionic sodium halide solid electrolytes. J. Mater. Chem. A.

[4] Li Y., Li M., Sun Z., Ni Q., Jin H., Zhao Y. (2023). Recent advance on NASICON electrolyte in solid-state sodium metal batteries. Energy Storage Mater..

[5] Gao X., Xing Z., Wang M., Nie C., Shang Z., Bai Z., Dou S.X., Wang N. (2023). Comprehensive insights into solid-state electrolytes and electrode-electrolyte interfaces in all-solid-state sodium-ion batteries. Energy Storage Mater..

[6] Yang Y., Yang S., Xue X., Zhang X., Li Q., Yao Y., Rui X., Pan H., Yu Y. (2024). Inorganic All-Solid-State Sodium Batteries: Electrolyte Designing and Interface Engineering. Adv. Mater..

[7] Huang L., Huang C. (2026). Inorganic Sodium Solid-State Electrolytes: Progress, Existing Issues, and Solutions Towards High-Performance All Solid-State Batteries. Electrochem. Energy Rev..

[8] Huang J., Li C., Jiang D., Gao J., Cheng L., Li G., Luo H., Xu Z.L., Shin D.M., Wang Y. (2025). Solid-State Electrolytes for Lithium Metal Batteries: State-of-the-Art and Perspectives. Adv. Funct. Mater..

[9] Henrique F., Żuk P.J., Gupta A. (2024). A network model to predict ionic transport in porous materials. Proc. Natl. Acad. Sci. USA.

[10] Wang X., Li X., Fan H., Ma L. (2022). Solid Electrolyte Interface in Zn-Based Battery Systems. Nano-Micro Lett..

[11] Xu T., Liu K., Sheng N., Zhang M., Liu W., Liu H., Dai L., Zhang X., Si C., Du H. (2022). Biopolymer-based hydrogel electrolytes for advanced energy storage/conversion devices: Properties, applications, and perspectives. Energy Storage Mater..

[12] Li X., Wang D., Ran F. (2023). Key approaches and challenges in fabricating advanced flexible zinc-ion batteries with functional hydrogel electrolytes. Energy Storage Mater..

[13] Niu B., Wang J., Guo Y., Li Z., Yuan C., Ju A., Wang X. (2024). Polymers for Aqueous Zinc-Ion Batteries: From Fundamental to Applications Across Core Components. Adv. Energy Mater..

[14] Li J., Azizi A., Zhou S., Liu S., Han C., Chang Z., Pan A., Cao G. (2025). Hydrogel polymer electrolytes toward better zinc-ion batteries: A comprehensive review. eScience.

[15] Peng H., Wang D., Zhang F., Yang L., Jiang X., Zhang K., Qian Z., Yang J. (2024). Improvements and Challenges of Hydrogel Polymer Electrolytes for Advanced Zinc Anodes in Aqueous Zinc-Ion Batteries. ACS Nano.

[16] Ge H., Xie X., Xie X., Zhang B., Li S., Liang S., Lu B., Zhou J. (2024). Critical challenges and solutions: Quasi-solid-state electrolytes for zinc-based batteries. Energy Environ. Sci..

[17] Xu Y., Zhang S., Liang T., Yao Z., Wang X., Gu C., Xia X., Tu J. (2021). Porous Polyamide Skeleton-Reinforced Solid-State Electrolyte: Enhanced Flexibility, Safety, and Electrochemical Performance. ACS Appl. Mater. Interfaces.

[18] Xu Y., Gao L., Wu X., Zhang S., Wang X., Gu C., Xia X., Kong X., Tu J. (2021). Porous Composite Gel Polymer Electrolyte with Interfacial Transport Pathways for Flexible Quasi Solid Lithium-Ion Batteries. ACS Appl. Mater. Interfaces.

[19] Li L., Jia S., Yue S., Wang C., Qiu H., Ji Y., Cao M., Zhang D. (2024). Hydrogel-stabilized zinc ion batteries: Progress and outlook. Green Chem..

[20] Wang Y., Jia Y., Li C., Cui H., Zhang R., Hong H., Li Q., Wang D., Zhi C. (2025). Progress in Developing Polymer Electrolytes for Advanced Zn Batteries. Small Methods.

[21] Yang J., Weng C., Sun P., Yin Y., Xu M., Pan L., Li J. (2025). Comprehensive regulation strategies for gel electrolytes in aqueous zinc-ion batteries. Coord. Chem. Rev..

[22] Zhang F., Liao T., Zhou Q., Bai J., Li X., Sun Z. (2025). Advancements in ion regulation strategies for enhancing the performance of aqueous Zn-ion batteries. Mater. Sci. Eng. R Rep..

[23] Jo Y., Jin D., Lim M., Lee H., An H., Seo J., Kim G., Ren X., Lee Y.M., Lee H. (2023). Structural and Chemical Evolutions of Li/Electrolyte Interfaces in Li-Metal Batteries: Tracing Compositional Changes of Electrolytes under Practical Conditions. Adv. Sci..

[24] Yoon G., Kim S., Kim J.S. (2023). Design Strategies for Anodes and Interfaces Toward Practical Solid-State Li-Metal Batteries. Adv. Sci..

[25] Li D., Liu H., Wang C., Yan C., Zhang Q., Nan C.W., Fan L.Z. (2024). High Ionic Conductive, Mechanical Robust Sulfide Solid Electrolyte Films and Interface Design for All-Solid-State Lithium Metal Batteries. Adv. Funct. Mater..

[26] Lin C., Ruan H., Wang M.S. (2025). Comprehensive Study of Li Deposition and Solid Electrolyte Cracking by Integrating Simulation and Experimental Data. Adv. Sci..

[27] Buragohain M., Kunnikuruvan S. (2025). Unveiling the Role of Ion Pairs in Determining Electrolyte Stability and Solid Electrolyte Interphase Formation in Lithium Metal Batteries. ACS Appl. Energy Mater..

[28] Li G., Zhao Z., Zhang S., Sun L., Li M., Yuwono J.A., Mao J., Hao J., Vongsvivut J., Xing L. (2023). A biocompatible electrolyte enables highly reversible Zn anode for zinc ion battery. Nat. Commun..

[29] Tang W., Qi R., Wu J., Zuo Y., Shi Y., Liu R., Yan W., Zhang J. (2024). Engineering, Understanding, and Optimizing Electrolyte/Anode Interfaces for All-Solid-State Sodium Batteries. Electrochem. Energy Rev..

[30] Robin P., Emmerich T., Ismail A., Niguès A., You Y., Nam G.H., Keerthi A., Siria A., Geim A.K., Radha B. (2023). Long-term memory and synapse-like dynamics in two-dimensional nanofluidic channels. Science.

[31] Kamsma T.M., Kim J., Kim K., Boon W.Q., Spitoni C., Park J., van Roij R. (2024). Brain-inspired computing with fluidic iontronic nanochannels. Proc. Natl. Acad. Sci. USA.

[32] Ismail A., Nam G.H., Lokhandwala A., Pandey S.V., Saurav K.V., You Y., Jyothilal H., Goutham S., Sajja R., Keerthi A. (2025). Programmable memristors with two-dimensional nanofluidic channels. Nat. Commun..

[33] Zhang H., Gan X., Yan Y., Zhou J. (2024). A Sustainable Dual Cross-Linked Cellulose Hydrogel Electrolyte for High-Performance Zinc-Metal Batteries. Nano-Micro Lett..

[34] Nguyen M.H., Ngo N.M., Kim B.K., Park S. (2024). Dual Ionic Pathways in Semi-Solid Electrolyte based on Binary Metal–Organic Frameworks Enable Stable Operation of Li-Metal Batteries at Extremely High Temperatures. Adv. Sci..

[35] Chen Z., Zhao W., Liu Q., Xu Y., Wang Q., Lin J., Wu H.B. (2024). Janus Quasi-Solid Electrolyte Membranes with Asymmetric Porous Structure for High-Performance Lithium-Metal Batteries. Nano-Micro Lett..

[36] Li Z., Oh K.S., Seo J.M., Qin W., Lee S., Zhai L., Li C., Baek J.B., Lee S.Y. (2024). A Solvent-Free Covalent Organic Framework Single-Ion Conductor Based on Ion–Dipole Interaction for All-Solid-State Lithium Organic Batteries. Nano-Micro Lett..

[37] Qin W.M., Li Z., Su W.X., Hu J.M., Zou H., Wu Z., Ruan Z., Cai Y.P., Li K., Zheng Q. (2025). Porous Organic Cage-Based Quasi-Solid-State Electrolyte with Cavity-Induced Anion-Trapping Effect for Long-Life Lithium Metal Batteries. Nano-Micro Lett..

[38] Zhao G., Ma H., Zhang C., Yang Y., Yu S., Zhu H., Sun Y., Guo H. (2025). Constructing Donor–Acceptor-Linked COFs Electrolytes to Regulate Electron Density and Accelerate the Li+ Migration in Quasi-Solid-State Battery. Nano-Micro Lett..

[39] Zhang H., Dubey R., Inniger M., Okur F., Wullich R., Parrilli A., Karabay D.T., Neels A., Kravchyk K.V., Kovalenko M.V. (2023). Ultrafast-sintered self-standing LLZO membranes for high energy density lithium-garnet solid-state batteries. Cell Rep. Phys. Sci..

[40] Wang S., Demopoulos G.P. (2024). High-conductive polymer-in-porous garnet solid electrolyte structure for all-solid-state lithium batteries enabled by molecular engineering. Energy Storage Mater..

[41] Su H., Zhong Y., Wang C., Liu Y., Hu Y., Li J., Wang M., Jiao L., Zhou N., Xiao B. (2024). Deciphering the critical role of interstitial volume in glassy sulfide superionic conductors. Nat. Commun..

[42] Lin X., Zhao Y., Wang C., Luo J., Fu J., Xiao B., Gao Y., Li W., Zhang S., Xu J. (2024). A Dual Anion Chemistry-Based Superionic Glass Enabling Long-Cycling All-Solid-State Sodium-Ion Batteries. Angew. Chem. Int. Ed..

[43] Porzio J., Scown C.D. (2021). Life-Cycle Assessment Considerations for Batteries and Battery Materials. Adv. Energy Mater..

[44] Fong K.D., Sumić B., O’Neill N., Schran C., Grey C.P., Michaelides A. (2024). The Interplay of Solvation and Polarization Effects on Ion Pairing in Nanoconfined Electrolytes. Nano Lett..

[45] Yan Y., Liu Z., Wan T., Li W., Qiu Z., Chi C., Huangfu C., Wang G., Qi B., Yan Y. (2023). Bioinspired design of Na-ion conduction channels in covalent organic frameworks for quasi-solid-state sodium batteries. Nat. Commun..

[46] Chen B.R., Monson A.G., Barnes P.L., Efaw C.M., Chuirazzi W.C., Woods M.E., Rollins H.W., Rufner J.F., Preston A.S., Guillen D.P. (2025). Digital light processing of porous LLZTO scaffolds for Li-garnet solid-state batteries. Cell Rep. Phys. Sci..

[47] Li Z., Myers S.K., Xiao J., Li Y., Noy N., Leuski A., Noy A. (2025). Neuromorphic ionic computing in droplet interface synapses. Sci. Adv..

[48] Kang S.H., Choi J., Kim J.Y., Shin D.O., Lee Y.G., Lee J. (2023). Mechanically Robust Ultrathin Solid Electrolyte Membranes Using a Porous Net Template for All-Solid-State Batteries. ACS Appl. Mater. Interfaces.

[49] Qi Y., Yan M., Qin S., Huang J., Huang X., Ren Y. (2025). Solid polymer electrolyte supported by an asymmetric porous polymer membrane for thermally stable and high-energy lithium metal batteries. J. Power Sources.

[50] Zhang G., Hu G., Li J., Feng G., Cui Y., Chen Z., Liu Z., Cui Y. (2025). Design strategies for ion-sieving charge mosaic membranes toward sustainable lithium extraction. Proc. Natl. Acad. Sci. USA.

[51] Chen L., Xue P., Liang Q., Liu X., Tang J., Li J., Liu J., Tang M., Wang Z. (2022). A Single-Ion Polymer Composite Electrolyte Via In Situ Polymerization of Electrolyte Monomers into a Porous MOF-Based Fibrous Membrane for Lithium Metal Batteries. ACS Appl. Energy Mater..

[52] Lu G., Wei H., Shen C., Zhou F., Zhang M., Chen Y., Jin H., Li J., Chen G., Wang J. (2022). Bifunctional MOF Doped PEO Composite Electrolyte for Long-Life Cycle Solid Lithium Ion Battery. ACS Appl. Mater. Interfaces.

[53] Mu A.U., Singh V.V., Kim H., Lee D.J., Kim N., Ruff C.X., Levy A., Young T.A., Paesani F., Cohen S.M. (2025). Structure–Activity Relationships in Ether-Functionalized Solid-State Metal–Organic Framework Electrolytes. Chem. Mater..

[54] Cheng P., Liu S., Jia X., Jiang Y., Zhang X. (2025). Robust MOF-Based Composite Solid-State Electrolyte Membrane for High-Performance Lithium–Metal Batteries. Nano Lett..

[55] Zhang J., Li J., Zhao G., Wang Q., Guo Y.G., Yang C. (2025). Mining Solid-State Electrolytes from Metal–Organic Framework Databases through Large Language Models and Representation Clustering. J. Am. Chem. Soc..

[56] Deng S., Yu H., Shang X., Hao Q., Tian F., Ge Z., Li Z. (2026). A Self-Crosslinking MOF-Based Electrolyte Enabling Stable and Selective Li + Conduction in Solid-State Lithium Batteries. Adv. Funct. Mater..

[57] Zhang Y., Wu W., Zhang X., Yang Z., Kou W., Shi L., Liu Y., Zhou S., Wang C., Ren Q. (2026). Metal-Organic Framework Electrolytes for Sub-−60°C Solid-State Lithium Batteries. Adv. Mater..

[58] Jiang J., Zhang R., Guo J., Zhang S., Min X., Liu Z., Liu N., Cao D., Xu J., Cheng P. (2025). Defect-Modulated MOF Nanochannels for the Quasi-Solid-State Electrolyte of a Dendrite-Free Lithium Metal Battery. Nano Lett..

[59] Han D., Zhao Z., Xu Z., Wang H., He Z., Wang H., Shi J., Zheng L. (2022). Metal Organic Frameworks Enabled Multifunctional Poly(ethylene oxide)-Based Solid Polymer Electrolytes with High Lithium-Ion Conductivity and Excellent Stability. ACS Appl. Energy Mater..

[60] Fan W., Huang Y., Yu M., She K., Gou J., Zhang Z. (2024). Designing metal-organic framework fiber network reinforced polymer electrolytes to provide continuous ion transport in solid state lithium metal batteries. Nano Res..

[61] Li Z., Wang L., Yu M., Liu Y., Liu B., Sun Z., Hu W., Zhu G. (2022). Lithium-Rich Porous Aromatic Framework-Based Quasi-Solid Polymer Electrolyte for High-Performance Lithium Ion Batteries. ACS Appl. Mater. Interfaces.

[62] Dong P., Zhang X., Hiscox W., Liu J., Zamora J., Li X., Su M., Zhang Q., Guo X., McCloy J. (2023). Toward High-Performance Metal–Organic-Framework-Based Quasi-Solid-State Electrolytes: Tunable Structures and Electrochemical Properties. Adv. Mater..

[63] Wang R., Dong Q., Wang C., Hong M., Gao J., Xie H., Guo M., Ping W., Wang X., He S. (2021). High-Temperature Ultrafast Sintering: Exploiting a New Kinetic Region to Fabricate Porous Solid-State Electrolyte Scaffolds. Adv. Mater..

[64] Li M., Kolek M., Frerichs J.E., Sun W., Hou X., Hansen M.R., Winter M., Bieker P. (2021). Investigation of Polymer/Ceramic Composite Solid Electrolyte System: The Case of PEO/LGPS Composite Electrolytes. ACS Sustain. Chem. Eng..

[65] Feng X., Fang H., Liu P., Wu N., Self E.C., Yin L., Wang P., Li X., Jena P., Nanda J. (2021). Heavily Tungsten-Doped Sodium Thioantimonate Solid-State Electrolytes with Exceptionally Low Activation Energy for Ionic Diffusion. Angew. Chem. Int. Ed..

[66] Liu G., Sun X., Yu X., Weng W., Yang J., Zhou D., Xiao R., Chen L., Yao X. (2021). Na10SnSb2S12: A nanosized air-stable solid electrolyte for all-solid-state sodium batteries. Chem. Eng. J..

[67] Weng W., Liu G., Shen L., Yao X. (2021). High ionic conductivity and stable phase Na11.5Sn2Sb0.5Ti0.5S12 for all-solid-state sodium batteries. J. Power Sources.

[68] Shen L., Yang J., Liu G., Avdeev M., Yao X. (2021). High ionic conductivity and dendrite-resistant NASICON solid electrolyte for all-solid-state sodium batteries. Mater. Today Energy.

[69] Weng W., Wan H., Liu G., Wu L., Wu J., Yao X. (2021). Liquid-Phase Synthesis of Nanosized Na11Sn2PS12 Solid Electrolytes for Room Temperature All-Solid-State Sodium Batteries. ACS Appl. Energy Mater..

[70] Kalnaus S., Amin R., Parish C., Parejiya A., Essehli R., Westover A., Tsai W.Y., Nanda J., Belharouak I. (2021). Effect of Composition on Mechanical Properties and Conductivity of the Dual-Ion Conductor Na_1+x_Mn_x/2_Zr_2−x/2_(PO_4_)_3_ for Solid-State Batteries. ACS Appl. Energy Mater..

[71] Kwak H., Lyoo J., Park J., Han Y., Asakura R., Remhof A., Battaglia C., Kim H., Hong S.T., Jung Y.S. (2021). Na2ZrCl6 enabling highly stable 3 V all-solid-state Na-ion batteries. Energy Storage Mater..

[72] Park D., Kim K., Chun G.H., Wood B.C., Shim J.H., Yu S. (2021). Materials design of sodium chloride solid electrolytes Na 3 MCl 6 for all-solid-state sodium-ion batteries. J. Mater. Chem. A.

[73] Huang H., Wu H.H., Chi C., Yang Y., Zheng J., Huang B., Wang S. (2021). Phase-structure-dependent Na ion transport in yttrium-iodide sodium superionic conductor Na_3_YI_6_. J. Mater. Chem. A.

[74] Tsuji F., Nasu A., Sakuda A., Tatsumisago M., Hayashi A. (2021). Mechanochemical synthesis and characterization of Na3–P1–W S4 solid electrolytes. J. Power Sources.

[75] Chi X., Zhang Y., Hao F., Kmiec S., Dong H., Xu R., Zhao K., Ai Q., Terlier T., Wang L. (2022). An electrochemically stable homogeneous glassy electrolyte formed at room temperature for all-solid-state sodium batteries. Nat. Commun..

[76] Prakash P., Fall B., Aguirre J., Sonnenberg L.A., Chinnam P.R., Chereddy S., Dikin D.A., Venkatnathan A., Wunder S.L., Zdilla M.J. (2023). A soft co-crystalline solid electrolyte for lithium-ion batteries. Nat. Mater..

[77] Ihrig M., Dashjav E., Laptev A.M., Ye R., Grüner D., Ziegner M., Odenwald P., Finsterbusch M., Tietz F., Fattakhova-Rohlfing D. (2022). Increasing the performance of all-solid-state Li batteries by infiltration of Li-ion conducting polymer into LFP-LATP composite cathode. J. Power Sources.

[78] Yu T.H., Huang C.Y., Wu M.C., Chen Y.J., Lan T., Tsai C.L., Chang J.K., Eichel R.A., Wu W.W. (2021). Atomic-scale investigation of Na_3_V_2_(PO_4_)_3_ formation process in chemical infiltration via in situ transmission electron microscope for solid-state sodium batteries. Nano Energy.

[79] Zhou R., Fan Y., Yan B., Chen H., Zhang B., Hwang J.Y., Zhou D., Fan L.Z. (2025). Novel Sodium-Rare-Earth-Silicate-Based Solid Electrolytes for All-Solid-State Sodium Batteries: Structure, Synthesis, Conductivity, and Interface. Adv. Energy Mater..

[80] Gao J.L., Zhao X.L., Ni X.Q., Mo Y.H., Tong Y.B., Cao D.Q., Luo H.B., Qiao-Qiao, Ren X.M. (2024). Multicomponent Quasi-Solid-State Polymer Electrolyte Incorporating MOFs and Halloysite Nanotubes for Enhanced Sodium Metal Battery Performance. ACS Appl. Energy Mater..

[81] Lin D., Lin Y., Pan R., Li J., Zhu A., Zhang T., Liu K., Feng D., Liu K., Zhou Y. (2025). Water-Restrained Hydrogel Electrolytes with Repulsion-Driven Cationic Express Pathways for Durable Zinc-Ion Batteries. Nano-Micro Lett..

[82] Grissa R., Seidl L., Dachraoui W., Sauter U., Battaglia C. (2022). Li7La3Zr2O12 Protonation as a Means to Generate Porous/Dense/Porous-Structured Electrolytes for All-Solid-State Lithium-Metal Batteries. ACS Appl. Mater. Interfaces.

[83] Kong O., Jiang G., Wang K., Yang A., Zheng J., Wu S., Han W., Li J., Li L., An X. (2025). Elucidation of Li+ Conduction Behavior in MOF Glass Electrolyte Toward Long-Cycling and High C-Rate Lithium Metal Batteries. Adv. Energy Mater..

[84] Zhang H., Okur F., Pant B., Klimpel M., Butenko S., Karabay D.T., Parrilli A., Neels A., Cao Y., Kravchyk K.V. (2024). Garnet-Based Solid-State Li Batteries with High-Surface-Area Porous LLZO Membranes. ACS Appl. Mater. Interfaces.

[85] Lu S., He K., Zhou L., Xu W., Lin X., Chen C., Lin Y., He J., Xu Y., Tian L. (2025). The Regulation of Ion Transport Microenvironment in Micropores to Precisely Construct Porous Polymer Electrolytes for Solid-State Lithium–Metal Batteries. ACS Nano.

[86] Yan K., Fan Y., Hu F., Li G., Yang X., Wang X., Li X., Peng C., Wang W., Fan H. (2024). A “Polymer-in-Salt” Solid Electrolyte Enabled by Fast Phase Transition Route for Stable Zn Batteries. Adv. Funct. Mater..

[87] Wang H., Song J., Zhang K., Fang Q., Zuo Y., Yang T., Yang Y., Gao C., Wang X., Pang Q. (2022). A strongly complexed solid polymer electrolyte enables a stable solid state high-voltage lithium metal battery. Energy Environ. Sci..

[88] An H., Li M., Liu Q., Song Y., Liu J., Yu Z., Liu X., Deng B., Wang J. (2024). Strong Lewis-acid coordinated PEO electrolyte achieves 4.8 V-class all-solid-state batteries over 580 Wh kg^−1^. Nat. Commun..

[89] Zhang W., Koverga V., Liu S., Zhou J., Wang J., Bai P., Tan S., Dandu N.K., Wang Z., Chen F. (2024). Single-phase local-high-concentration solid polymer electrolytes for lithium-metal batteries. Nat. Energy.

[90] Shao L., Tang J., Fang S., Zhang Y., Peng D., Huang J., Tang M., Wang Z. (2025). Regulation of the Ionic Transport and Electrochemical Stability in Poly(ethylene oxide) Solid Electrolyte via Terminal Polyfluorinated Modification. ACS Appl. Mater. Interfaces.

[91] Su Y., Rong X., Li H., Huang X., Chen L., Liu B., Hu Y.S. (2023). High-Entropy Microdomain Interlocking Polymer Electrolytes for Advanced All-Solid-State Battery Chemistries. Adv. Mater..

[92] Xu J., Ma C., Chang C., Lei X., Fu Y., Wang J., Liu X., Ding Y. (2021). Immobilizing Ceramic Electrolyte Particles into a Gel Matrix Formed In Situ for Stable Li-Metal Batteries. ACS Appl. Mater. Interfaces.

[93] Yu X., Liu Y., Goodenough J.B., Manthiram A. (2021). Rationally Designed PEGDA–LLZTO Composite Electrolyte for Solid-State Lithium Batteries. ACS Appl. Mater. Interfaces.

[94] Cheng A., Sun L., Menga N., Yang W., Zhang X. (2024). Interfacial performance evolution of ceramics-in-polymer composite electrolyte in solid-state lithium metal batteries. Int. J. Eng. Sci..

[95] Cheng M., Li D., Cao J., Sun T., Sun Q., Zhang W., Zha Z., Shi M., Zhang K., Tao Z. (2024). “Anions-in-Colloid” Hydrated Deep Eutectic Electrolyte for High Reversible Zinc Metal Anodes. Angew. Chem. Int. Ed..

[96] Zhou X., Huang S., Gao L., Zhang Z., Wang Q., Hu Z., Lin X., Li Y., Lin Z., Zhang Y. (2024). Molecular Bridging Induced Anti-Salting-Out Effect Enabling High Ionic Conductive ZnSO_4_-Based Hydrogel for Quasi-Solid-State Zinc Ion Batteries. Angew. Chem. Int. Ed..

[97] Sun M., Wang Z., Jiang J., Wang X., Yu C. (2024). Gelation mechanisms of gel polymer electrolytes for zinc-based batteries. Chin. Chem. Lett..

[98] Tan H., Meng C., Chen H., Yang J.L., Cao J.M., Wu X.L., Wang J.J. (2025). Breaking the Ice: Hofmeister Effect-Inspired Hydrogen Bond Network Reconstruction in Hydrogel Electrolytes for High-Performance Zinc-Ion Batteries. Small.

[99] Hoffknecht J.P., Wettstein A., Atik J., Krause C., Thienenkamp J., Brunklaus G., Winter M., Diddens D., Heuer A., Paillard E. (2023). Coordinating Anions “to the Rescue” of the Lithium Ion Mobility in Ternary Solid Polymer Electrolytes Plasticized With Ionic Liquids. Adv. Energy Mater..

[100] Lu Y.H., Liu Y.L. (2025). A Study on the Effects of Porous Structures of Polymer Matrixes on the Properties of Gel Polymer Electrolytes for Lithium Ion Batteries. ACS Appl. Energy Mater..

[101] Li J., Yu P., Zhang S., Wen Z., Wen Y., Zhou W., Dong X., Liu Y., Liang Y. (2021). Mild synthesis of superadhesive hydrogel electrolyte with low interfacial resistance and enhanced ionic conductivity for flexible zinc ion battery. J. Colloid Interface Sci..

[102] Ling W., Mo F., Wang J., Liu Q., Liu Y., Yang Q., Qiu Y., Huang Y. (2021). Self-healable hydrogel electrolyte for dendrite-free and self-healable zinc-based aqueous batteries. Mater. Today Phys..

[103] Zhang W., Guo F., Mi H., Wu Z.S., Ji C., Yang C., Qiu J. (2022). Kinetics-Boosted Effect Enabled by Zwitterionic Hydrogel Electrolyte for Highly Reversible Zinc Anode in Zinc-Ion Hybrid Micro-Supercapacitors. Adv. Energy Mater..

[104] Liu J., Long J., Shen Z., Jin X., Han T., Si T., Zhang H. (2021). A Self-Healing Flexible Quasi-Solid Zinc-Ion Battery Using All-In-One Electrodes. Adv. Sci..

[105] Chen M., Chen J., Zhou W., Han X., Yao Y., Wong C.P. (2021). Realizing an All-Round Hydrogel Electrolyte toward Environmentally Adaptive Dendrite-Free Aqueous Zn–MnO_2_ Batteries. Adv. Mater..

[106] Cao F., Wu B., Li T., Sun S., Jiao Y., Wu P. (2022). Mechanoadaptive morphing gel electrolyte enables flexible and fast-charging Zn-ion batteries with outstanding dendrite suppression performance. Nano Res..

[107] Fu C., Wang Y., Lu C., Zhou S., He Q., Hu Y., Feng M., Wan Y., Lin J., Zhang Y. (2022). Modulation of hydrogel electrolyte enabling stable zinc metal anode. Energy Storage Mater..

[108] Wu M., Zhang Y., Xu L., Yang C., Hong M., Cui M., Clifford B.C., He S., Jing S., Yao Y. (2022). A sustainable chitosan-zinc electrolyte for high-rate zinc-metal batteries. Matter.

[109] Xu L., Meng T., Zheng X., Li T., Brozena A.H., Mao Y., Zhang Q., Clifford B.C., Rao J., Hu L. (2023). Nanocellulose-Carboxymethylcellulose Electrolyte for Stable, High-Rate Zinc-Ion Batteries. Adv. Funct. Mater..

[110] Liu Q., Yu Z., Zhuang Q., Kim J.K., Kang F., Zhang B. (2023). Anti-Fatigue Hydrogel Electrolyte for All-Flexible Zn-Ion Batteries. Adv. Mater..

[111] Zhang H., Gan X., Song Z., Zhou J. (2023). Amphoteric Cellulose-Based Double-Network Hydrogel Electrolyte Toward Ultra-Stable Zn Anode. Angew. Chem. Int. Ed..

[112] Zeng W., Zhang S., Lan J., Lv Y., Zhu G., Huang H., Lv W., Zhu Y. (2024). Double Network Gel Electrolyte with High Ionic Conductivity and Mechanical Strength for Zinc-Ion Batteries. ACS Nano.

[113] Ge H., Qin L., Zhang B., Jiang L., Tang Y., Lu B., Tian S., Zhou J. (2024). An ionically cross-linked composite hydrogel electrolyte based on natural biomacromolecules for sustainable zinc-ion batteries. Nanoscale Horiz..

[114] Sun Y., Song L.N., Du X.Y., Liang S., Wang Y., Wang X.X., Ji G.J., Xu J.J. (2025). In Situ Copolymerizing ZrO_2_ with Hydrogel Electrolytes toward High-Rate and Long-Life Quasi-Solid-State Zn–Ion Batteries. Adv. Energy Mater..

[115] Shen Z., Zhong J., Jiang S., Xie W., Zhan S., Lin K., Zeng L., Hu H., Lin G., Lin Y. (2022). Polyacrylonitrile Porous Membrane-Based Gel Polymer Electrolyte by In Situ Free-Radical Polymerization for Stable Li Metal Batteries. ACS Appl. Mater. Interfaces.

[116] Liu J., Lin H., Li H., Zhao D., Liu W., Tao X. (2024). In Situ Polymerization Derived from PAN-Based Porous Membrane Realizing Double-Stabilized Interface and High Ionic Conductivity for Lithium-Metal Batteries. ACS Appl. Mater. Interfaces.

[117] Wichmann L., Tengen B., Yan P., Lennartz P., Cekic-Laskovic I., Winter M., Brunklaus G. (2026). Boosting the Energy Density of “Anode-Free” Lithium Metal Batteries via Electrospun Polymeric Scaffolds. Adv. Funct. Mater..

[118] Jia Z., Liu Y., Li H., Xiong Y., Miao Y., Liu Z., Ren F. (2024). In-situ polymerized PEO-based solid electrolytes contribute better Li metal batteries: Challenges, strategies, and perspectives. J. Energy Chem..

[119] Zou S., Yang Y., Wang J., Zhou X., Wan X., Zhu M., Liu J. (2024). In situ polymerization of solid-state polymer electrolytes for lithium metal batteries: A review. Energy Environ. Sci..

[120] Chiu S.K., Lu Y.H., Chen H.Y., Liu Y.L. (2024). Construction of Ion-Conducting Channels with Surface-Functionalized Carbon Nanotubes in Gel/Solid Polymer Electrolytes for Lithium-Ion Batteries. ACS Appl. Energy Mater..

[121] Lin R., Jin Y., Li Y., Fu M., Gong Y., Lei L., Zhang Y., Xu J., Xiong Y. (2025). Decoupling Interfacial Stability and Ion Transport in Solid Polymer Electrolyte by Tailored Ligand Chemistry for Lithium Metal Battery. Adv. Funct. Mater..

[122] Li Y., Yuan W., Hu Z., Shen Y., Wu G., Cong F., Fu X., Lu F., Li Y., Liu P. (2025). Constructing PVDF-Based Polymer Electrolyte for Lithium Metal Batteries by Polymer-Induced Phase Structure Adjustment Strategy. Adv. Funct. Mater..

[123] Jiang M., Ma Y., Bao C., Wang H., Zhang F., Zhang H., Wang L. (2026). Opportunities for Polymer Electrolytes in Sustaining High-Voltage Lithium Metal Batteries. Adv. Funct. Mater..

[124] Hou J., Zhang J., Wu S., Liu X., Li Y., Yan Z., Zhang K., Lu Y., Chen J. (2026). Advanced Design and Characterization of Polyether-Based Solid-State Electrolytes for High-Energy-Density Lithium Batteries. Adv. Mater..

[125] Chen X., Kong X. (2023). Nanoscale Confinement Effects on Ionic Conductivity of Solid Polymer Electrolytes: The Interplay between Diffusion and Dissociation. Nano Lett..

[126] Kim G.H., Jang J., Kang J. (2024). Enhanced Free Li-Ion Mobility in Solid-State Electrolytes via Long-Range Assembly of Porous Materials. ACS Appl. Mater. Interfaces.

[127] Li C., Deng S., Feng W., Cao Y., Bai J., Tian X., Dong Y., Xia F. (2023). A Universal Room-Temperature 3D Printing Approach Towards porous MOF Based Dendrites Inhibition Hybrid Solid-State Electrolytes. Small.

[128] Liu X., Liang Q., Chen L., Tang J., Liu J., Tang M., Wang Z. (2023). PEO-Based Solid-State Electrolytes Reinforced by High Strength, Interconnected MOF Networks. ACS Appl. Energy Mater..

[129] Wu Z., Yi Y., Hai F., Tian X., Zheng S., Guo J., Tang W., Hua W., Li M. (2023). A Metal–Organic Framework Based Quasi-Solid-State Electrolyte Enabling Continuous Ion Transport for High-Safety and High-Energy-Density Lithium Metal Batteries. ACS Appl. Mater. Interfaces.

[130] Hu A., Sun C., Li C., Sun Z., Fan J., Zheng M., Dong Q. (2023). A Novel Metal–Organic-Framework-Based Composite Solid Electrolyte for Lithium Metal Batteries. ACS Appl. Mater. Interfaces.

[131] Song Z., Li H., Zheng F., Lin H., Liu J., Liu W., Sun G., Tao X. (2023). In Situ Gelation of a 1,3-Dioxolane Dual-Permeable Porous Tandem Framework with Excellent Interfacial Stability to Power Long-Cycling Solid-State Lithium Metal Batteries. ACS Appl. Mater. Interfaces.

[132] Zheng S., Li Z., Chen L., Huang Y., Shi J., Wang S., Liu Y., Liu Y., Cai Y.P., Zheng Q. (2023). MOF-Based 3D Ion-Conducting Network Enables High-Voltage All-Solid-State Lithium Metal Batteries at Room Temperature. ACS Mater. Lett..

[133] Wang T., Yuan H., Wang H., Guo Y., Yang J., Liu X., Liu B., Wang X., Kirk C.H., Sun J. (2024). Unlocking Fast Ionic Transport in Sub-Nano Channels of MOF-Based Electrolytes for Next-Generation Batteries. Adv. Funct. Mater..

[134] Nguyen M.C., Nguyen H.L., Duong T.P.M., Kim S.H., Kim J.Y., Bae J.H., Kim H.K., Lim S.N., Ahn W. (2024). Highly Safe, Ultra-Thin MOF-Based Solid Polymer Electrolytes for Superior All-Solid-State Lithium-Metal Battery Performance. Adv. Funct. Mater..

[135] Duan S., Qian L., Zheng Y., Zhu Y., Liu X., Dong L., Yan W., Zhang J. (2024). Mechanisms of the Accelerated Li + Conduction in MOF-Based Solid-State Polymer Electrolytes for All-Solid-State Lithium Metal Batteries. Adv. Mater..

[136] Mu A.U., Cai G., Chen Z. (2024). Metal–Organic Frameworks for the Enhancement of Lithium-Based Batteries: A Mini Review on Emerging Functional Designs. Adv. Sci..

[137] Zhou J., Wang X., Fu J., Chen L., Wei X., Jia R., Shi L. (2024). A 3D Cross-Linked Metal-Organic Framework (MOF)-Derived Polymer Electrolyte for Dendrite-Free Solid-State Lithium-Ion Batteries. Small.

[138] Hong Z., Li P., Zou Q., Gu L., Wang J., Deng L., Wang C., Zhang Y., Li M., Chen J. (2024). Metal Organic Framework (MOF-808) Incorporated Composite Polymer Electrolyte for Stable All-Solid-State Lithium Batteries. ACS Appl. Energy Mater..

[139] Wei L., Xu X., Xi K., Shi X., Cheng X., Lei Y., Gao Y. (2024). Polydopamine-Induced Metal–Organic Framework Network-Enhanced High-Performance Composite Solid-State Electrolytes for Dendrite-Free Lithium Metal Batteries. ACS Appl. Mater. Interfaces.

[140] Li S., Pei F., Ding Y., Guo X., Zhang X., Tao H., He Z., Hu H., Zhang L. (2025). Metal-Organic Framework-Derived Elastic Solid Polymer Electrolytes Enabled by Covalent Crosslinking for High-Performance Lithium Metal Batteries. Adv. Funct. Mater..

[141] Feng L., Shen Z.Z., Yang Q., Zhang J., Chen S. (2025). MOF-Ionic Liquid Structured Polymer Electrolytes with Multi-Channel Ion Transport Pathways for Wide-Temperature Solid-State Lithium Batteries. Adv. Mater..

[142] Chen Z., Li P., Anderson R., Wang X., Zhang X., Robison L., Redfern L.R., Moribe S., Islamoglu T., Gómez-Gualdrón D.A. (2020). Balancing volumetric and gravimetric uptake in highly porous materials for clean energy. Science.

[143] Lee J.H., Lee H., Lee J., Kang T.W., Park J.H., Shin J.H., Lee H., Majhi D., Lee S.U., Kim J.H. (2023). Multicomponent Covalent Organic Framework Solid Electrolyte Allowing Effective Li-Ion Dissociation and Diffusion for All-Solid-State Batteries. ACS Nano.

[144] Shan Z., Wu M., Du Y., Xu B., He B., Wu X., Zhang G. (2021). Covalent Organic Framework-Based Electrolytes for Fast Li + Conduction and High-Temperature Solid-State Lithium-Ion Batteries. Chem. Mater..

[145] Kang T.W., Lee J.H., Lee J., Park J.H., Shin J.H., Ju J.M., Lee H., Lee S.U., Kim J.H. (2023). An Ion-Channel-Restructured Zwitterionic Covalent Organic Framework Solid Electrolyte for All-Solid-State Lithium-Metal Batteries. Adv. Mater..

[146] Zhao G., Mei Z., Duan L., An Q., Yang Y., Zhang C., Tan X., Guo H. (2023). COF-based single Li + solid electrolyte accelerates the ion diffusion and restrains dendrite growth in quasi-solid-state organic batteries. Carbon Energy.

[147] Zhang C., Zhao L., Li F., Song X., Chen J., Zeng J., Xi L., Hu R., Zhu M., Liu J. (2025). Optimizing the Electron Density of PVDF-HFP-Based Solid Polymer Electrolyte by Donor–Acceptor COF Toward High-Performance Solid-State Lithium Metal Batteries. Adv. Mater..

[148] Tian Y., Cheng X., Cheng L., Chang Y., Wu J., Gu M., Bang K.T., Wang R., Tao R., Wang Y. (2026). Single-Crystalline Borate Covalent Organic Frameworks for Solid-State Lithium Metal Batteries. Adv. Sci..

[149] Choi R.H., So J., Kim Y., Lee D., Byon H.R. (2024). Li+ Conduction of Soft-Base Anion-Immobilized Covalent Organic Frameworks for All-Solid-State Lithium–Metal Batteries. ACS Energy Lett..

[150] Li S., Wang L., Liu C., Liu Y., Li Z., Liu B., Sun Z., Hu W. (2024). Lithium-Rich Porous Aromatic Framework Doped Quasi-Solid Polymer Electrolyte for Lithium Battery with High Cycling Stability. ACS Appl. Mater. Interfaces.

[151] Li J.X., Guan D.H., Wang X.X., Miao C.L., Li J.Y., Xu J.J. (2024). Highly Stable Organic Molecular Porous Solid Electrolyte with One-Dimensional Ion Migration Channel for Solid-State Lithium−Oxygen Battery. Adv. Mater..

[152] Zhu Y., Zheng S., Lu P., Ma J., Das P., Su F., Cheng H.M., Wu Z.S. (2022). Kinetic regulation of MXene with water-in-LiCl electrolyte for high-voltage micro-supercapacitors. Natl. Sci. Rev..

[153] Chen M., Shi X., Wang X., Liu H., Wang S., Meng C., Liu Y., Zhang L., Zhu Y., Wu Z.S. (2022). Low-temperature and high-voltage planar micro-supercapacitors based on anti-freezing hybrid gel electrolyte. J. Energy Chem..

[154] Osterrieth J.W.M., Rampersad J., Madden D., Rampal N., Skoric L., Connolly B., Allendorf M.D., Stavila V., Snider J.L., Ameloot R. (2022). How Reproducible are Surface Areas Calculated from the BET Equation?. Adv. Mater..

[155] Islamoglu T., Idrees K.B., Son F.A., Chen Z., Lee S.J., Li P., Farha O.K. (2022). Are you using the right probe molecules for assessing the textural properties of metal–organic frameworks?. J. Mater. Chem. A.

[156] Meng J., Yin M., Guo K., Zhou X., Xue Z. (2025). In Situ Polymerization in COF Boosts Li-Ion Conduction in Solid Polymer Electrolytes for Li Metal Batteries. Nano-Micro Lett..

[157] Liu X.X., Pan L., Zhang H., Liu C., Cao M., Gao M., Zhang Y., Xu Z., Wang Y., Sun Z. (2025). Indium-MOF as Multifunctional Promoter to Remove Ionic Conductivity and Electrochemical Stability Constraints on Fluoropolymer Electrolytes for All-Solid-State Lithium Metal Battery. Nano-Micro Lett..

[158] Guan J., Feng X., Zeng Q., Li Z., Liu Y., Chen A., Wang H., Cui W., Liu W., Zhang L. (2023). A New In Situ Prepared MOF-Natural Polymer Composite Electrolyte for Solid Lithium Metal Batteries with Superior High-Rate Capability and Long-Term Cycling Stability at Ultrahigh Current Density. Adv. Sci..

[159] Qi X., Cai D., Wang X., Xia X., Gu C., Tu J. (2022). Ionic Liquid-Impregnated ZIF-8/Polypropylene Solid-like Electrolyte for Dendrite-free Lithium-Metal Batteries. ACS Appl. Mater. Interfaces.

[160] Kim K.J., Shin K.S., Choi J.H., Han J., Park M.S., Kim M.K., Lee D.K., Yi E.J., Kim J.H. (2025). Instantaneous Photonic Sintering Process for Scalable Fabrication of a 3D Garnet Electrolyte Scaffold for Solid-State Batteries. ACS Energy Lett..

[161] Tishkevich D.I., Vorobjova A.I., Bondaruk A.A., Dashkevich E.S., Shimanovich D.L., Razanau I.U., Zubar T.I., Yakimchuk D.V., Dong M.G., Sayyed M.I. (2022). The Interrelation of Synthesis Conditions and Wettability Properties of the Porous Anodic Alumina Membranes. Nanomaterials.

[162] Ge S., Wu J., Wang R., Zhang L., Liu S., Ma X., Fu K., Yan J., Yu J., Ding B. (2024). Tailoring Practical Solid Electrolyte Composites Containing Ferroelectric Ceramic Nanofibers and All-Trans Block Copolymers for All-Solid-State Lithium Metal Batteries. ACS Nano.

[163] Kim H.M., Subramanian Y., Ryu K.S. (2023). I-Rich Thioantimonate Argyrodite Glass-Ceramic Electrolyte with High Air Stability and Compatibility with Lithium. ACS Appl. Energy Mater..

[164] Zhang S., Zhao F., Chen J., Fu J., Luo J., Alahakoon S.H., Chang L.Y., Feng R., Shakouri M., Liang J. (2023). A family of oxychloride amorphous solid electrolytes for long-cycling all-solid-state lithium batteries. Nat. Commun..

[165] Zhou R., Luo K., Martin S.W., An Q. (2024). Insights into Lithium Sulfide Glass Electrolyte Structures and Ionic Conductivity via Machine Learning Force Field Simulations. ACS Appl. Mater. Interfaces.

[166] Hu Y., Fu J., Xu J., Luo J., Zhao F., Su H., Liu Y., Lin X., Li W., Kim J.T. (2024). Superionic amorphous NaTaCl6 halide electrolyte for highly reversible all-solid-state Na-ion batteries. Matter.

[167] Ridley P., Nguyen L.H.B., Sebti E., Han B., Duong G., Chen Y.T., Sayahpour B., Cronk A., Deysher G., Ham S.Y. (2024). Amorphous and nanocrystalline halide solid electrolytes with enhanced sodium-ion conductivity. Matter.

[168] Luo K., Zhou R., Martin S.W., An Q. (2024). Flexible doorway controlled Na + ion diffusion in NaPSO glassy electrolytes from machine-learning force field simulations. J. Mater. Chem. A.

[169] Huang H., Chi C., Zhang J., Zheng X., Wu Y., Shen J., Wang X., Wang S. (2022). Fast Ion Transport Mechanism and Electrochemical Stability of Trivalent Metal Iodide-based Na Superionic Conductors Na_3_XI_6_ (X = Sc, Y, La, and In). ACS Appl. Mater. Interfaces.

[170] Park J., Son J.P., Ko W., Kim J.S., Choi Y., Kim H., Kwak H., Seo D.H., Kim J., Jung Y.S. (2022). NaAlCl_4_: New Halide Solid Electrolyte for 3 V Stable Cost-Effective All-Solid-State Na-Ion Batteries. ACS Energy Lett..

[171] Fu J., Wang S., Liang J., Alahakoon S.H., Wu D., Luo J., Duan H., Zhang S., Zhao F., Li W. (2023). Superionic Conducting Halide Frameworks Enabled by Interface-Bonded Halides. J. Am. Chem. Soc..

[172] Lian Y., Wu M., Xu B., He B., Liu G., Shi J., Kuang Q., Wang H., Ouyang C. (2023). Phase-structure design for sodium chloride solid electrolytes with outstanding performance: A first-principles approach. J. Mater. Chem. A.

[173] Wang C., Wang S., Liu X., Wu Y., Yu R., Duan H., Kim J.T., Huang H., Wang J., Mo Y. (2023). New insights into aliovalent substituted halide solid electrolytes for cobalt-free all-solid state batteries. Energy Environ. Sci..

[174] Hu L., Li H., Chen F., Liu Y., Wang J., Ma C. (2024). Overcoming the Na-ion conductivity bottleneck for the cost-competitive chloride solid electrolytes. J. Energy Chem..

[175] Huang Z., Yoshida S., Akamatsu H., Hayashi K., Ohno S. (2024). Na*M*Cl_6_ (*M* = Nb and Ta): A New Class of Sodium-Conducting Halide-Based Solid Electrolytes. ACS Mater. Lett..

[176] Fu C., Li Y., Xu W., Feng X., Gu W., Liu J., Deng W., Wang W., Abeykoon A.M.M., Su L. (2024). LaCl_3_-based sodium halide solid electrolytes with high ionic conductivity for all-solid-state batteries. Nat. Commun..

[177] Barker K., McKinney S.L., Artal R., Jiménez R., Tapia-Ruiz N., Skinner S.J., Aguadero A., Seymour I.D. (2024). The importance of A-site cation chemistry in superionic halide solid electrolytes. Nat. Commun..

[178] Ouachouo L., Es-soufi H., Sayyed M.I., Ghyati S., Bih L. (2023). Investigation of structural, thermal, and electrical characteristics of zirconium-doped lithium-phosphate glasses. Heliyon.

[179] Ouachouo L., Es-soufi H., Sayyed M.I., Bih L. (2023). Effect of tungsten metal-oxide addition on physical, structural, and electrical properties of borophosphate glasses. Ceram. Int..

[180] Lee B.D., Gavali D.S., Kim H., Kim S., Cho M.Y., Pyo K., Lee Y.K., Park W.B., Sohn K.S. (2025). Discovering virtual Na-based argyrodites as solid-state electrolytes using DFT, AIMD, and machine learning techniques. J. Mater. Chem. A.

[181] Holmes S.E., Kondek J., Zhang P., Faka V., Newnham J.A., Gronych L.M., Hansen M.R., Zeier W.G., Cui Y. (2025). LiI-Modified Glass-Ceramic Lithium Thioborate: From Fundamentals to Applications in Solid-State Batteries. Chem. Mater..

[182] Yang J., Schaller M., Cherkashinin G., Zhang R., Indris S., Corte D.A.D., Kondrakov A., Brezesinski T., Strauss F. (2025). Glass-Ceramic Lithium Thiophosphate Electrolytes with Enhanced Conductivity and (Chemo)mechanical Properties for All-Solid-State Batteries. Chem. Mater..

[183] Chen Z., Du T., Krishnan N.M.A., Yue Y., Smedskjaer M.M. (2025). Disorder-induced enhancement of lithium-ion transport in solid-state electrolytes. Nat. Commun..

[184] Wang Q., Yu C., Li L., Liu X., Zhang X., Gao G., Wang Y., Li G. (2023). Sc-doping in Na_3_Zr_2_Si_2_PO_12_ electrolytes enables preeminent performance of solid-state sodium batteries in a wide temperature range. Energy Storage Mater..

[185] Lee J.E., Park K.H., Kim J.C., Wi T.U., Ha A.R., Song Y.B., Oh D.Y., Woo J., Kweon S.H., Yeom S.J. (2022). Universal Solution Synthesis of Sulfide Solid Electrolytes Using Alkahest for All-Solid-State Batteries. Adv. Mater..

[186] Liu S., Jiang G., Wang Y., Liu C., Zhang T., Wei Y., An B. (2024). Vitrified Metal–Organic Framework Composite Electrolyte Enabling Dendrite-Free and Long-Lifespan Solid-State Lithium Metal Batteries. ACS Nano.

[187] Jiang G., Qu C., Xu F., Zhang E., Lu Q., Cai X., Hausdorf S., Wang H., Kaskel S. (2021). Glassy Metal–Organic-Framework-Based Quasi-Solid-State Electrolyte for High-Performance Lithium-Metal Batteries. Adv. Funct. Mater..

[188] Rees G.J., Spencer Jolly D., Ning Z., Marrow T.J., Pavlovskaya G.E., Bruce P.G. (2021). Imaging Sodium Dendrite Growth in All-Solid-State Sodium Batteries Using ^23^Na T_2_-Weighted Magnetic Resonance Imaging. Angew. Chem. Int. Ed..

[189] Hill R., Peretti A., Small L.J., Spoerke E.D., Cheng Y.T. (2023). Molten Sodium Penetration in NaSICON Electrolytes at 0.1 A cm^−2^. ACS Appl. Energy Mater..

[190] Gao Z., Yang J., Li G., Ferber T., Feng J., Li Y., Fu H., Jaegermann W., Monroe C.W., Huang Y. (2022). TiO_2_ as Second Phase in Na_3_Zr_2_Si_2_PO_12_ to Suppress Dendrite Growth in Sodium Metal Solid-State Batteries. Adv. Energy Mater..

[191] Lu Y., Zhao C.Z., Yuan H., Cheng X.B., Huang J.Q., Zhang Q. (2021). Critical Current Density in Solid-State Lithium Metal Batteries: Mechanism, Influences, and Strategies. Adv. Funct. Mater..

[192] Sun J., Zhang S., Li J., Xie B., Ma J., Dong S., Cui G. (2023). Robust Transport: An Artificial Solid Electrolyte Interphase Design for Anode-Free Lithium-Metal Batteries. Adv. Mater..

[193] Song Y., Bhargava B., Stewart D.M., Talin A.A., Rubloff G.W., Albertus P. (2023). Electrochemical-mechanical coupling measurements. Joule.

[194] Quérel E., Williams N.J., Seymour I.D., Skinner S.J., Aguadero A. (2023). Operando Characterization and Theoretical Modeling of Metal|Electrolyte Interphase Growth Kinetics in Solid-State Batteries. Part I Experiments. Chem. Mater..

[195] Ono T., Hiraoka K., Kobayashi T., Yamamoto K., Seki S. (2023). Degradation Analysis of Oxide-Based All-Solid-State Na Batteries by Operando Raman Spectroscopy. ACS Appl. Energy Mater..

[196] Nuwayhid R.B., Kozen A.C., Long D.M., Ahuja K., Rubloff G.W., Gregorczyk K.E. (2023). Dynamic Electrode–Electrolyte Intermixing in Solid-State Sodium Nano-Batteries. ACS Appl. Mater. Interfaces.

[197] Geng L., Xue D., Yao J., Dai Q., Sun H., Zhu D., Rong Z., Fang R., Zhang X., Su Y. (2023). Morphodynamics of dendrite growth in alumina based all solid-state sodium metal batteries. Energy Environ. Sci..

[198] Xiang L., Jiang D., Gao Y., Zhang C., Ren X., Zhu L., Gao S., Zhan X. (2024). Self-Formed Fluorinated Interphase with Fe Valence Gradient for Dendrite-Free Solid-State Sodium-Metal Batteries. Adv. Funct. Mater..

[199] Gao Z., Bai Y., Feng J., Yang J., Liu P., Yuan H., Guan X., Wang F.R., Huang Y. (2024). Controlling Sodium Dendrite Growth via Grain Boundaries in Na_3_Zr_2_Si_2_PO_12_ Electrolyte. Adv. Energy Mater..

[200] Huang Z., Fan H., Jiang G., Xiong J., Yuan S. (2025). Facing the Ionic Conductivity of Solid Polymer Electrolytes for Sodium–Metal Batteries: A Mini Review. ACS Appl. Mater. Interfaces.

[201] Das S., Jana S., Orságh M., Byś K., Mishra J., Uchman M., Adyam V. (2023). Building Sodium Metal Battery with Polyisoprene-Based Air-Stable Single-Ion Gel Polymer Electrolyte. ACS Appl. Energy Mater..

[202] Guo J., Feng F., Jiang X., Wang R., Chu D., Ren Y., Chen F., He P., Ma Z.F., Chen S. (2024). Boosting Selective Na^+^ Migration Kinetics in Structuring Composite Polymer Electrolyte Realizes Ultrastable All-Solid-State Sodium Batteries. Adv. Funct. Mater..

[203] Vasudevan S., Dwivedi S., Morekonda Ganesh Babu K.B., Balaya P. (2024). Investigation of Solid Polymer Electrolytes for NASICON-Type Solid-State Symmetric Sodium-Ion Battery. ACS Appl. Mater. Interfaces.

[204] Wang X., Bai Y., Lv Z., Liu Y., Dong W., Bi H., Huang F. (2025). In Situ Polymerized Localized High-Concentration Electrolytes for Ultrahigh-Rate Sodium Metal Batteries. Adv. Energy Mater..

[205] Del Olmo R., Olmedo-Martínez J.L., Santino F., Villaluenga I. (2025). Fluorine-Free All-Solid-State Polymer Electrolytes for Sodium Metal Batteries. ACS Appl. Energy Mater..

[206] Ma Q., Ortmann T., Yang A., Sebold D., Burkhardt S., Rohnke M., Tietz F., Fattakhova-Rohlfing D., Janek J., Guillon O. (2022). Enhancing the Dendrite Tolerance of NaSICON Electrolytes by Suppressing Edge Growth of Na Electrode along Ceramic Surface. Adv. Energy Mater..

[207] Sun Z., Zhao Y., Ni Q., Liu Y., Sun C., Li J., Jin H. (2022). Solid-State Na Metal Batteries with Superior Cycling Stability Enabled by Ferroelectric Enhanced Na/Na_3_Zr_2_Si_2_PO_12_ Interface. Small.

[208] Yao Y., Wang X., Dong C., Chen J., Wang D., Mao Z. (2022). Constructing effective interface for room-temperature Beta-Al_2_O_3_ based sodium metal batteries. J. Power Sources.

[209] Sun Z., Li L., Sun C., Ni Q., Zhao Y., Wu H., Jin H. (2022). Active Control of Interface Dynamics in NASICON-Based Rechargeable Solid-State Sodium Batteries. Nano Lett..

[210] Cheng Y., Li M., Yang X., Lu X., Wu D., Zhang Q., Zhu Y., Gu M. (2022). Na–K Alloy Anode for High-Performance Solid-State Sodium Metal Batteries. Nano Lett..

[211] Xie G., Tang M., Xu S., Brown A., Sang L. (2022). Degradation at the Na3SbS4/Anode Interface in an Operating All-Solid-State Sodium Battery. ACS Appl. Mater. Interfaces.

[212] Shen F.C., Ma Q., Tietz F., Kao J.C., Huang C.T., Hernandha R.F.H., Huang C.W., Lo Y.C., Chang J.K., Wu W.W. (2023). In Situ Atomic-Scale Investigation of Structural Evolution During Sodiation/Desodiation Processes in Na_3_V_2_(PO_4_)_3_-Based All-Solid-State Sodium Batteries. Adv. Sci..

[213] Ruan J., Luo S., Wang S., Hu J., Fang F., Wang F., Chen M., Zheng S., Sun D., Song Y. (2023). Enhancing the Whole Migration Kinetics of Na+ in the Anode Side for Advanced Ultralow Temperature Sodium-Ion Hybrid Capacitor. Adv. Energy Mater..

[214] Song P., Chen S., Guo J., Wu J., Lu Q., Xie H., Wang Q., Liu T. (2026). Electrostatic Regulation of Na^+^ Coordination Chemistry for High-Performance All-Solid-State Sodium Batteries. Nano-Micro Lett..

[215] Zhao B., Liu J., Fan X., Guo Y., Cheng L., Zhang Y., Shao A., Tang J., Wang H., Li C. (2025). Integrated Solid Sodium Metal Battery with Reinforced Polymer Electrolyte and Presodiated 3D InSb@Cu Anode. Adv. Energy Mater..

[216] Helaley A., Liang X. (2025). Solid-state sodium-ion batteries with composite polymer electrolytes and ALD-modified Na_0.7_MnO_2_ cathodes. Chem. Eng. J..

[217] Yang D., Zhou H., Wang B., Ren K., Liu P., Zhou Y., Zhang D., Hou S., Li F., Hou M. (2025). Back-Adhesive Hybrid Solid Electrolyte with Regulated Surface Adhesion Realizing Enhanced Interfacial Kinetics and Stability in Solid-State Sodium Metal Batteries. ACS Energy Lett..

[218] Oh J.A.S., Sun J., Goh M., Chua B., Zeng K., Lu L. (2021). A Robust Solid–Solid Interface Using Sodium–Tin Alloy Modified Metallic Sodium Anode Paving Way for All-Solid-State Battery. Adv. Energy Mater..

[219] Wu E.A., Banerjee S., Tang H., Richardson P.M., Doux J.M., Qi J., Zhu Z., Grenier A., Li Y., Zhao E. (2021). A stable cathode-solid electrolyte composite for high-voltage, long-cycle-life solid-state sodium-ion batteries. Nat. Commun..

[220] Liu Y., Gao A., Hao J., Li X., Ling J., Yi F., Li Q., Shu D. (2023). Soaking-free and self-healing hydrogel for wearable zinc-ion batteries. Chem. Eng. J..

[221] Zhu R., Yang H., Cui W., Fadillah L., Huang T., Xiong Z., Tang C., Kowalski D., Kitano S., Zhu C. (2022). High strength hydrogels enable dendrite-free Zn metal anodes and high-capacity Zn–MnO_2_ batteries via a modified mechanical suppression effect. J. Mater. Chem. A.

[222] Hong L., Wu X., Liu Y.S., Yu C., Liu Y., Sun K., Shen C., Huang W., Zhou Y., Chen J.S. (2023). Self-Adapting and Self-Healing Hydrogel Interface with Fast Zn^2+^ Transport Kinetics for Highly Reversible Zn Anodes. Adv. Funct. Mater..

[223] Yan Y., Duan S., Liu B., Wu S., Alsaid Y., Yao B., Nandi S., Du Y., Wang T.W., Li Y. (2023). Tough Hydrogel Electrolytes for Anti-Freezing Zinc-Ion Batteries. Adv. Mater..

[224] Peng M., Tang X., Xiao K., Hu T., Yuan K., Chen Y. (2023). Polycation-Regulated Electrolyte and Interfacial Electric Fields for Stable Zinc Metal Batteries. Angew. Chem. Int. Ed..

[225] Li M., Xi C., Wang X., Li L., Xiao Y., Chao Y., Zheng X., Liu Z., Yu Y., Yang C. (2023). Spontaneous Desaturation of the Solvation Sheath for High-Performance Anti-Freezing Zinc-Ion Gel-Electrolyte. Small.

[226] Wang Y., Yang L., Xu P., Liu L., Li S., Zhao Y., Qin R., Pan F. (2024). An Electrochemically Initiated Self-Limiting Hydrogel Electrolyte for Dendrite-Free Zinc Anode. Small.

[227] Nian Q., Zhang X., Feng Y., Liu S., Sun T., Zheng S., Ren X., Tao Z., Zhang D., Chen J. (2021). Designing Electrolyte Structure to Suppress Hydrogen Evolution Reaction in Aqueous Batteries. ACS Energy Lett..

[228] Qin R., Wang Y., Zhang M., Wang Y., Ding S., Song A., Yi H., Yang L., Song Y., Cui Y. (2021). Tuning Zn^2+^ coordination environment to suppress dendrite formation for high-performance Zn-ion batteries. Nano Energy.

[229] Zou P., Lin R., Pollard T.P., Yao L., Hu E., Zhang R., He Y., Wang C., West W.C., Ma L. (2022). Localized Hydrophobicity in Aqueous Zinc Electrolytes Improves Zinc Metal Reversibility. Nano Lett..

[230] Feng D., Jiao Y., Wu P. (2023). Proton-Reservoir Hydrogel Electrolyte for Long-Term Cycling Zn/PANI Batteries in Wide Temperature Range. Angew. Chem. Int. Ed..

[231] Wang Y., Li Q., Hong H., Yang S., Zhang R., Wang X., Jin X., Xiong B., Bai S., Zhi C. (2023). Lean-water hydrogel electrolyte for zinc ion batteries. Nat. Commun..

[232] Wang A., Zhou W., Huang A., Chen M., Tian Q., Chen J. (2021). Developing improved electrolytes for aqueous zinc-ion batteries to achieve excellent cyclability and antifreezing ability. J. Colloid Interface Sci..

[233] Yang J.L., Li J., Zhao J.W., Liu K., Yang P., Fan H.J. (2022). Stable Zinc Anodes Enabled by a Zincophilic Polyanionic Hydrogel Layer. Adv. Mater..

[234] Sun M., Ji G., Zheng J. (2023). A hydrogel electrolyte with ultrahigh ionic conductivity and transference number benefit from Zn^2+^ “highways” for dendrite-free Zn-MnO_2_ battery. Chem. Eng. J..

[235] Li Z., Zhou G., Ye L., He J., Xu W., Hong S., Chen W., Li M.C., Liu C., Mei C. (2023). High-energy and dendrite-free solid-state zinc metal battery enabled by a dual-network and water-confinement hydrogel electrolyte. Chem. Eng. J..

[236] Chen Z.J., Shen T.Y., Zhang M.H., Xiao X., Wang H.Q., Lu Q.R., Luo Y.L., Jin Z., Li C.H. (2024). Tough, Anti-Fatigue, Self-Adhesive, and Anti-Freezing Hydrogel Electrolytes for Dendrite-Free Flexible Zinc Ion Batteries and Strain Sensors. Adv. Funct. Mater..

[237] Sun M., Ji G., Li M., Zheng J. (2024). A Robust Hydrogel Electrolyte with Ultrahigh Ion Transference Number Derived from Zincophilic “Chain-Gear” Network Structure for Dendrite-Free Aqueous Zinc Ion Battery. Adv. Funct. Mater..

[238] Li J., Zhang H., Liu Z., Du H., Wan H., Li X., Yang J., Yan C. (2025). Boosting Dendrite-Free Zinc Anode with Strongly Polar Functional Group Terminated Hydrogel Electrolyte for High-Safe Aqueous Zinc-Ion Batteries. Adv. Funct. Mater..

[239] Gu Y., Zheng X., Zhou Z., Chen G., Chen S., Li Q. (2024). Amphiphilic ionic liquid hydrogel electrolytes with high ionic conductivity towards dendrite-free ultra-stable aqueous zinc ion batteries. J. Energy Storage.

[240] Xu D., Wang Y., Tian H., Chen Y., Tian X., Zhang Q. (2025). Dynamic Cross-Linking Network Construction of Flexible Hydrogel Electrolyte Enabling Dendrite-Free Zinc Anode. Adv. Energy Mater..

[241] Wu R., Yang S., Wang R., Guo Y., Du P. (2025). Pullulan enhanced dual-network hydrogel electrolyte for high-performance flexible zinc-ion batteries. Chem. Eng. J..

[242] Somteds A., Tayraukham P., Pipattanachaiyanan P., Kao-ian W., Wannapaiboon S., Loiha S., Sukpirom N., Suttipong M., Guo J., Kheawhom S. (2025). CO_2_-Derived Polymeric Double-Network Hydrogel Electrolyte for Zinc-Ion Batteries. ACS Sustain. Chem. Eng..

[243] Yu J., Li M., Kong X., Wang T., Zhang H., Zhu X., Zhao J., Ma Z., Yang H. (2025). Polyanion hydrogel electrolyte with a high Zn 2+ transference number for dendrite-free aqueous zinc-ion batteries. J. Mater. Chem. A.

[244] Zeng J., Chen H., Dong L., Guo X. (2024). Anti-Polyelectrolyte Effect of Zwitterionic Hydrogel Electrolytes Enabling High-Voltage Zinc-Ion Hybrid Capacitors. Adv. Funct. Mater..

[245] Zhang Z., Zhang R., Gao Y., Gao Y., Jia F., Gao G. (2024). Dendrite-free zinc anode enabled by zwitterionic organic hydrogel electrolytes for high-voltage zinc-ion hybrid capacitors. Chem. Eng. J..

[246] Yang C., Woottapanit P., Geng S., Chanajaree R., Shen Y., Lolupiman K., Limphirat W., Pakornchote T., Bovornratanaraks T., Zhang X. (2025). A multifunctional quasi-solid-state polymer electrolyte with highly selective ion highways for practical zinc ion batteries. Nat. Commun..

[247] Xu J., Shen H., Gao K., Xu Z., Liu S., Liang X., Zhou W., Jia J., Huang H., Zhu Y. (2026). Trace-MXene-induced electrolyte reconfiguration and interfacial regulation for high-performance aqueous zinc-ion batteries. Chem. Eng. J..

[248] Zhang Y., Wen G., Zhang J., Qin Y., Cui W., Luo Z., Huang B., Yu J., Wang X. (2024). A lignocellulose-based hydrogel polymer electrolytes with high conductivity and stability for rechargeable flexible zinc-air batteries. J. Energy Storage.

[249] Li C.Y., Wang J.L., Zhang D.T., Li M.P., Chen H., Yi W.H., Ren X.Y., Liu B., Lu X.F., Liu M.C. (2024). In-situ physical/chemical cross-linked hydrogel electrolyte achieving ultra-stable zinc anode-electrolyte interface towards dendrite-free zinc ion battery. J. Energy Chem..

[250] Wang N., Liu H., Sun M., Ren X., Hu L., Li Z., Yao X., Gong Y., Jia C. (2024). Achieving Wide-Temperature-Range Sustainable Zinc-Ion Batteries via Magnesium-Doped Cathodes and Gel Electrolytes. ACS Sustain. Chem. Eng..

[251] Zhang B., Cai X., Li J., Zhang H., Li D., Ge H., Liang S., Lu B., Zhao J., Zhou J. (2024). Biocompatible and stable quasi-solid-state zinc-ion batteries for real-time responsive wireless wearable electronics. Energy Environ. Sci..

[252] Xia H., Zhang W., Miao C., Chen H., Yi C., Shang Y., Shui T., Cao X., Liu J., Kure-Chu S.Z. (2024). Ultra-thin amphiphilic hydrogel electrolyte for flexible zinc-ion paper batteries. Energy Environ. Sci..

[253] Chen T., Shuang Z., Hu J., Zhao Y., Wei D., Ye J., Zhang G., Duan H. (2022). Freestanding 3D Metallic Micromesh for High-Performance Flexible Transparent Solid-State Zinc Batteries. Small.

[254] Tan Y., Liao R., Mu Y., Dong L., Chen X., Xue Y., Zheng Z., Wang F., Ni Z., Guo J. (2024). Hierarchically-Structured and Mechanically-Robust Hydrogel Electrolytes for Flexible Zinc-Iodine Batteries. Adv. Funct. Mater..

[255] Shen Z., Liu Y., Li Z., Tang Z., Pu J., Luo L., Ji Y., Xie J., Shu Z., Yao Y. (2025). Highly-Entangled Hydrogel Electrolyte for Fast Charging/Discharging Properties in Aqueous Zinc Ion Batteries. Adv. Funct. Mater..

[256] Zhang J., Lin C., Zeng L., Lin H., He L., Xiao F., Luo L., Xiong P., Yang X., Chen Q. (2024). A Hydrogel Electrolyte with High Adaptability over a Wide Temperature Range and Mechanical Stress for Long-Life Flexible Zinc-Ion Batteries. Small.

[257] Yang Z., Wu W., Duan M., Chen S., Liu M., Liu J., Huang T., Liu H. (2025). Strategies for Advanced Solid Electrolytes toward Efficient Lithium-Ion Conduction in All-Solid-State Lithium Metal Batteries. ACS Appl. Mater. Interfaces.

[258] Fong K.D., Grey C.P., Michaelides A. (2025). On the Physical Origins of Reduced Ionic Conductivity in Nanoconfined Electrolytes. ACS Nano.

[259] Wang S., Fu J., Liu Y., Saravanan R.S., Luo J., Deng S., Sham T.K., Sun X., Mo Y. (2023). Design principles for sodium superionic conductors. Nat. Commun..

[260] Li L., Shan Y., Wang F., Chen X., Zhao Y., Zhou D., Wang H., Cui W. (2021). Improving Fast and Safe Transfer of Lithium Ions in Solid-State Lithium Batteries by Porosity and Channel Structure of Polymer Electrolyte. ACS Appl. Mater. Interfaces.

[261] Uchida K., Ohkubo T., Utsuno F., Yazawa K. (2021). Modified Li7P3S11 Glass-Ceramic Electrolyte and Its Characterization. ACS Appl. Mater. Interfaces.

[262] Tsai C.Y., Liu Y.L. (2022). Building up ion-conduction pathways in solid polymer electrolytes through surface and pore functionalization of PVDF porous membranes with ionic conductors. J. Membr. Sci..

[263] Zhang D., Liu Y., Sun Z., Liu Z., Xu X., Xi L., Ji S., Zhu M., Liu J. (2023). Eutectic-Based Polymer Electrolyte with the Enhanced Lithium Salt Dissociation for High-Performance Lithium Metal Batteries. Angew. Chem. Int. Ed..

[264] Kravchyk K.V., Zhang H., Okur F., Kovalenko M.V. (2022). Li–Garnet Solid-State Batteries with LLZO Scaffolds. Acc. Mater. Res..

[265] Zhang L., Gao H., Xiao S., Li J., Ma T., Wang Q., Liu W., Wang S. (2022). In-Situ Construction of Ceramic–Polymer All-Solid-State Electrolytes for High-Performance Room-Temperature Lithium Metal Batteries. ACS Mater. Lett..

[266] Tian L.W., Kim J.W., Kim D.W. (2023). Solid Hybrid Electrolyte Featuring a Vertically Aligned Li6.4La3.0Zr1.4Ta0.6O12 Membrane for All-Solid-State Lithium Batteries. ACS Appl. Mater. Interfaces.

[267] Maurya D.K., Bazri B., Srivastava P., Huang J.Y., Hung Y.T., Huang W.T., Wei D.H., Liu R.S. (2024). Ceramic Rich Composite Electrolytes: An Overview of Paradigm Shift toward Solid Electrolytes for High-Performance Lithium-Metal Batteries. Adv. Energy Mater..

[268] Zhang Y., Zhang L., Guo P., Zhang C., Ren X., Jiang Z., Song J., Shi C. (2024). Porous garnet as filler of solid polymer electrolytes to enhance the performance of solid-state lithium batteries. Nano Res..

[269] Wei B., Li Y., Lin W., Yu J., Chen D. (2024). A wrapped and infiltrated ~20-μm-thick 3D ceramic framework composite enables fast Li+ diffusion and interfacial compatibility for lithium-metal batteries. Compos. Part B Eng..

[270] Chen Q., Ouyang C., Liang Y., Liu H., Duan H. (2024). Composite polymer electrolyte with vertically aligned garnet scaffolds for quasi solid-state lithium batteries. Energy Storage Mater..

[271] Xu Z., Lin C., Qiu J., Wang Z. (2025). Polymer-Regulated Solvation and Interphase Engineering for Long-Life and Safe Quasi-Solid-State Anode-Free Sodium Batteries. Adv. Mater..

[272] Chen W., Guo S., Qin L., Li L., Cao X., Zhou J., Luo Z., Fang G., Liang S. (2022). Hydrogen Bond-Functionalized Massive Solvation Modules Stabilizing Bilateral Interfaces. Adv. Funct. Mater..

[273] Shi X., Zhong Y., Yang Y., Zhou J., Cao X., Liang S. (2025). Anion-Anchored Polymer-in-Salt Solid Electrolyte for High-Performance Zinc Batteries. Angew. Chem. Int. Ed..

[274] Wen J., Qiu B., Guan Y., Zhao R., Xiao G., He C., Zhang P., He Y.B., Mi H. (2026). Defect-Driven Ionic Trap Construction and Interface Modulation for Rapid Li^+^ Kinetics in Composite Solid Electrolytes. Adv. Mater..

[275] Zhang M., Li J.H., Tang Y., Wang D.W., Hu H., Liu M., Xiao B., Wang P.F. (2024). Selective Zn-ion channels enabled by a double-network protective layer for stable zinc anode. Energy Storage Mater..

[276] Cai W., Zhang X., Li G., Chen L. (2024). Tailoring codirectional Zn2+ pathways with biomaterials for advanced hydrogel electrolytes in High-Performance zinc metal batteries. Chem. Eng. J..

[277] Zhang K., Xie S., Liang J., Zheng Z., Li Y., Dong L. (2025). Functional ultrathin separators enabling stable zinc anodes for lean-electrolyte zinc-ion batteries. J. Membr. Sci..

[278] Alregeb F., Khalili F., Sweileh B., Ali D.K. (2022). Synthesis and Characterization of Chelating Hyperbranched Polyester Nanoparticles for Cd(II) Ion Removal from Water. Molecules.

[279] Song Y., Yang L., Li J., Zhang M., Wang Y., Li S., Chen S., Yang K., Xu K., Pan F. (2021). Synergistic Dissociation-and-Trapping Effect to Promote Li-Ion Conduction in Polymer Electrolytes via Oxygen Vacancies. Small.

[280] Paulo G., Sun K., Di Muccio G., Gubbiotti A., Morozzo della Rocca B., Geng J., Maglia G., Chinappi M., Giacomello A. (2023). Hydrophobically gated memristive nanopores for neuromorphic applications. Nat. Commun..

[281] Alvarez-Fernandez A., Hernández G., Maiz J. (2025). Unlocking Solid Polymer Electrolytes: Advancing Materials through Characterization-Driven Insights. JACS Au.

[282] Chen Y., Yang T., Lin Y., Evans C.M. (2025). Ion transport in helical-helical polypeptide polymerized ionic liquid block copolymers. Nat. Commun..

[283] Tsai P.C., Mair S., Smith J., Halat D.M., Chien P.H., Kim K., Zhang D., Li Y., Yin L., Liu J. (2023). Double Paddle-Wheel Enhanced Sodium Ion Conduction in an Antiperovskite Solid Electrolyte. Adv. Energy Mater..

[284] Ratschow A.D., Wagner A.J., Janssen M., Hardt S. (2025). Convection can enhance the capacitive charging of porous electrodes. Proc. Natl. Acad. Sci. USA.

[285] Tan H., Tan T., Jiang Y., Bai H., Zhou R., Feng J. (2026). Nonaqueous Ion Transport through Nanopores: A Nonlinear Behavior Driven by Enhanced Ion Correlation. J. Am. Chem. Soc..

[286] Li Z., Xu Y., Lu W., Pan X., Wu L., Ong W.L., Shpigel N., Chen G., Ho G.W. (2026). Ion transport control in electrolytes via electrochemical doping. Proc. Natl. Acad. Sci. USA.

[287] Wang M., Xiong Q., Yue X., Yan G., Han Y., Lyu Z., Li Z., Sun L., Hoenig E., Xu K. (2025). Cooperative and inhibitory ion transport in functionalized angstrom-scale two-dimensional channels. Nat. Commun..

[288] Shang Z., Zhao J., Yang M., Xiao Y., Chu W., Xu S., Zhang X., Yi X., Lin M., Xia F. (2024). Precise control of transmembrane current via regulating bionic lipid membrane composition. Sci. Adv..

[289] Luo J., Remy A., Zhang Y. (2025). Iontronic Devices from Biological Nanopores to Artificial Systems: Emerging Applications and Future Perspectives. Chem. Rev..

[290] Tsutsui M., Hsu W.L., Garoli D., Douaki A., Komoto Y., Daiguji H., Kawai T. (2026). Chemistry-driven autonomous nanopore membranes. Nat. Commun..

[291] Mayer S.F., Mitsioni M.F., Robin P., van den Heuvel L., Ronceray N., Marcaida M.J., Abriata L.A., Krapp L.F., Anton J.S., Soussou S. (2026). Lumen charge governs gated ion transport in β-barrel nanopores. Nat. Nanotechnol..

[292] Li C., Chen C., Shi J., Han W., Fu Q., Yang L. (2026). Biomimetic action-potential transmission via mechano-gated potassium channels assembled by quadruple hydrogen-bonded crown ethers. Sci. Adv..

[293] Emmerich T., Teng Y., Ronceray N., Lopriore E., Chiesa R., Chernev A., Artemov V., Di Ventra M., Kis A., Radenovic A. (2024). Nanofluidic logic with mechano–ionic memristive switches. Nat. Electron..

[294] Noh Y., Smolyanitsky A. (2024). Synaptic-like plasticity in 2D nanofluidic memristor from competitive bicationic transport. Sci. Adv..

[295] Baram D., Kvetny M., Ake S., Yang R., Wang G. (2025). Anodized Aluminum Oxide Membrane Ionic Memristors. J. Am. Chem. Soc..

[296] Cho A.D., Wawrzkiewicz-Jałowiecka A., Dewi C.E.P., Tang S., Cain D., Cao E., Martens C., Schäffer T.E., Cervera J., Ramirez P. (2025). Nanopores with Ionic Memory in Oscillating Ion Current Signals. J. Am. Chem. Soc..

[297] Farajpour N., Bandara Y.M.N.D.Y., Lastra L., Freedman K.J. (2025). Negative memory capacitance and ionic filtering effects in asymmetric nanopores. Nat. Nanotechnol..

[298] Yadav S., Gogoi R.K., Lokhandwala A., Pandey S.V., Bhardwaj A., DasGupta S., Boya R. (2026). Two-dimensional clay channels for tunable nanofluidic memristor. Commun. Mater..

[299] Cao E., Cain D., Silva S., Siwy Z.S. (2024). Ion Concentration Polarization Tunes Interpore Interactions and Transport Properties of Nanopore Arrays. Adv. Funct. Mater..

[300] Tsutsui M., Hsu W.L., Hsu C., Garoli D., Weng S., Daiguji H., Kawai T. (2025). Transmembrane voltage-gated nanopores controlled by electrically tunable in-pore chemistry. Nat. Commun..

[301] Liao S., Liu Y., Li L., Ding L., Wei Y., Wang H. (2025). Theoretical framework for confined ion transport in two-dimensional nanochannels. Nat. Commun..

[302] Hu X., Xu H., Lu J., Cui F., Wu H.A., Wang F., Jiang L., Novoselov K.S., Wang H. (2025). Selective ion transport of nonlinear resistive switching by hierarchical nanometer-to-angstrom channels for nanofluidic transistors. Sci. Adv..

[303] Qian H., Fan H., Peng P., Du Y., Li X., Liu Y., Yang F., Zhou Y., Wang Z.L., Wei D. (2025). Biomimetic Janus MXene membrane with bidirectional ion permselectivity for enhanced osmotic effects and iontronic logic control. Sci. Adv..

[304] Poletayev A.D., Hoffmann M.C., Dawson J.A., Teitelbaum S.W., Trigo M., Islam M.S., Lindenberg A.M. (2024). The persistence of memory in ionic conduction probed by nonlinear optics. Nature.

[305] Pandey S.V., Saurav K.V., Goutham S., Sharma A., Keerthi A., Radha B. (2026). Ionic memory or electrode artefacts? A systematic assessment of nanofluidic memristors. Faraday Discuss..

[306] Yakovtseva V., Volchek S., Bondarenko V., Sayyed M.I., Hanafy T.A., Trukhanov S., Bondaruk A., Rotkovich A., Silibin M.V., Zubar T. (2024). Effects during the cathode polarization of porous silicon. Heliyon.

[307] Puls S., Nazmutdinova E., Kalyk F., Woolley H.M., Thomsen J.F., Cheng Z., Fauchier-Magnan A., Gautam A., Gockeln M., Ham S.Y. (2024). Benchmarking the reproducibility of all-solid-state battery cell performance. Nat. Energy.

[308] Hiraoka K., Yamamoto K., Kobayashi T., Sakamoto T., Seki S. (2025). Multi-Scale Analysis Combined Operando Elemental/Spectroscopic Measurement Techniques in Oxide-Type All-Solid-State Na Batteries. Energy Environ. Mater..

[309] Xue T., Mu Y., Zhang Z., Guan J., Qiu J., Yang C., Zang L., Zeng L. (2025). Enhanced Zinc Deposition and Dendrite Suppression in Aqueous Zinc-Ion Batteries Via Citric Acid-Aspartame Electrolyte Additives. Adv. Energy Mater..

[310] Wei X., Mu Y., Chen J., Zhou Y., Chu Y., Yang L., Huang C., Xue T., Zang L., Yang C. (2025). Optimizing Zn (100) deposition via crystal plane shielding effect towards ultra-high rate and stable zinc anode. Energy Storage Mater..

[311] Mu Y., Zhou Y., Chu Y., Wei X., Gu H., Fang J., Liao R., Wu F., Zhang Q., He G. (2025). Robust Piezoelectric-Derived Bilayer Solid Electrolyte Interphase for Zn Anodes Operating from −60 to 60 °C. ACS Nano.

[312] Kim D., Hu X., Yu B., Chen Y.I. (2024). Small Additives Make Big Differences: A Review on Advanced Additives for High-Performance Solid-State Li Metal Batteries. Adv. Mater..

[313] Yin X., Guo Y., Chi S., Jia Y., Li F., Qi J., Yi X., Wu S., Yang Q.H. (2025). Beyond Polymerization: In Situ Coupled Fluorination Enables More Stable Interfaces for Solid-State Lithium Batteries. J. Am. Chem. Soc..

